# The large milkweed bugs’ Na,K-ATPase β-subunits colocalize with septate junction proteins in a tissue-specific manner

**DOI:** 10.1007/s00441-025-03965-3

**Published:** 2025-03-26

**Authors:** Marlena Herbertz, Christian Lohr, Susanne Dobler

**Affiliations:** 1 Institute of Cell and Systems Biology of Animals, Molecular Evolutionary Biology, University of Hamburg, 20146 Hamburg, Germany; 2 Institute of Cell and Systems Biology of Animals, Neurophysiology, University of Hamburg, 20146 Hamburg, Germany

**Keywords:** Cell–cell contacts, Discs large, Coracle, Paracellular barrier, *Oncopeltus fasciatus*

## Abstract

The Na,K-ATPase is a vital transmembrane enzyme, which is important for maintaining cell membrane potentials and the general functionality of animal cells. The enzyme’s minimal functional unit consists of one α and one β-subunit, whereas the number of existing paralogs varies in different insect species. The functional roles of different β-subunits, which can account for their diversity within a single species, are so far only partially explained. The emphasis of this study was to specifically elucidate the involvement in septate junctions of the four β-subunits of the new model system *Oncopeltus fasciatus*. Septate junctions function as a paracellular barrier controlling the flow of solutes across epithelia. So far, studies in *Drosophila* revealed that nervana2, the β2 homolog of *Drosophila*, is involved in septate junction formation. In *O. fasciatus*, we demonstrate that most of the Na,K-ATPase subunits colocalize with septate junction proteins. This agrees with our previous findings implying a role of β2 in the control of tracheal tube size in *O. fasciatus*, which according to the findings in *Drosophila* appears to be dependent on a stable formation of septate junctions. Finally, our data suggest a connection between the septate junction protein coracle and the enigmatic, N-terminally strongly truncated βx, which has no obvious homologs in other insects. Our study proposes that the four β-subunits form functional units with septate junction proteins, either allowing tissue-adjusted formation of cell–cell contacts or other yet unknown functions.

**Supplementary Information:**

The online version contains supplementary material available at 10.1007/s00441-025-03965-3.

## Introduction

The Na,K-ATPase (NKA) is a vital transmembrane enzyme expressed in all animal cells. Despite massive research efforts since its discovery in 1957 (Skou 1957), our understanding of this enzyme’s function remains incomplete. Unquestionably, the NKA is a vital enzyme important for maintaining membrane potentials by transporting two K^+^ against three Na^+^ ions across cell membranes through the hydrolysis of ATP. In addition, it is important for the excitability of cells, osmoregulation, cell volume and pH homeostasis, and electrolyte balance (Horisberger 2004; Pierre and Xie 2006; Skou and Esmann 1992). All across the animal kingdom, the enzyme is very specifically inhibited by cardiotonic steroids, which include cardiac glycosides. These toxins bind to a specific pocket located in the catalytic α-subunit and thereby block ion exchange (Horisberger 2004) with serious health consequences for the organisms that can lead to death. Crystallization of the vertebrate NKA revealed further insights into its function (Laursen et al. 2013; Ogawa et al. 2009; Yatime et al. 2011), and accumulating evidence corroborated its role in intracellular signaling cascades, likely initiated by endogenous cardiac glycosides or a similar compound (Askari 2019; Cui and Xie 2017; Liang et al. 2006; Lingrel 2010; Pierre and Blanco 2021). In addition, a pump-independent role of the enzyme in the formation and function of tight and septate junctions emerged in vertebrates (Krupinski and Beitel 2009; Rajasekaran and Rajasekaran 2009; Rajasekaran et al. 2001) and in *Drosophila* (Genova and Fehon 2003; Oshima and Fehon 2011).

Junctions determine cell–cell contacts, membrane barriers, and polarity of cells. Junctions include desmosomes, hemidesmosomes, gap junctions, adherent junctions, tight junctions (TJs), and septate junctions (SJs), but they are not all ubiquitously present in tissues across the animal kingdom (Tepass and Hartenstein 1994; Tepass et al. 2001). For paracellular barrier function, SJs in invertebrates are the functional counterpart of vertebrate TJs, although their cellular orientation differs. The TJs are located at the apical cell border, whereas in invertebrates, adherent junctions build the uppermost cell–cell contacts followed by SJs (Rajasekaran and Rajasekaran 2009). TJs and SJs function both as a paracellular barrier controlling leakage of solutes and pathogens across epithelia (Banerjee et al. 2006; Oshima and Fehon 2011; Rajasekaran and Rajasekaran 2009). Both types of junctions are formed by a protein complex, and invertebrates carry homologs of the complex-forming proteins found in vertebrates. For example, neuroglian is the invertebrate homolog of the vertebrate neurofascin, coracle is a protein 4.1 homolog, and gliotactin is a neuroligin 3 protein homolog (Genova and Fehon 2003).

In addition to numerous other proteins involved in SJ formation and functionality, the NKA forms part of the TJ and SJ protein complexes (Rajasekaran and Rajasekaran 2009). The NKA β2 homolog of *Drosophila* nervana2 (nrv2) and α1 were found together with neurexin IV, neuroglian, gliotactin, and coracle to build an interdependent complex that is essential for the formation of a paracellular barrier (Genova and Fehon 2003; Oshima and Fehon 2011). Additionally, proteins of the lethal giant larva (lgl) group such as discs large (dlg) and scribbled are known to be important for cell polarity and their connection to the SJ protein complex (Bilder et al. 2000; Oshima and Fehon 2011; Pradhan et al. 2023; Wu and Beitel 2004). They are so-called SJ resident proteins that physically reside in SJs but are not needed for their formation or maintenance (Rice et al. 2021). Besides the barrier function, SJs were found to be involved in tracheal tube size control. Mutations in n*rv2*, *α1*, and other SJ complex genes resulted in tracheal tube size alterations (Paul et al. 2003). Although both NKA subunits, α1 and β, were determined to be part of the SJ protein complex, very little is known about the role of the smaller, “chaperone-like” β-subunit. Furthermore, previous studies on SJs in invertebrates exclusively focus on *Drosophila*.

Here, we focused on a cardiac glycoside-resistant hemimetabolous insect, the large milkweed bug *Oncopeltus fasciatus*, which is a specialist feeder on cardiac glycoside-defended plants*.* Due to multiple gene duplications, four α1 paralogs (A, B, C, D) exist (Yang et al. 2019; Zhen et al. 2012) that differ in activity and resistance against cardiac glycosides (Dalla et al. 2017; Herbertz et al. 2024; Lohr et al. 2017). Additionally, four β-subunits are present (β1, β2, β3, βx) that can build different complexes with the α1 paralogs. They show different distribution patterns and play different functional roles (Herbertz et al. 2022, 2023, 2024).

In a previous study, we found that tracheal tube morphology changed significantly after the knockdown of β2 in *O. fasciatus*. Specifically, the distances between taenidiae decreased significantly, indicating alterations in tube length (Herbertz et al. 2023). This resembles findings in earlier studies, where NKA-mutant fruit flies showed stark alterations in tracheal morphology (Paul et al. 2003, 2007). Together, these findings suggest a conserved role of specific NKA subunits in tracheal morphology control. Tube length and diameter are adjusted to the physical needs of an organism. Alterations, such as expanded tube length, might lead to a slower airflow and consequently insufficient oxygen supply. During the development of the fruit fly, the expansion of the tracheal tubes concurs with the SJ complex assembly (Beitel and Kransnow 2000). Therefore, disrupted SJ formation may directly induce alterations in tracheal tube development (Paul et al. 2007). To disentangle the connection of the NKA in tracheal tube size control and SJ formation in *O. fasciatus*, we here investigated whether and to what extent the four different β-subunits colocalize with the SJ proteins coracle and dlg and whether there are differences between tissues regarding amount and identity of the colocalizing proteins. To address these goals, we performed immunohistochemistry (IHC) analyses lined with LC–MS/MS data. Our results contribute to our understanding of NKA involvement in SJs and elucidate a connection between different NKA β-subunits and the SJ proteins.

## Material and methods

### Identifying coracle and discs large homologs

We investigated coracle and discs large because they were determined to be part of the SJs in *Drosophila* by Fehon et al. (1994) and important for cell–cell contacts and the normal development of the organisms. The discs large protein sequence (UniProt: P31007 DLG1_DROME Disks large 1 tumor suppressor protein, *Drosophila melanogaster*) and the coracle protein sequence, also known as protein 4.1 homolog (UniProt: Q9V8R9 (EPB41_DROME) *Drosophila melanogaster*), were used as a reference to search via tblastn (NCBI) for potential homologs in a transcriptome of *O. fasciatus* (NCBI, TSA: *Oncopeltus fasciatus* breed wildtype, transcriptome shotgun assembly, GenBank: GCXY00000000.1) using the transcriptome shotgun assembly (TSA) database. The sequences of *O. fasciatus* GCXY01049860.1 and GCXY01049861.1 strongly match the reference sequence of *D. melanogaster* in the area of the monoclonal discs large antibody 4F3 anti-discs large epitope (439–756 aa of P31007 DLG1_DROME Disks large 1 tumor suppressor protein, Fig. S2), and GCXY01047324.1 strongly matches the reference sequence of *D. melanogaster* in the area of the monoclonal coracle antibody C566.9 epitope (72–397 aa of Q9V8R9, Fig. S3), which justifies the use of the antibodies in this study.

### Rearing of large milkweed bugs

Populations of *O. fasciatus* were established in our lab as described in Herbertz et al. (2022). The individuals used here were reared on sunflower seeds at 25 °C, 50% humidity, and a 14/10 h day/night rhythm throughout the year. For consistency purposes and to allow comparability with former studies, only females were used for all analyses conducted here.

### Tissue dissection

Salivary glands and nervous tissues were dissected on ice. A total of 25 female milkweed bugs were chilled in the fridge for about 10–15 min and immediately killed by separating the thorax from the abdomen. Salivary glands and nervous tissues were dissected with ultra-fine forceps and immediately stored in a fixing solution containing 4% paraformaldehyde (Merck KGaA, Darmstadt, Germany) in phosphate-buffered saline (PBS, pH 7.4) for 24 h at 4 °C.

Afterwards, the fixed tissues were washed in PBS for 5 min. Salivary glands and nervous tissues were mounted in 5% low melt agarose (AGS GmbH, Heidelberg, Germany) and cut into 180 µm wide slices with a vibratome (VT1000S, Leica, Wetzlar, Germany) as described in Herbertz et al. (2022).

### Immunohistochemistry

The slices were washed with PBS for 5 min and blocked with 2.5% normal goat serum (Invitrogen by Thermo Fisher Scientific, Rockford, USA), 2.5% bovine serum albumin (BSA), and 0.025% Triton X (Carl Roth GmbH, Karlsruhe, Germany) in PBS for 2 h at room temperature (RT) followed by three washing steps. The slices were incubated overnight with 5 µg/ml of primary antibody (10 µg/ml of β1-specific antibody) in a 1:2 dilution of blocking solution. A double staining was performed with one of the monoclonal mouse primary antibodies anti-coracle (C566.9; DSHB Hybridoma Bank, University of Iowa; deposited by R. Fehon (Fehon et al. 1994)) or anti-discs large (4F3 anti-discs large; DSHB Hybridoma Bank, University of Iowa; deposited by C. Goodman (Parnas et al. 2001)) and one of the four affinity purified, customer-designed polyclonal β-specific primary antibodies (chicken anti-β1, rabbit anti-β2, chicken anti-β3, or rabbit anti-βx; produced by Davids Biotechnologie, Regensburg, Germany). Additional negative controls were incubated without primary antibodies in a 1:2 dilution of blocking solution. The tissues and slices were incubated for 3 days at 4 °C with a slow end-to-end movement. After three washing steps, the binding of light-sensitive secondary antibodies followed. A double staining with goat anti-mouse red fluorescent secondary antibody (Cy3; 1:200 in 1:2 diluted blocking solution; Sigma, St. Louis, MO, USA) and goat anti-chicken green fluorescent secondary antibody (Alexa 488, 1:1000 in 1:2 diluted blocking solution; Invitrogen, Carlsbad, CA, USA) or goat anti-rabbit green fluorescent secondary antibody (Alexa 488, 1:1000 in 1:2 diluted blocking solution; Invitrogen, CA, California, USA) was performed for 2 days at 4 °C with a slow end-to-end movement. After three washing steps with PBS, the slices of salivary glands and nervous tissues were incubated with 4′−6-diamidine-2-phenylindole (DAPI) (DAPI, 1:1000 in PBS, Merck, Darmstadt, Germany) for 30 min at room temperature with a slow end-to-end movement. Three times 10-min washing followed. The slices were transferred onto microscopic slides, embedded with air-hardening Shandon Immu-Mount (Thermo Scientific), covered with cover slips, and sealed the next day with nail polish.

The samples were imaged using a confocal microscope eC1 (Nikon, Düsseldorf, Germany) equipped with a × 40 lens (CFI Plan Fluor 40X Oil, NA 1.3, Nikon). The gain values for each fluorescence channel (488 green, 543 red) were restricted by the negative controls (see Fig. S1). To avoid a bleed-through effect, each channel was imaged separately.

### Image analyses

For comparisons between abundances and colocalization patterns of different β-subunits, discs large, and coracle, the IHC images were analyzed with the free software Fiji (ImageJ, version 1.54f).

The channels of each image were split, and the scale was set. Background noise was subtracted via the rolling ball algorithm with the default setting (50 pixels), and the threshold was adjusted by using the “Moments” setting. Afterwards, the images were processed to binary pictures composed of a white background and black structures. The black structures representing a target protein were selected with the function “Create Selection” and added to the region of interest (ROI) manager.

The area of each target (dlg, coracle, β) was measured. To calculate the area of two colocalizing targets, the “Image Calculator” was used by running the operation “AND.” This operation visualizes only areas in which both targets are abundant. The selection of the colocalization area was added to the ROI manager, and the area was measured.

For normalization purposes, the area of the whole tissue was measured.

### Data analyses and statistics

All measured areas of the target β-subunits, coracle, and discs large, as well as colocalizations, were normalized to the mean total area of the biological replicates (mean area/area_bio. rep._). Relative abundances of overlapping areas were expressed in percentage (analyzed colocalizations: β and discs large, β and coracle). Areas of β-subunits, discs large, and coracle were expressed in pixels/micron.

For statistics, each dataset was first checked for normal distribution (one-sample Kolmogorov–Smirnov test) and homogeneity of variances (Levene’s test). We performed an ANOVA to test for significant differences between areas of β-subunits, discs large, and coracle for each tissue separately by using the following code: aov (area ~ target, data = file name) followed by the post hoc TukeyHSD test. The results were visualized in boxplot format.

Every statistical analysis was conducted by using R version 4.4.2 (2024–10–31) (© 2024 the R Foundation for Statistical Computing).

## Results

### β-subunits colocalize with discs large and coracle in different tissues

#### Analyses of colocalization between β-subunits, discs large, and coracle

**Fig. 1:**
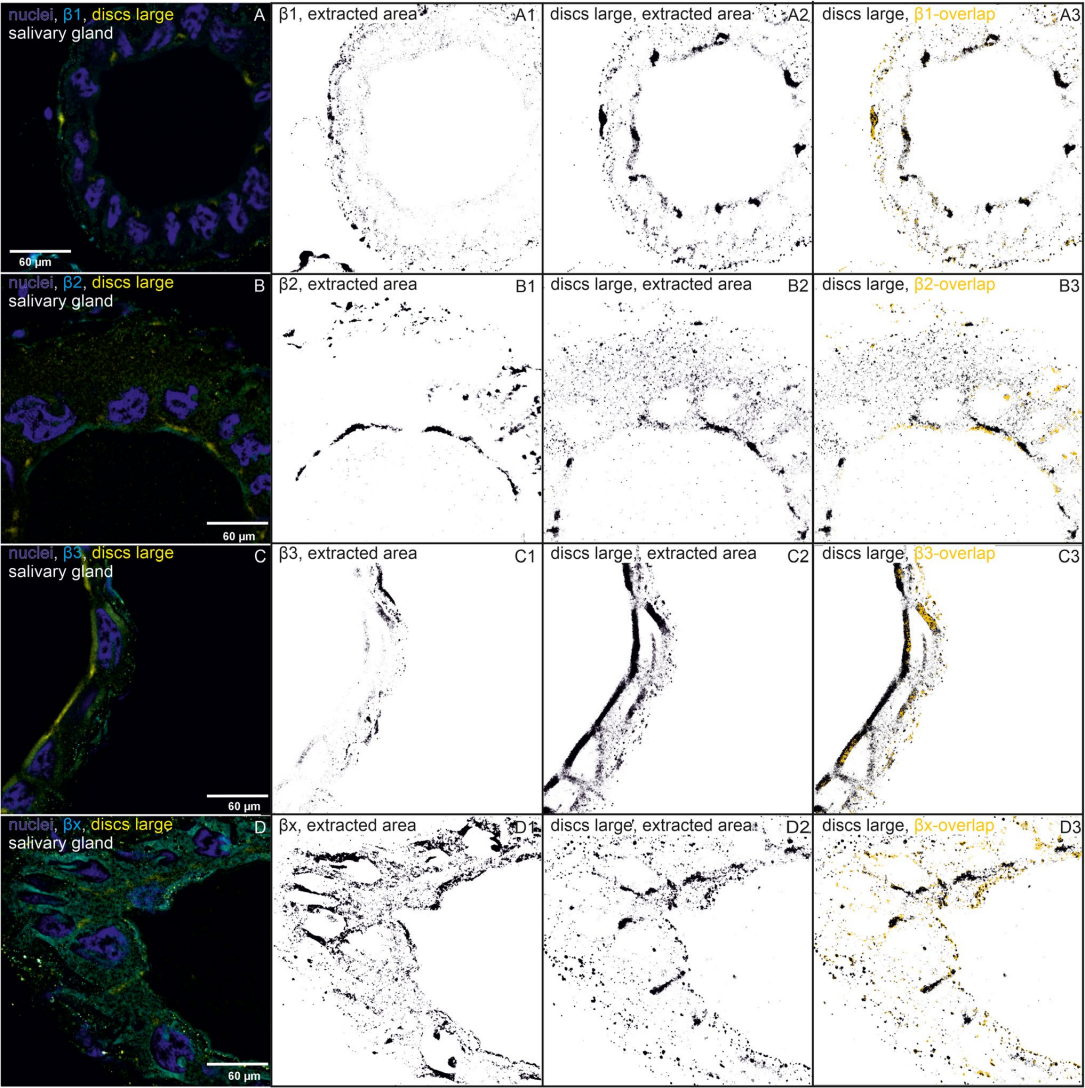

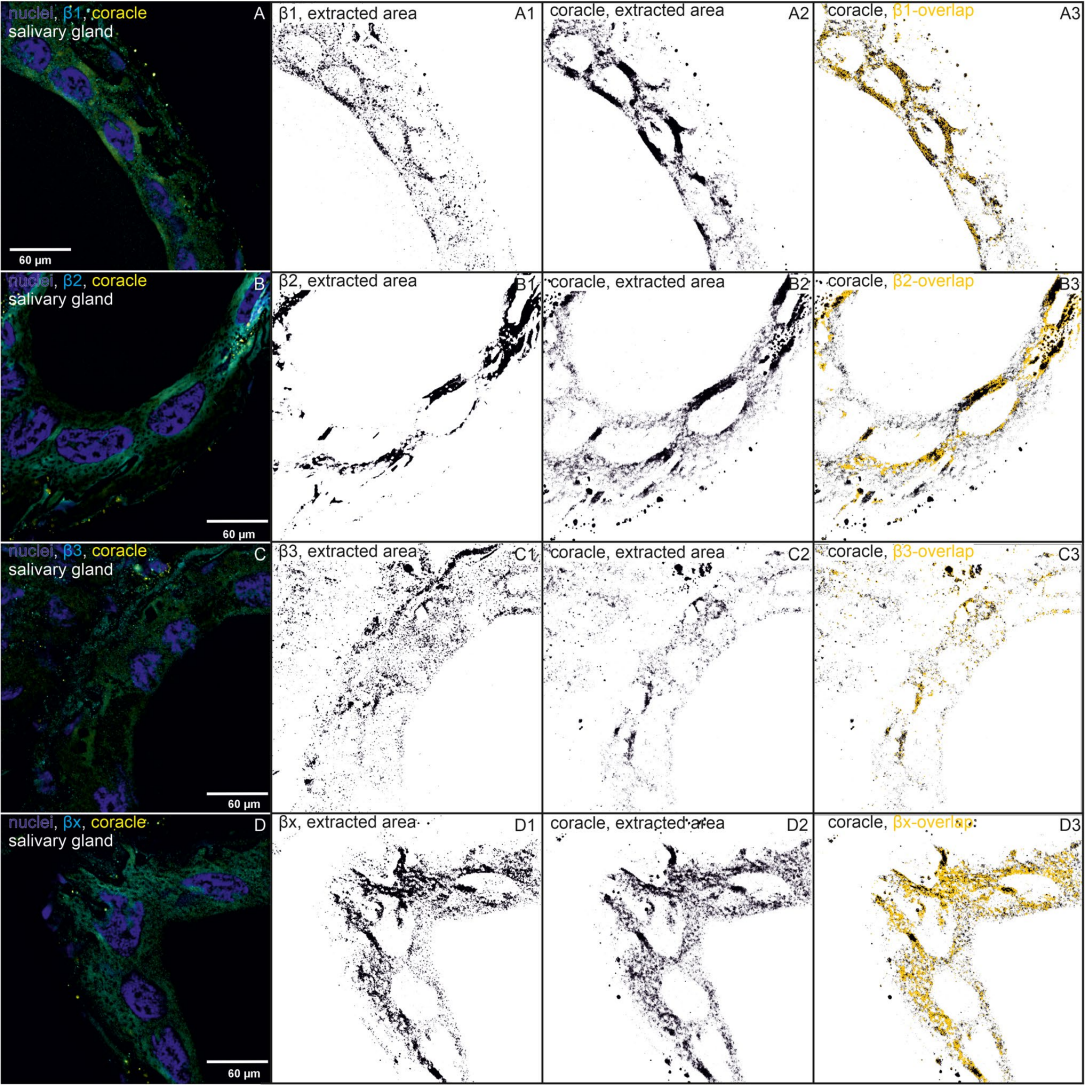
Immunohistochemistry images of salivary glands showing immunostainings of β-subunits, one SJ protein, and colocalizations. One reference image was chosen out of 3–4 similar biological replicates. The following binary images in one row show calculated and extracted areas by Fiji (black on white background, ImageJ version 1.54f). **a** Immunostainings of β-subunits that colocalize with discs large and their extracted areas and overlaps are shown. (A) Immunostainings with a subtracted background of nuclei (blue), β1 (cyan), and discs large (dlg, yellow) are shown in one biological replicate (out of three). (A1) The extracted area of β1 is shown. (A2) The extracted area of dlg is shown. (A3) The overlapping areas of β1 and dlg (yellow) are shown based on the binary image of dlg. (B–B3) The same for β2 (one out of four biological replicates), (C–C3) the same for β3 (one out of 4), (D–D3) the same for βx (one out of four biological replicates). **b** Immunostainings of β-subunits that colocalize with coracle and their extracted areas and overlaps are shown. (A) Immunostainings with subtracted background of nuclei (blue), β1 (cyan), and coracle (cora, yellow) are shown in one biological replicate (out of four). (A1) The extracted area of β1 is shown. (A2) The extracted area of the coracle is shown. (A3) The overlapping areas of β1 and coracle (yellow) are shown based on the binary image of coracle. (B–B3) show the same for β2 (one out of four biological replicates), (C–C3) show the same for β3 (one out of four biological replicates), (D–D3) show the same for βx (one out of four biological replicates)

**Fig. 2:**
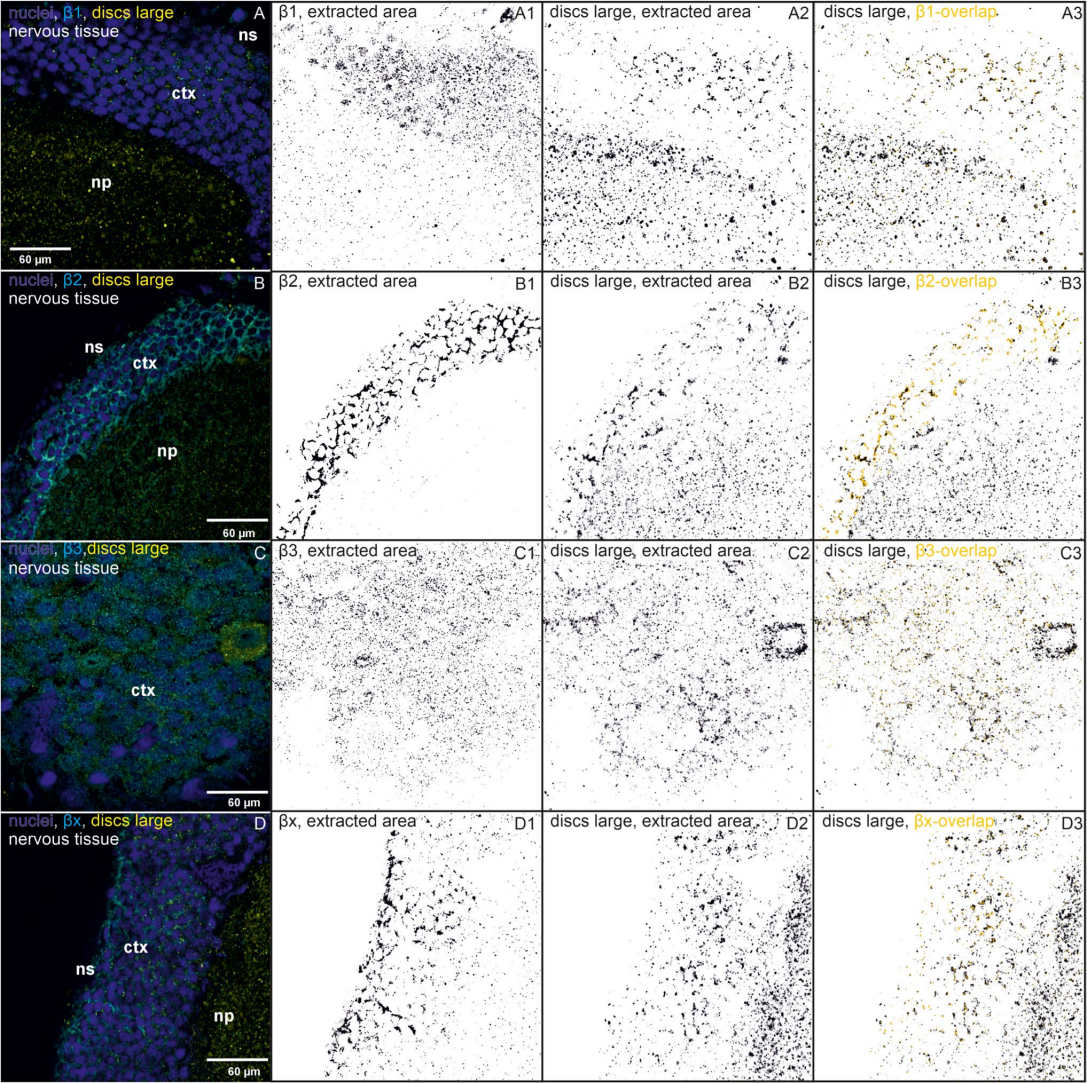

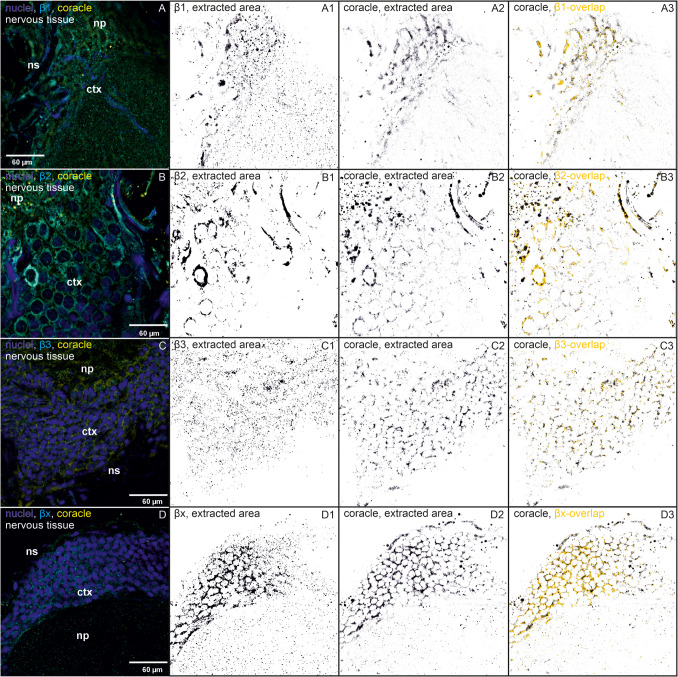
Immunohistochemistry images of nervous tissues show immunostainings of β-subunits, SJ proteins, nuclei, and colocalizations. One reference image was chosen out of 3–4 similar biological replicates. The following binary images in one row show calculated and extracted areas by Fiji (black on white background, ImageJ version 1.54f). **a** Immunostainings of β-subunits that colocalize with discs large and their extracted areas and overlaps are shown. (A) Immunostainings with a subtracted background of nuclei (blue), β1 (cyan), and discs large (dlg, yellow) are shown in one biological replicate (out of four). (A1) The extracted area of β1 is shown. (A2) The extracted area of dlg is shown. (A3) The overlapping areas of β1 and dlg (yellow) are shown based on the binary image of dlg. (B–B3) The same for β2 (one out of four biological replicates), (C–C3) the same for β3 (one out of three), (D–D3) the same for βx (one out of five biological replicates). **b** Immunostainings of β-subunits that colocalize with coracle and their extracted areas and overlaps are shown. (A) Immunostainings with subtracted background of nuclei (blue), β1 (cyan), and coracle (cora, yellow) are shown in one biological replicate (out of three). (A1) The extracted area of β1 is shown. (A2) The extracted area of the coracle is shown. (A3) The overlapping areas of β1 and coracle (yellow) are shown based on the binary image of coracle. (B–B3) show the same for β2 (one out of three biological replicates), (C–C3) show the same for β3 (one out of four biological replicates), (D–D3) show the same for βx (one out of four biological replicates)

**Fig. 3:**
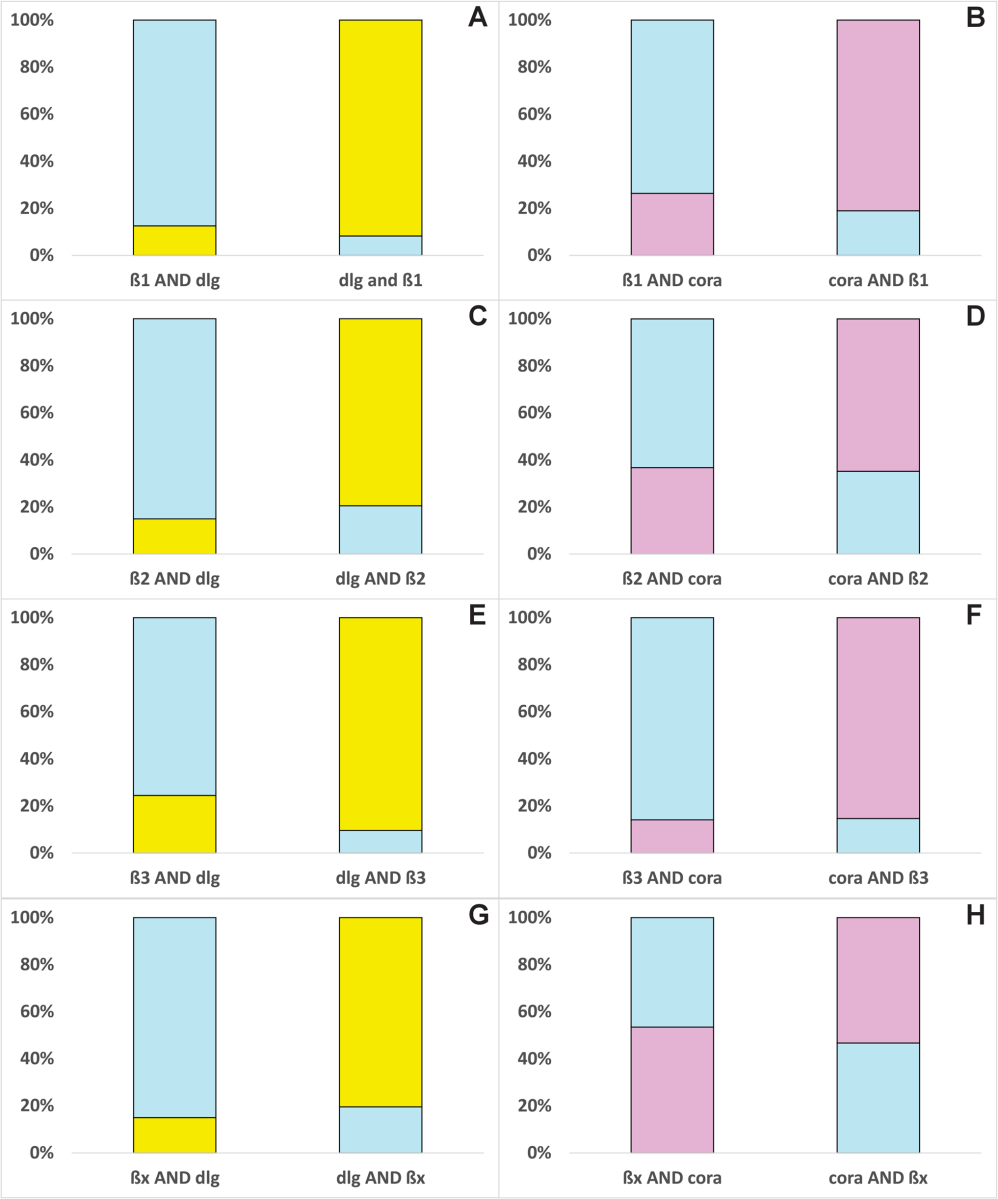
Percentage of the mean area of one β-subunit (light blue) colocalized with dlg (yellow) and coracle (cora, pink) in salivary glands and vice versa was calculated and shown in the bar chart. Total areas of the target proteins were extracted from IHC images, and overlapping areas were calculated and analyzed with Fiji (ImageJ, version 1.54f). The area of one protein overlapped by another is always 100%. **A** β1 overlapped by dlg (*N* = 3) and vice versa, **B** β1 overlapped by coracle (*N* = 4) and vice versa, **C** β2 overlapped by dlg (*N* = 4) and vice versa, **D** β2 overlapped by coracle (*N* = 4), **E** β3 overlapped by dlg (*N* = 4) and vice versa, **F** β3 overlapped by coracle (*N* = 4) and vice versa, **G** βx overlapped by dlg (*N* = 4) and vice versa, **H** βx overlapped by coracle (*N* = 4) and vice versa

**Fig. 4:**
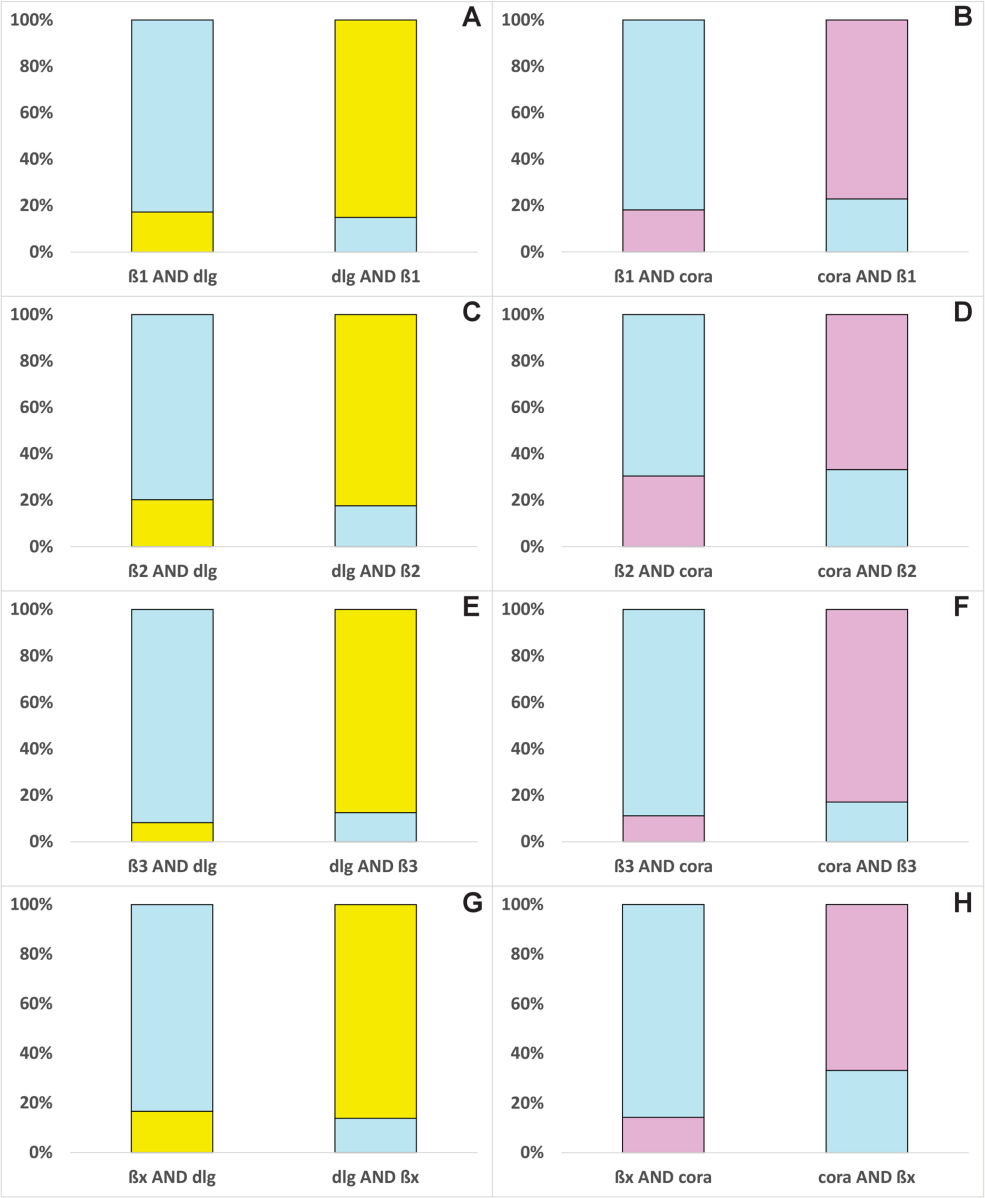
Percentage of the mean area of one β-subunit (light blue) colocalized with dlg (yellow) and coracle (cora, pink) in nervous tissue and vice versa was calculated and shown in the bar chart. Total areas of the target proteins were extracted from IHC images, and overlapping areas were calculated and analyzed with Fiji (ImageJ, version 1.54f). The area of one protein overlapped by another is always 100%. **A** β1 overlapped by dlg (*N* = 4) and vice versa, **B** β1 overlapped by coracle (*N* = 3) and vice versa, **C** β2 overlapped by dlg (*N* = 4) and vice versa, **D** β2 overlapped by coracle (*N* = 3), **E** β3 overlapped by dlg (*N* = 3) and vice versa, **F** β3 overlapped by coracle (*N* = 4) and vice versa, **G** βx overlapped by dlg (*N* = 5) and vice versa, **H** βx overlapped by coracle (*N* = 4) and vice versa

Our data show that all four β-subunits and the SJ proteins dlg and coracle colocalize. The NKA subunits β1, β2, and β3 clearly colocalize with both SJ proteins at the apical lateral regions of the cell membranes of the salivary glands where the SJs are localized. However, not all colocalizations between the proteins are exclusively located in this region (Figs. 1, 2, 3, and 4).

Salivary glands are formed by tall columnar cells, allowing for more detailed resolution and visualization of SJs. In this tissue, we found that dlg is clearly localized to the apical lateral region of the cell membranes and only to a minor extent to other regions. Coracle not only appears to be present throughout the cells but also accumulates at the apical region of the cell membranes (Fig. 1).

**Fig. 5:**
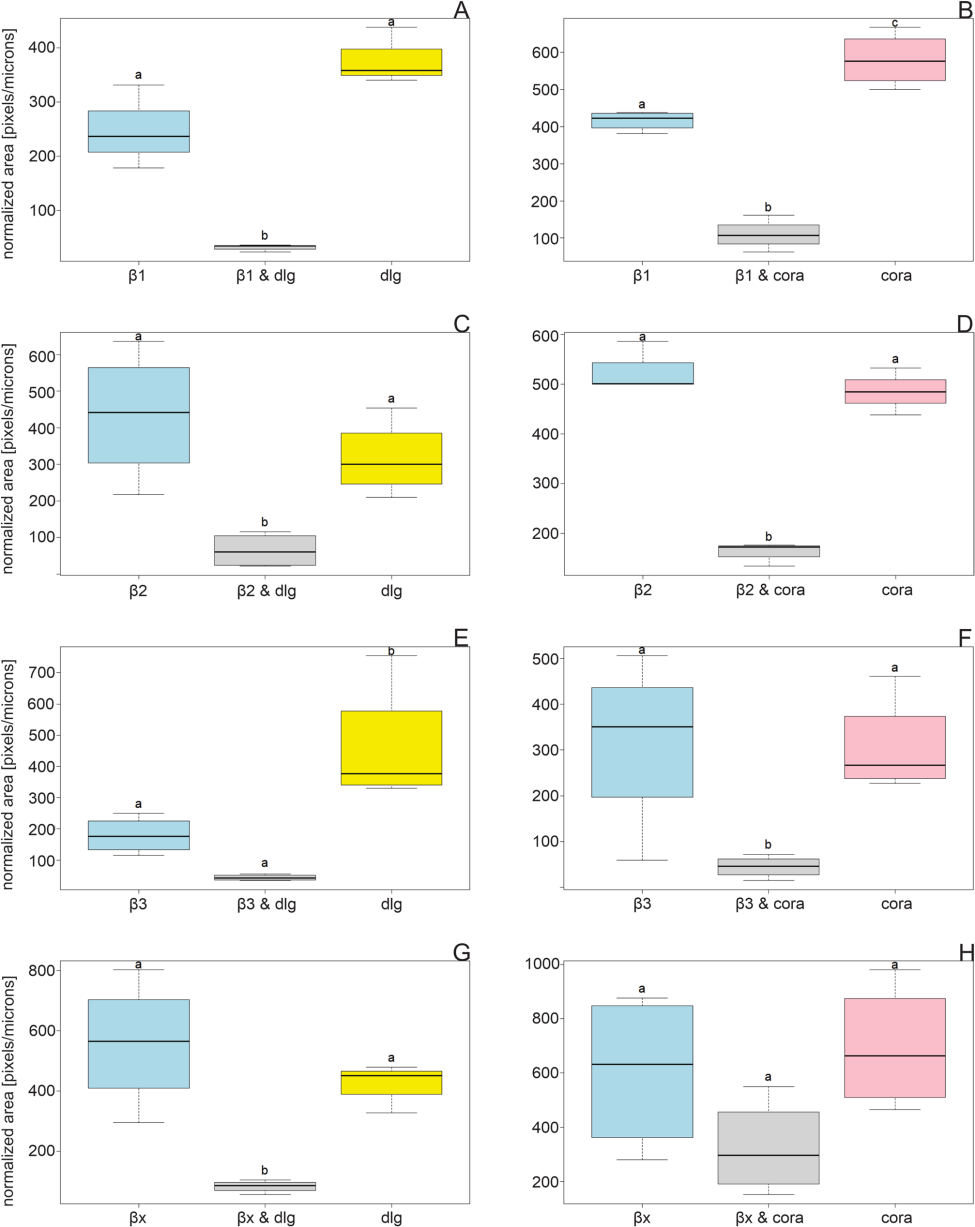
Normalized areas of β-subunits (light blue), colocalizations (grey), dlg (yellow), and coracle (cora, pink) from the salivary gland are shown (mean ± standard error). Areas were measured from IHC images and analyzed using Fiji (ImageJ, version 1.54f). ANOVA followed by TukeyHSD was performed using R version 4.4.2. Different letters above the boxplots represent significant differences between normalized areas. **A** β1 and dlg (*N* = 3), **B** β1 and coracle (*N* = 4), **C** β2 and dlg (*N* = 4), **D** β2 and coracle (*N* = 4), **E** β3 and dlg (*N* = 4), **F** β3 and coracle (*N* = 4), **G** βx and dlg (*N* = 4), **H** βx and coracle (*N* = 4)

β1 colocalizes with dlg, and their joint occurrence is almost equally distributed at both cell poles of the salivary gland cells (Fig. 1a (A–A3)). The areas of β1 and dlg are not different in size, but the colocalization area is significantly smaller, occupying only about 15% of the total area of each colocalization partner (Figs. 3A and 5A, Table S1). β1 colocalizes with coracle mainly in the apical lateral region of the cells where the SJs are located (Fig. 1b (A–A3)). The areas of β1, coracle, and their colocalization are significantly different. Coracle occupies a significantly larger area than β1, but their colocalization area is significantly smaller, occupying approximately 20 and 26% of the total area of coracle and β1, respectively (Figs. 3B and 5B, Table S1).

**Fig. 6:**
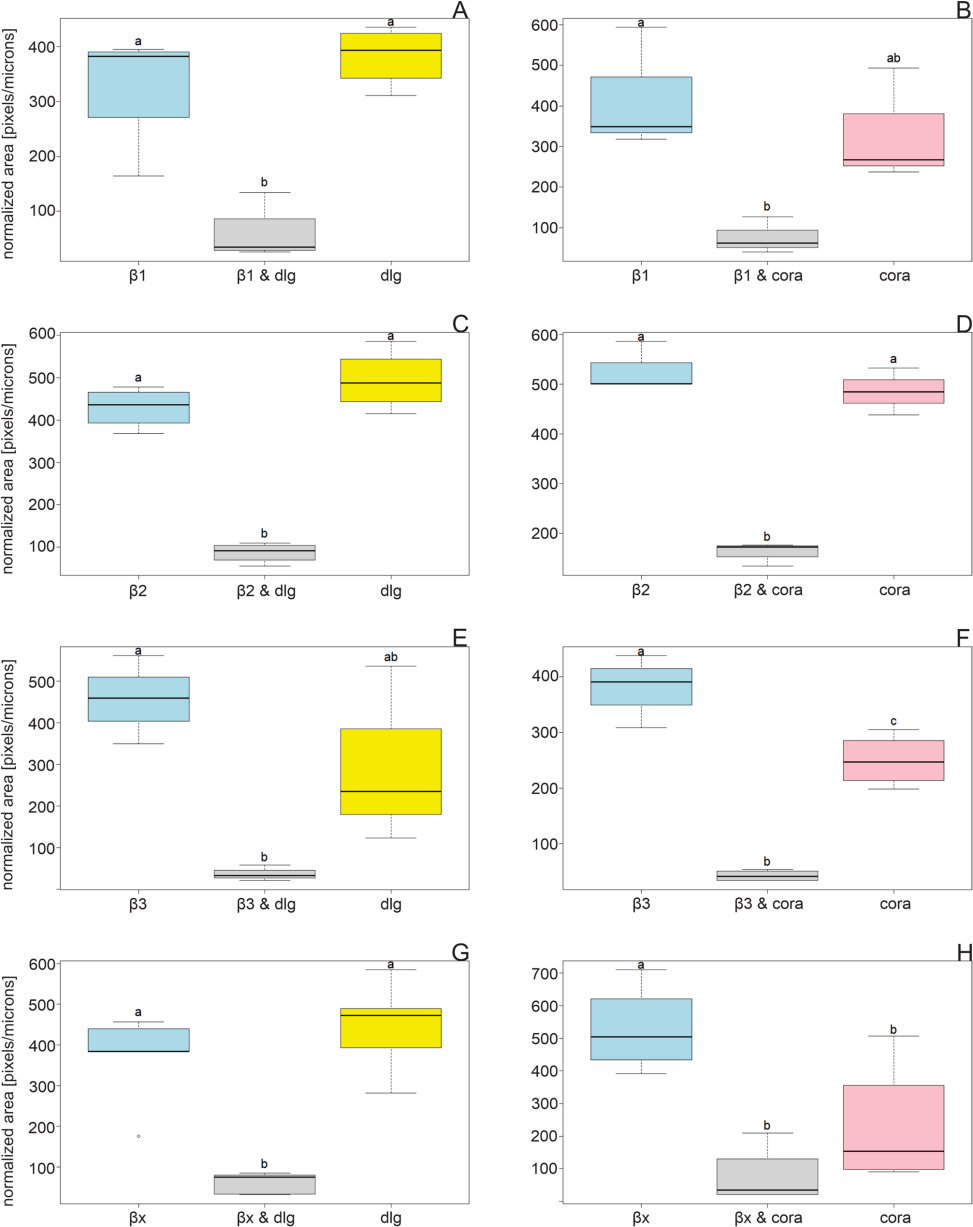
Normalized areas of β-subunits (light blue), colocalizations (grey), dlg (yellow), and coracle (cora, pink) from nervous tissues are shown (mean ± standard error). Areas were measured from IHC images and analyzed using Fiji (ImageJ, version 1.54f). ANOVA followed by TukeyHSD was performed using R version 4.4.2. Different letters above the boxplots represent significant differences between normalized areas. **A** β1 and dlg (*N* = 4), **B** β1 and coracle (*N* = 3), **C** β2 and dlg (*N* = 4), **D** β2 and coracle (*N* = 3), **E** β3 and dlg (*N* = 3), **F** β3 and coracle (*N* = 4), **G** βx and dlg (*N* = 5), **H** βx and coracle (*N* = 4)

The significantly smaller areas of the colocalizations compared to the areas of the corresponding proteins is a pattern that runs through the entire dataset of salivary glands and nervous tissue. This indicates that the SJ proteins are not just associated with one specific β-subunit, but with different β-subunits to different extents at the same time. In addition, β-subunits are required for the ion pump function of the NKA and are therefore widely distributed in cell membranes throughout the tissues and not only located at the apical lateral region of an epithelial cell. The data suggest that only a small fraction of the different β-subunits colocalize with dlg or coracle and vice versa (Figs. 1, 2, 3, 4, 5, and 6). However, in most cases, the areas of β-subunits and the SJ proteins do not differ significantly (Figs. 5 and 6, Table S1).

β2 colocalizes with dlg in apical lateral cell regions and lateral regions closer to the basal region of the gland cells. Remarkably, β2 does not overlap in the densest region of dlg appearance, but slightly above it. This suggests a marginally different location at the SJs (Fig. 1a (B–B3)) and implies an involvement in the SJs. β2 colocalizes with coracle not only in the apical cell region but also in other cell regions of the salivary glands to a similar extent (Fig. 1b (B–B3)). The colocalization area occupies more than 35% of the total area of each colocalization partner, which is larger than the colocalization area of β2 and dlg (Fig. 3C, D). This high percentage of colocalization indicates an association and could be indicative of common functions of β2 and coracle.

Dlg and β3 colocalize in apical lateral regions of the salivary gland cells but only to a low extent (Figs. 1a (C–C3) and 3E). Only fractions of β3 seem to colocalize with the SJ protein, which suggests that β3 mainly fulfills NKA pump-dependent functions. Coracle and β3 colocalize only to a low percentage, and the colocalization areas are distributed throughout the cell regions (Figs. 1b (C–C3) and 3F), which does not suggest a strong association with coracle.

βx and dlg do not colocalize at the apical lateral regions, and their overlaps appear to be small and scattered all over the gland cell, ruling out an SJ involvement for βx (Figs. 1a (D–D3) and 3G). In the salivary glands, areas of βx, coracle, and their colocalization do not differ significantly thus hinting to their co-occurrence (Fig. 5H, Table S1). The colocalized βx and coracle are found in almost all cell regions except the SJ region (Fig. 1b (D–D3)). The large colocalization area, occupying approximately 50% of the total area of βx and coracle, clearly suggests a common function that does not include the formation or maintenance of SJs (Fig. 3H). The analysis of the salivary glands suggests no involvement of βx in SJs, but an involvement of β1 and β2 is well supported by the data. It is also possible that β3 contributes to SJs, but as the data show, only to a minor extent.

We also analyzed the nervous tissue for colocalization between the β-subunits and the two SJ proteins. Because the cells are so small, no definitive statement can be made about SJ involvement. Both SJ proteins, dlg and coracle, can be found in the cell membranes. The strength of the signal after excitation depended entirely on the focal plane and the section of the cell, which indicate a specific location of the two SJ proteins.

All four β-subunits colocalize with both SJ proteins almost exclusively in the cortex of the nervous tissue. The distribution of dlg and coracle differed in that dlg also has a pronounced presence in the neuropil.

The distribution of β1 and dlg is different: β1 seems to be present in the nuclei, whereas dlg is mainly present in the membrane of the cell (Fig. 2a (A–A3)). However, they colocalize to a moderate extent (Fig. 4A). β1 and coracle colocalize to a higher extent in the cell membranes, which might indicate a functional connection between both proteins (Figs. 2b (A–A3) and 4B).

β2 and dlg are found in the apical cell membranes (Fig. 2a (B–B3)). They show the strongest colocalizations in the nervous tissue compared to the other β/dlg colocalizations, approximately 20% of the total area of β2, and dlg is occupied by the overlap of the other colocalization partner (Fig. 4C). Because dlg is almost exclusively present in apical lateral cell regions in the salivary glands (Fig. 1a), we assume that this is similar in the cortex of the nervous tissue. Therefore, the data suggest an involvement of β2 in SJs (Figs. 2a (B–B3) and 4C). β2 and coracle show the overall highest colocalization in the lateral cell membranes of the nervous tissue, which indicates a joint function of both proteins (Figs. 2b (B–B3) and 4D).

Similar to the colocalization patterns in the salivary glands, β3 colocalizes with dlg and coracle to a low extent and not as concentrated at one region, for example, β2 (Figs. 2a (C–C3), b (C–C3) and 4E, F), which argues against a strong association with the SJ proteins.

βx is mainly present in the vicinity of the neural sheath, which is the outer layer of the nervous tissue, whereas dlg can be found throughout the cortex in the cell membranes and in the neuropil. Despite these different distributions, they nevertheless show colocalization in the cell membranes (Fig. 2a (D–D3)). Although βx and dlg colocalize to a low extent, a connection between dlg and βx in the nervous tissue is still supported by our data (Fig. 4G). βx and coracle show a very strong colocalization in the cell membranes of the cortex, indicating an association and common functions of both proteins (Figs. 2b (D–D3) and 4H).

Data obtained in a previous round of experiments with a first version of the experimental protocol (for details, see the material and methods paragraph in the supplements) support the findings obtained here with the optimized protocol. The colocalization area of β1 and β2 with coracle in the nervous tissue almost reached 50% of the total protein (β-subunit, coracle, respectively), which was interpreted as a strong sign for an association (Fig. S5 Q & R, Fig. S6 E & F).

Besides the nervous tissue, in the first round of experiments, Malpighian tubules, ovaries, trachea, and muscles were analyzed via IHC (Fig. S6). However, the recorded images are not sufficient for detailed analysis because of lower magnification and only provide a first indication of possible β/SJ protein colocalizations. These data show that β1 colocalizes with coracle in ovaries, trachea, and nervous tissue. β2 colocalizes with coracle in the trachea, muscles, and nervous tissue, and β3 colocalizes with coracle in the Malpighian tubules (Fig. S5 & S6).

#### LC–MS/MS analyses

LC–MS/MS analyses of immunoprecipitation (IP) eluates of nervous tissue samples with magnetic beads cross-linked with C566.9 anti-coracle antibody could partly support an interaction of coracle and NKA subunits (see material & method section for the IP procedure in the supplements). High peptide spectrum matches (PSMs) indicated a high abundance (Madsen et al. 2015) of the SJ proteins neurexin and the NKA subunits β2, β3, α1B, and α1C (Fig. S7 & Table S2), whereas coracle itself was only recognized by a single typical PSM based on the *Drosophila* coracle sequence. The detected NKA subunits are highly abundant in the nervous tissue (Herbertz et al. 2022) and easily detectable by LC–MS/MS. For β2, the LC–MS/MS data supported the colocalization with coracle as observed in IHC. βx, in contrast, could not be detected by LC–MS/MS of coracle-baited IP despite their strong colocalization in the IHC analyses. We already experienced this lack of detection of βx in LC–MS/MS in a former study and could not find a satisfying explanation for this (Herbertz et al. 2022). The re-analyzed nervous tissue samples obtained from IP with anti-β1, β2, β3, and βx antibodies did not reveal any SJ proteins (for more details, see Herbertz et al. 2022, Table S2—S4), which can be explained by the high abundance of NKA subunits that preferably bind to the β-specific antibodies and β-subunits themselves.

## Discussion

In this study, we focused on the pump-independent functions of the NKA β-subunits, particularly with respect to their involvement in SJs. Immunohistochemistry and fluorescence confocal microscopy with the highest magnification possible on our device provided a good overview of the relationship between the β-subunits and SJs in the salivary glands and nervous tissue of *O. fasciatus*. Our data show that all four β-subunits colocalize with the SJ proteins dlg and coracle to varying degrees and in a tissue-specific manner. An exceptionally high colocalization of β2 and βx with coracle was detected in salivary glands and nervous tissue compared to the other β/coracle colocalizations. LC–MS/MS data also support an association of β2 with coracle. It also shows an association of β3 with coracle, which was not as strongly reflected in our IHC data. Although this may in itself not be sufficient to support an involvement in SJ formation or maintenance, an inspection of the proteins’ colocalization at specific cell regions in the IHC images allows a better assessment of the relationship between SJs and β-subunits. As dlg was shown to be almost exclusively present in the apical lateral cell regions where SJs are located, an involvement of the β-subunits in the SJs can be better corroborated.

Although a few studies support that the NKA is important for the establishment and maintenance of SJs, the biochemical role of the NKA in the formation of SJs is still not completely understood. It has been hypothesized that a low intracellular Na^+^ level preserved by the NKA is a necessary requirement for the formation of SJs (Paul et al. 2003), similar to the conditions needed for TJ formation in vertebrates (Rajasekaran et al. 2001). However, genetic mutations that hinder the catalytic function of the α1 subunit did not affect SJ formation in *Drosophila*, leaving open whether Na^+^ homeostasis has the same importance in invertebrates (Paul et al. 2007).

As Rajasekaran et al. (2001) show, the inhibition of the NKA by cardiac glycosides leads to disrupted junctions in canine kidney cells. Assuming that this also holds true for SJs, the uptake of cardiac glycosides by insects feeding on cardiac glycoside-containing host plants would need special adaptations for proper cell–cell contact formation. In this context, the evolution of cardiac glycoside resistant α1 paralogs as observed in *O. fasciatus* could well be of importance. Only a few invertebrates that are adapted to cardiac glycosides are known to possess duplicated α1 paralogs that differ in resistance-conferring amino acid substitutions and consequently also in activity and resistance levels (Dalla and Dobler 2016; Dalla et al. 2017). In *O. fasciatus*, functional units of the NKA can be combinations of one out of four α1 and one out of four β-subunits (Dalla et al. 2017; Herbertz et al. 2024). Since resistance to cardiac glycosides and differing activities should influence Na^+^ balance and could thus have an effect on SJ functionality—judging from the situation in vertebrates (Rajasekaran et al. 2001)—the investigation of the interplay between NKA isoforms and functional SJs in *O. fasciatus* remains highly fascinating. So far, knowledge about pump-independent functions of the NKA, such as SJ formation (Genova and Fehon 2003; Oshima and Fehon 2011; Paul et al. 2003, 2007) and tracheal tube size control (Paul et al. 2003, 2007), was restricted to research in *Drosophila*.

### Analyses of β and SJ protein colocalization via IHC

Our IHC image analyses show that all four β-subunits colocalize with the SJ proteins dlg and coracle to varying degrees and in a tissue-specific pattern. Previously, we found that the knockdown of β1, β2, and β3 led to severe problems during the final ecdysis in *O. fasciatus*. We hypothesized that this might be caused by disrupted SJ formation (Herbertz et al. 2023). Right before ecdysis, the cell number increases, cells get repositioned (Chapman 1970), and cell–cell contacts form. In this stage, the disruption of SJs could lead to malformation of the cuticle as observed in *Drosophila* embryos (Lamb et al. 1998). Such malformations could be one reason for a problematic molting. Furthermore, a link between disrupted SJs and altered tracheal morphology was drawn (Herbertz et al. 2023; Paul et al. 2003, 2007). During the preparatory phase of ecdysis, air-swallowing is an important strategy to split the old cuticle (Wadsworth et al. 2014). Enlarged tracheal tubes might hinder a sufficient airflow and the building up of sufficient internal pressure to get rid of the old exoskeleton.

Here, we have shown a colocalization of β2 and β3 with dlg in the salivary glands, which supports the involvement of β-subunits in SJs. Dlg can be clearly assigned to the SJs; it is a SJ resident protein (Rice et al. 2021). The anti-dlg antibody beautifully stained the SJ region in between the large salivary gland cells in *O. fasciatus*. The SJs could thus be clearly distinguished from the rest of the cell regions. This was different for the anti-coracle antibody. Coracle also strongly accumulated at the apical lateral region but not exclusively. Rather, we found coracle throughout the cells, which makes an exclusive SJ assignment difficult. Due to the use of the same primary antibody host, it was not possible to immunostain both, dlg and coracle, at the same time to additionally verify the apical lateral position of coracle in the SJs.

Coracle is a core SJ protein, which is located in the SJs. It is important for the formation and maintenance of the junctions (Rice et al. 2021). Its more widespread localization in cells of *O. fasciatus* might be explained by further functions, which might not all be known yet. In *Drosophila*, coracle fulfills functions besides its paracellular barrier function. Recent studies of SJ proteins in *Drosophila* suggest that coracle is involved in follicle epithelium development, signaling pathways that control proliferation (Holtwick et al. 2024), and larval developmental processes prior to SJ formation, including dorsal closure, head involution, regulation of dorsal vascular structure, and function (Rice et al. 2021). In adult *Drosophila* flies, coracle also has essential functions in the development of eyes, wings, ocelli, and other tissues (Lamb et al. 1998)*.* In *O. fasciatus*, β2 and βx both colocalize strongly with coracle in the salivary glands and the nervous tissue. These colocalizations are not restricted to the apical lateral SJs, but are present throughout the cell, suggesting an additional common function that we do not yet know about.

Nervous tissue cells are very small, so the data here must be interpreted with more caution. Colocalizations are visible, but the exact delineation of SJ regions is very difficult. According to our results, dlg is SJ specific as also previously described (Rice et al. 2021), which allows us to make assumptions about the SJ affiliations of β-subunits, even in the nervous tissue. The nervous tissue is protected by a hemolymph-brain barrier to prevent uncontrolled passage of solutes, metabolites, or other xenobiotics. This barrier is formed by two specific classes of glial cells, the perineurial glial cells, which form the outer layer and act as a first diffusion barrier, and the subperineurial glial cells, which form the SJs and block paracellular diffusion and separate the nervous system from the hemolymph (Limmer et al. 2014).

Overall, the colocalization areas of β-subunits and coracle were larger than those of β-subunits and dlg. Dlg is also present in the neuropil, but only little β-overlaps were highlighted in this area. In *Drosophila* brains, dlg is important for short-term memory and synaptic plasticity (Bertin et al. 2022). Therefore, the presence of dlg in the neuropil of *O. fasciatus* is not surprising, because it is a region dense with synapses. Both SJ proteins are present throughout the cortex, in contrast to *Drosophila* where SJ localization is restricted to the hemolymph-brain barrier (Limmer et al. 2014). This is different in *O. fasciatus*, where we found both SJ proteins in the membranes of the glial cells in direct contact with neighboring glial cells throughout the cortex. So far, nothing is known about the specific structure of the hemolymph-brain barrier or additional protective layers in the nervous tissue in *O. fasciatus*. The cardiac glycoside-adapted large milkweed bug may need a stronger protective layer in the periphery of the nervous tissue than a non-adapted fruit fly.

β1, β2, and βx are located in the cell membranes in close contact to neighboring cells together with dlg, which suggests a different extent of involvement of the three β-subunits in SJs. β2 and dlg show the strongest area overlaps here. In an ongoing study (unpublished data, Herbertz et al.), we found that β2 knockdown increases the concentration of the cardenolide frugoside in the nervous tissue of *O. fasciatus* compared to the control, while β1 knockdown increases the concentration of the more polar cardenolide ouabain, supporting the hypothesis that β1 and β2 are part of the SJs. According to our previous analyses, β2 is a major component of the NKA in the nervous tissue with a high prevalence (Herbertz et al. 2022). Possibly, β2 fulfills a broad range of functions in this tissue including SJ formation.

βx and dlg did not colocalize in the salivary glands but did so in the nervous tissue, yet their specific task in this tissue remains open for now. According to our data, βx and coracle appear to have a strong connection and seem to fulfill SJ-independent functions together, which need further investigation. The enigmatic βx has no homologs in other insects and differs from the other NKA β-subunits in *O. fasciatus* by its truncated N-terminus (Herbertz et al. 2022)*.* Previous knockdown studies indicated no effect of βx on ecdysis success in contrast to the other β-subunits (Herbertz et al. 2023), thus further suggesting that βx is not involved in SJ formation but fulfills different functions than the other NKA β-subunits.

### Additional analyses of different tissues

The first round of experiments included additional tissues imaged at a lower magnification (Fig. S5 & S6). These experiments support that β1 colocalizes with coracle in the ovaries of *O. fasciatus* (Fig. S5 I) where a high abundance of β1 was observed previously (Herbertz et al. 2023). In a recent study, Holtwick et al. (2024) found coracle to be present in the lateral membranes of follicle cells and particularly abundant in follicle stalk cells in *Drosophila* and hypothesized that coracle fulfills a SJ-independent role in follicle epithelium development (Holtwick et al. 2024). Our data supports the described localization of coracle (Fig. S6 B—B2).

Further, we found that β1 and β2 colocalize with coracle in the tracheal epithelia (Fig. S5 M & N, Fig. S6 C—C1 & D—D1). Here, not all background noise was removable without eliminating important information. Furthermore, it seems as if the anti-β2 antibody somehow stains the ridges of the taenidiae, possibly due to the high abundance of β2 in the tracheal epithelia and the tracheal surface structure. Nevertheless, these data are consistent with previous studies in which the *Drosophila* homolog of β2, Nrv2, is characterized as a major part of the SJ core complex (Genova and Fehon 2003). We can only hypothesize on the basis of the given data that at least β2 and coracle fulfill a common function, which is most likely the formation of SJs. Although the colocalization of β1 and coracle reached 50%, our arbitrarily chosen threshold for a strong overlap (for details, see supplemental material & methods); the abundance of the β-subunit seems negligible in the tracheal epithelia.

In the muscle tissue, β2 is mainly present in the sarcolemma (Herbertz et al. 2023), the membrane of the muscle cells (Fig. S5 V & S6 G—G1). Therefore, it makes sense that most colocalizations with coracle were detected in this region and less between the myofibrils of the muscles. Because no SJ is present in muscle tissue, this colocalization indicates a different common function.

In addition, the data showed that β3 colocalized with coracle in the Malpighian tubules (Fig. S5 G & S6 A—A1). It is impossible to judge whether they form SJs there, nevertheless, we are able to show that β3 and coracle are present and colocalize. Although the presence of Nrv3, the *Drosophila* homolog of β3, was restricted to the nervous tissue (Baumann et al. 2010) and was found to lack junctional activity (Paul et al. 2007), our data do not confirm these findings for *O. fasciatus*. Even though Nrv3 and β3 are homologs, their location and functions may slightly differ between organisms.

We found no strong interdependence between β-subunits and coracle in the midgut, even though colocalizations between the proteins are supported by our data (Fig. S5 A – D). Further corroboration by the available LC–MS/MS methods was impossible as the NKA is not sufficiently abundant in tissues other than the nervous tissue.

### LC–MS/MS analyses of nervous tissue samples

In contrast to the IHC results discussed above, the LC–MS/MS analyses allow the discrimination between α1 paralogs. Immunoprecipitation with beads cross-linked to anti-coracle antibodies revealed an association of α1B and α1C as well as β2 and β3 to coracle. Only one PSM was found for coracle, which is not surprising, as the UniProt database search was based on the *Drosophila* coracle sequence in this case. This sequence may be highly divergent from *O. fasciatus* in certain regions, as is also the case in other insect species (for details, see materials and methods section and the coracle alignment Fig. S3 in the supplements).

LC–MS/MS analyses of nervous tissue samples of IP with anti-β1, β2, and β3 antibodies cross-linked to magnetic beads could not reveal any coimmunoprecipitated SJ proteins. Low PSMs or missing SJ proteins in the precipitates might be due to the limited binding capacities of the antibodies coupled to the magnetic beads. Mainly NKA subunits were coimmunoprecipitated with the anti-β-subunit-coupled beads (for details, see Herbertz et al. 2022 & Table S3), as well as other abundant proteins (spectrin, actin, tubulin, myosin, etc.). These abundant proteins might have masked other less abundant proteins, such as members of the SJ core complex or SJ resident proteins. Lower order complexes cannot be resolved if there is an overabundance of other strong complex partners and if the binding capacity of the antibodies is fully utilized.

## Conclusion

Our study, together with previous studies from our working group (for details, see Herbertz et al. 2022, 2023), supports the interplay between NKA β-subunits and SJs in *O. fasciatus.* The use of two SJ protein-specific antibodies provided a reliable framework for the assessment of beta-subunit involvement in SJs. Some β-subunits seem to be localized at and as a part of SJs, while they also seem to perform pump and SJ-independent functions together with the SJ proteins. Future studies investigating more physiological aspects of SJs are highly recommended to further elucidate these possibilities.

Our results add further insights about the functional role of the four β-subunits in *O. fasciatus*. Besides having chaperone-like functions and thus being vital for the correct NKA assembly and membrane integration, β-subunits associate with SJ proteins. The colocalization of β-subunits with SJ proteins and the possible involvement in SJs is tissue-specific.

To qualitatively substantiate our findings and to learn about the distribution of toxic cardiac glycosides in insects with malfunctional NKAs and SJs, an ongoing study combines RNA interference experiments and HPLC analyses. This will shed light on the effect of cardiac glycoside exposure on milkweed bugs in which the β-subunits were individually knocked down and help us to expand our knowledge about the function of different subunits of the NKA in *O. fasciatus* further.

## Supplementary Information

Below is the link to the electronic supplementary material.

Supplementary Material 1 (DOCX 21.9 KB)

Supplementary Material 2 (PDF 15.7 MB)

Supplementary Material 3 (PDF 523 KB)

Supplementary Material 4 (PDF 302 KB)

Supplementary Material 5 (PDF 9.10 MB)

Supplementary Material 6 (PDF 6.98 MB)

Supplementary Material 7 (PDF 27.1 MB)

Supplementary Material 8 (PDF 2.26 MB)

Supplementary Material 9 (PDF 20.4 MB)

Supplementary Material 10 (XLSX  13.8 KB)

Supplementary Material 11 (XLSX  14.0 KB)

Supplementary Material 12 (XLSX  1.24 MB)

Supplementary Material 13 (XLSX 2.33 MB)

## Data Availability

All relevant data can be found within the article and its supplementary information.
