# Supplementary material for: The large milkweed bugs’ Na,K-ATPase β-subunits colocalize with septate junction proteins in a tissue-specific manner
Source: Cell Tissue Res. 2025 Mar 26;400(3):347–63. doi: 10.1007/s00441-025-03965-3 (PMC12125057; doi:10.1007/s00441-025-03965-3)
Supplement: Supplementary file 1 — Supplementary Material 1 (DOCX 21.9 KB) [file 441_2025_3965_MOESM1_ESM.docx]

**The large milkweed bugs' Na,K-ATPase β-subunits colocalize with septate junction proteins in a tissue-specific manner**

Marlena Herbertz^1*^, Christian Lohr^2^, Susanne Dobler^1^

^1^ Institute of Cell and Systems Biology of Animals, Molecular Evolutionary Biology, Universität Hamburg, 20146 Hamburg, Germany

^2^ Institute of Zell and Systems Biology of Animals, Neurophysiology, Universität Hamburg, 20146 Hamburg, Germany

*corresponding author: marlena.herbertz@uni-hamburg.de

Supplement

Material and Methods

Identifying coracle homologs

To check for conserved regions and sequence similarities in different insect species, including *O. fasciatus,* the coracle protein sequences of different insects were aligned with the coracle protein sequences of *D. melanogaster* (Diptera, Drosophilidae, UniProt accession numbers: Q9V8R9, A0A0B4LFX4, A0A0B4LG23). The species included *Pararge aegeria* (Lepidoptera, Nymphalidae, UniProt accession numbers: S4NVH6, S4P6G9, S4PZT5), *Operophtera brumata* (Lepidoptera, Geometridae, UniProt accession numbers: A0A0L7KHN0, A0A0L7L3N5), *Danaus plexippus plexippus* (Lepidoptera, Nymphalidae, UniProt accession number: A0A212EJP2), *Corethrella appendiculata* (Diptera, Corethrellidae, UniProt accession number: W4VRQ9), *Cotesia congregata* (Hymenoptera, Braconidae, UniProt accession number: B1GS95), *Rhodnius prolixus* (Hemiptera, Reduviidae, UniProt accession number: G1K0N0) and *Oncopeltus fasciatus* (Hemiptera, Lygaeidae, NCBI TSA sequence ID: GCXY01047324.1). The multiple sequence alignment was performed with the online tool Clustal Omega provided by EMBL-EBI (see Fig. S3).

Tissue dissection (first IHC experiments with an anti-coracle antibody)

A total of 16 female large milkweed bugs were killed by freezing shortly at -80°C one to two days after final ecdysis. The dead animals were immediately incised between thorax and abdomen. Midgut, Malpighian tubules, and ovaries were dissected on ice with two ultra-fine forceps under chilled ultra-pure water containing a protease inhibitor cocktail (Roche, Basel, Switzerland). Head, thorax, and dissected tissues were fixed over night with 4% paraformaldehyde (Merck KGaA, Darmstadt, Germany) in phosphate buffered saline (PBS, pH 7.4).

Afterwards, the fixed tissues were washed in PBS for 5 minutes. The torso was mounted in 5% low melt agarose (AGS GmbH, Heidelberg, Germany) and sliced into 300 µm wide slices with a vibratome (VT1000S, Leica, Wetzlar, Germany) as described in Herbertz et al. (2022).

Immunohistochemistry

We followed the same protocol as described in the main text, except for the secondary anti-chicken antibody and the last embedding step. Here, we used a goat anti-chicken blue fluorescent secondary antibody (Alexa 488, 1:1000 in 1:2 diluted blocking solution; Sigma, St. Louis, Missouri, USA). For embedding, the torso and not sliced tissues were washed, and the slices and tissues were transferred to object slides, embedded with 30 µl 4’-6-Diamidine-2-phenylindole (DAPI) solution (Dianova, Hamburg, Germany) and Shandon Immu-Mount, and covered with cover slips. On the next day the cover slips were sealed with nail-polish.

Microscopy and image analyses were performed as described in the main text except that the area occupied by nuclei was included here and only strong background noise was excluded.

Data analysis

All measured areas of the target β-subunits, coracle, and nuclei, as well as colocalizations were normalized to the total area of one tissue (area/total area). Relative abundances of overlapping areas and the target area were expressed in percentage of the target area (analyzed colocalizations: nuclei area vs β overlap, β area vs nuclei overlap, β area vs coracle overlap, coracle area vs β overlap) (See Fig. S5).

Immunocytochemistry

*Sf*9 cells were infected with recombinant baculoviruses (P2 stock) containing an α and a β subunit (Herbertz et al., 2023a). 72 hours after infection 500 µl cell suspension (containing 2.5 *10^5^ cells/ml) were plated in 24-well culture plates on 11 mm glass coverslips and incubated for one hour at 27°C. We followed our previously published protocol with slight modifications (Dalla and Dobler, 2016). After fixation and permeabilization, the cells were blocked with 5% BSA in TBS-T for one hour at room temperature. For recognition of the β-subunits 2-3 µg per well of each of the four affinity purified, polyclonal β-specific primary antibodies (chicken anti-β1, rabbit anti-β2, chicken anti-β3, and rabbit anti-βx diluted in TBS with 1%/0,1% Tween BSA; customized by Davids Biotechnologie, Regensburg, Germany) were used and incubated over night at 4°C. Each β-subunit was incubated with all four antibodies separately to evaluate their specificity. Because the β2 specific antibody showed a different binding pattern, untransfected *Sf*9 cells and *Sf*9 cells transfected with an empty bacmid were used as an additional control for the anti- β2 antibody. After sufficient washing a two-hour incubation with fluorophore bound secondary antibodies followed (Alexa 488 anti-rabbit (β2 & βx) and Alexa 488 anti-chicken (β1 & β3) (Invitrogen, Carlsbad, California, USA) 1:1000 diluted in TBS with 1% BSA/0.1 % Tween). After three washing steps, the cells were mounted in mounting medium containing DAPI (ImmunoSelect, Dianova). The cells were imaged using a fluorescence microscope Olympus BX51 (Olympus, Hamburg, Germany) equipped with a Olympus DP71 camera and a 20x lens ([UPlan Fl N 20X Oil](https://www.microscope.healthcare.nikon.com/de_EU/selectors/objective-comparison/-1829), Olympus) (see Fig. S4). All four primary antibodies do not bind to a non-target β-subunit and only showed a clear signal when bound to their target β-subunit. The anti-β2 antibody also bound to an endogenous β-subunit in the *Sf*9 cells, which does not contradict the specificity of the antibody, but rather shows that β2 homologs are expressed at low levels in Sf9 cells (Fig. S4).

Immunoprecipitation with magnetic beads

Crosslinking of antibody

Thirty micrograms of monoclonal mouse anti-coracle primary antibody (C566.9; DSHB Hybridoma Bank) were coupled to magnetic beads (GE Healthcare, Chalfont Buckinghamshire, United Kingdom) according to the protocol by Herbertz et al. (2022). Successful coupling was evaluated by dot blotting the initial antibody solution and the antibody solution after incubation with magnetic beads (Herbertz et al. 2022). Because of insufficient binding the antibody solution was again incubated with the magnetic beads for 48 hours and successful coupling was verified via dot blot.

Protein extraction

Protein of 16 pooled nervous tissues of *O. fasciatus* was homogenized in 200 µl ice-cold RIPA buffer (50 mM Tris/HCl pH 8, 150 mM NaCl, 1% Triton-X-100, 0.5% sodium deoxycholate, 0.1% SDS (Carl Roth GmbH, Karlsruhe, Germany)) containing protease inhibitor cocktail with an all-glass grinder (Wheaton) and incubated on ice for 30 minutes. The protein solution was centrifuged for 5 minutes at 4 000 rpm at 4°C. 150 µl of the supernatant, together with 600 µl binding buffer (1.2 M KH2PO4, pH 7 (Carl Roth GmbH, Karlsruhe, Germany)) containing protease inhibitor, was incubated with the anti-coracle antibody-coupled magnetic beads with slow rotation at 4°C for 24 h. After three washing steps with binding buffer, proteins were eluted from the crosslinked antibodies by adding 50 µl 0.1 M Glycin (pH 2.5) (Carl Roth GmbH, Karlsruhe, Germany). This step was repeated and both eluates were neutralized by adding 6 µl of 1 M Tris. The protein eluates were subjected to LC-MS/MS analysis.

LC-MS/MS

Tryptic digestion and LC-MS/MS analysis followed the protocol described in Herbertz et al. (2022). The newly generated dataset originating from IP with anti-coracle antibody bound to magnetic beads was analyzed together with four datasets generated during a previous study, which were re-analyzed for this study (Herbertz et al., 2023b) with regard to proteins involved in cell-cell contact formation following the protocol of Herbertz et al. (2022). Peptide spectrum matches (PSMs) were used as an indicator for abundance – they are described as the total number of identified peptides counting also repeatedly identified ones (see Figure S7).
