# Supplementary material for: The large milkweed bugs’ Na,K-ATPase β-subunits colocalize with septate junction proteins in a tissue-specific manner
Source: Cell Tissue Res. 2025 Mar 26;400(3):347–63. doi: 10.1007/s00441-025-03965-3 (PMC12125057; doi:10.1007/s00441-025-03965-3)
Supplement: Supplementary file 2 — Supplementary Material 2 (PDF 15.7 MB) [file 441_2025_3965_MOESM2_ESM.pdf]

The large milkweed bugs' Na,K-ATPase β-subunits colocalize with septate junction proteins in a tissue-specific manner

Marlena Herbertz[1*], Christian Lohr[2], Susanne Dobler[1]

[1] Institute of Cell and Systems Biology of Animals, Molecular Evolutionary Biology, Universität Hamburg, 20146 Hamburg, Germany

[2] Institute of Zell and Systems Biology of Animals, Neurophysiology, Universität Hamburg, 20146 Hamburg, Germany

*corresponding author: marlena.herbertz@uni-hamburg.de

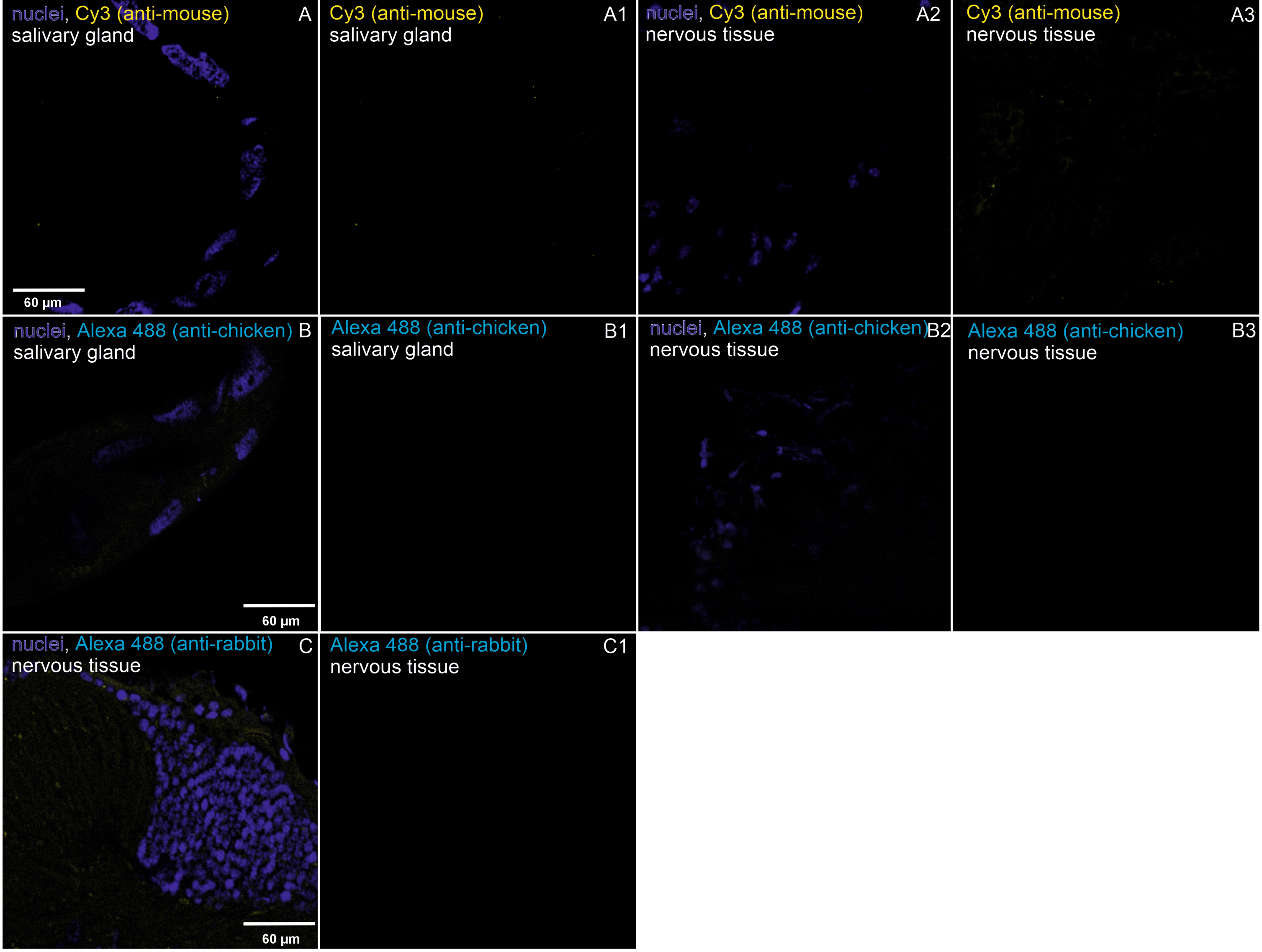

Figure S1: Immunohistochemical images of negative controls in salivary glands and nervous tissue from *Oncopeltus fasciatus*. As negative controls tissues stained with secondary antibody only were used. (A) A salivary gland stained with Dapi (blue) and Cy3 (anti-mouse secondary antibody, yellow) is shown. (A1) Only Cy3 immunostain is shown here, the signal with equal or higher detector gains than the ones used for the main samples was negligible. (A2) Nervous tissue stained with Dapi (blue) and Cy3 is shown. (A3) Only Cy3 immunostain is shown here, the signal was clearly weaker than signals of stainings in the main samples at same or higher detector gains. (B) A salivary gland stained with Dapi (blue) and Alexa 488 (anti-chicken secondary antibody, cyan) is shown. (B1) Only Alexa 488 immunostain is shown here, no signal was recorded. (B2) Nervous tissue stained with Dapi (blue) and Alexa 488 (anti-chicken) is shown. (B3) Only Alexa 488 immunostain is shown here, no signal was recorded after setting same or higher detector gains as for the main samples. (C) Nervous tissue stained with Dapi (blue) and Alexa 488 (anti-rabbit secondary antibody, cyan) is shown. (C1) Only Alexa 488 immunostain is shown here, no signal was recorded. (scale bars: 60µm)
