# Supplementary material for: The large milkweed bugs’ Na,K-ATPase β-subunits colocalize with septate junction proteins in a tissue-specific manner
Source: Cell Tissue Res. 2025 Mar 26;400(3):347–63. doi: 10.1007/s00441-025-03965-3 (PMC12125057; doi:10.1007/s00441-025-03965-3)
Supplement: Supplementary file 3 — Supplementary Material 3 (PDF 523 KB) [file 441_2025_3965_MOESM3_ESM.pdf]

The large milkweed bugs' Na,K-ATPase β-subunits colocalize with septate junction proteins in a tissue-specific manner

Marlena Herbertz[1*], Christian Lohr[2], Susanne Dobler[1]

[1] Institute of Cell and Systems Biology of Animals, Molecular Evolutionary Biology, Universität Hamburg, 20146 Hamburg, Germany

[2] Institute of Zell and Systems Biology of Animals, Neurophysiology, Universität Hamburg, 20146 Hamburg, Germany

*corresponding author: marlena.herbertz@uni-hamburg.de

The discs large protein sequence (UniProt: P31007 DLG1_DROME Disks large 1 tumor suppressor protein, *Drosophila melanogaster*) was used as a reference to search via tblastn for potential homologs in a transcriptome of *O. fasciatus* using the transcriptome shotgun assembly (TSA) method. Here are the results. The antibody epitope is highlighted in yellow.

An official website of the United States government

Here's how you know

The .gov means it’s official.
Federal government websites often end in .gov or .mil. Before sharing sensitive information, make sure you’re on a federal government site.

Feedback

The site is secure.
The **https://** ensures that you are connecting to the official website and that any information you provide is encrypted and transmitted securely.

Your search is limited to records that include: Oncopeltus fasciatus (Dallas, 1852) (taxid:7536)

**Job Title**	Protein Sequence ...
**RID**	S1K730F4016 Search expires on 01-11 14:31 pm
**Program**	TBLASTN
**Database**	tsa (2 databases)
**Query ID**	lcl|Query_2970441
**Description**	unnamed protein product ...
**Molecule type**	amino acid
**Query Length**	970

Descriptions

Description	Max Score	Total Score	Query Cover	E value	Per. Ident	Acc. Len	Accession
TSA: Oncopeltus fasciatus breed wildtype s6732_L_16687_0_59_0_FORK, transcribed RNA sequence	963	963	79%	0.0	64.37%	2401	GCXY01049860.1
TSA: Oncopeltus fasciatus breed wildtype s6733_L_16687_1_60_6_FORK, transcribed RNA sequence	729	729	48%	0.0	74.84%	1617	GCXY01049861.1
TSA: Oncopeltus fasciatus breed wildtype C252450_36_0, transcribed RNA sequence	173	173	48%	2e-41	27.29%	3507	GCXY01035507.1
TSA: Oncopeltus fasciatus breed wildtype C219282_8_0, transcribed RNA sequence	154	154	10%	5e-40	78.95%	592	GCXY01029008.1
TSA: Oncopeltus fasciatus breed wildtype C249004_13_0, transcribed RNA sequence	154	154	48%	2e-36	27.40%	1965	GCXY01034813.1
TSA: Oncopeltus fasciatus breed wildtype C252560_7_0, transcribed RNA sequence	149	149	49%	1e-33	25.40%	3658	GCXY01035534.1
TSA: Oncopeltus fasciatus breed wildtype s4502_L_10050_0_11_2_BUBBLE, transcribed RNA sequence	145	145	48%	6e-33	27.38%	2414	GCXY01047385.1
TSA: Oncopeltus fasciatus breed wildtype s4503_L_10050_1_10_9_BUBBLE, transcribed RNA sequence	145	145	48%	8e-33	27.38%	2561	GCXY01047386.1
TSA: Oncopeltus fasciatus breed wildtype C229540_8_0, transcribed RNA sequence	101	101	19%	3e-21	32.99%	768	GCXY01031079.1
TSA: Oncopeltus fasciatus breed wildtype s5346_L_12344_0_33_9_BUBBLE, transcribed RNA sequence	103	103	35%	1e-19	24.57%	5337	GCXY01048321.1
TSA: Oncopeltus fasciatus breed wildtype s5347_L_12344_1_33_3_BUBBLE, transcribed RNA sequence	103	103	35%	1e-19	24.57%	5415	GCXY01048322.1
TSA: Oncopeltus fasciatus breed wildtype s13041_L_42026_0_4_6_LINEAR, transcribed RNA sequence	91.7	91.7	19%	4e-18	27.60%	654	GCXY01039041.1
TSA: Oncopeltus fasciatus breed wildtype C234674_36_0, transcribed RNA sequence	81.3	81.3	10%	7e-14	45.26%	909	GCXY01032090.1
TSA: Oncopeltus fasciatus breed wildtype s15616_L_54952_1_30_1_BUBBLE, transcribed RNA sequence	80.1	356	31%	2e-12	30.88%	5540	GCXY01041892.1
TSA: Oncopeltus fasciatus breed wildtype s15615_L_54952_0_31_0_BUBBLE, transcribed RNA sequence	76.3	351	30%	3e-11	31.13%	5140	GCXY01041891.1
TSA: Oncopeltus fasciatus breed wildtype C232320_5_0, transcribed RNA sequence	70.9	70.9	13%	2e-10	33.57%	837	GCXY01031638.1
TSA: Oncopeltus fasciatus breed wildtype s17776_L_67419_0_32_7_LINEAR, transcribed RNA sequence	57.4	105	17%	2e-05	39.76%	6470	GCXY01044285.1
TSA: Oncopeltus fasciatus breed wildtype C247316_8_0, transcribed RNA sequence	55.8	55.8	9%	3e-05	36.19%	1716	GCXY01034463.1
TSA: Oncopeltus fasciatus breed wildtype s15430_L_54057_0_7_0_LINEAR, transcribed RNA sequence	51.2	51.2	9%	0.001	29.29%	2964	GCXY01041686.1
TSA: Oncopeltus fasciatus breed wildtype	49.3	49.3	8%	0.004	37.80%	1718	GCXY01053109.1

Description	Max Score	Total Score	Query Cover	E value	Per. Ident	Acc. Len	Accession
s9660_L_27513_0_5_7_LINEAR, transcribed RNA sequence							
TSA: Oncopeltus fasciatus breed wildtype C250890_51_0, transcribed RNA sequence	49.3	49.3	10%	0.004	33.02%	2435	GCXY01035194.1
TSA: Oncopeltus fasciatus breed wildtype C252002_12_0, transcribed RNA sequence	47.0	47.0	6%	0.022	46.15%	3034	GCXY01035414.1

Graphic Summary

Distribution of the top 33 Blast Hits on 22 subject sequences

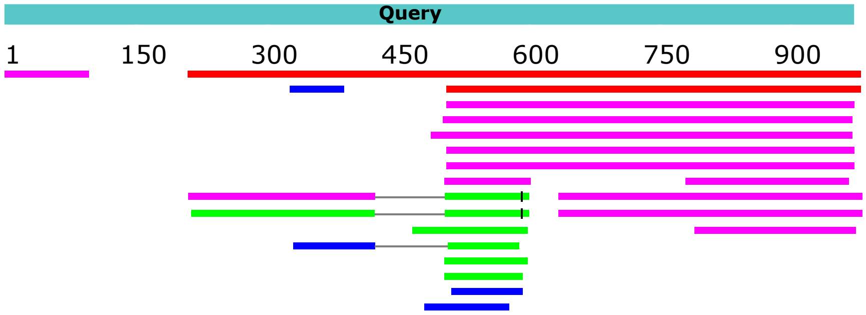

Alignments

Alignment view Pairwise

CDS feature

Restore defaults

TSA: Oncopeltus fasciatus breed wildtype s6732_L_16687_0_59_0_FORK, transcribed RNA sequence

Sequence ID: GCXY01049860.1 Length: 2401 Number of Matches: 1

Range 1: 101 to 2224

Score	Expect	Method	Identities	Positives	Gaps	Frame
963 bits(2489)	0.0()	Compositional matrix adjust.	513/769(67%)	604/769(78%)	65/769(8%)	-1

```
Query  206   VNGDDSWLYEDIQLERGNSGLGFSIAGGTDNPHIGTDTSIYITKLISGGAAAADGRLsin  265
             VNGD   + YE+I+LERG+SGLGFSIAGG+DNPH+G D  IYITKLI GGAAA DGRL +
Sbjct  2224  VNGDSDYEYEEIRLERGSSGLGFSIAGGSDNPHVGDDAGIYITKLIPGGAAAGDGRLRVG  2045

Query  266   diivsvndvsvvdvPHASAVDALKKAGNVVKLHVKRKrgtattpaagsaagdardsaasg  325
             D+I+ VN VSVVDV HA+AV+ALK+AGN V L+V+RKR   A
Sbjct  2044  DVILQVNRVSVVDVTHAAAVEALKRAGNTVTLYVRRKRDGAM------------------  1919

Query  326   pKVIEIDLVKGGKGLGFSIAGGIGNQHIPGDNGIYVTKLMDGGAAQVDGRLSIGDKLIAV  385
              ++IEI+L+KG KGLGFSIAGGIGNQHIPGDNGIYVTK+MDGGAAQVDGRL +GDKL+AV
Sbjct  1918  -RIIEIELIKGNKGLGFSIAGGIGNQHIPGDNGIYVTKIMDGGAAQVDGRLQVGDKLVAV  1742

Query  386   RT-NGSEKNLENVTHELAVATLKSITDKVTLIIGK--TQHlttsasggggggglssgqqls  442
             R      + NLENVTHE AVATLK+  ++V L++GK   T H   +
Sbjct  1741  RNMPDGDVNLENVTHEEAVATLKATHERVVLLVGKPDTPHSLSHPP-------------  1604

Query  443   qsqsqlatsqsqsqvhqqqhATPMVNSQSTEPGSRYASTNVLAAVPPGTPRAVSTEDIT-  501
                                P   + Q +    S +AST  L A     +P AVS ED++
Sbjct  1603  --------------------PDPSASPQPSLQDSLHASTVQLQAP---SPTAVSIEDLSS  1493

Query  502   REPRTITIQKGPQGLGFNIVGGEDGQGIYVSFILAGGPADLGSELKRGDQLLSVNNVNLT  561
             R+ RTI + KG  GLGFNIVGGEDG+GI+VSFILAGGPADL   +L+RGD++LSVN  +L+
Sbjct  1492  RDARTIILNKGSSGLGFNIVGGEDGEGIFVSFILAGGPADLSGDLRRGDRILSVNGQDLS  1313

Query  562   HATHEEAAQALKTSGGVVTLLAQYRPEEYNRFEARIQELKQQaalgaggsgtllrttQKR  621
              ATHEEAA ALK +G  VT++AQYRPEEYNRFEARI +LKQ  A+     + TLLRT+QKR
Sbjct  1312  TATHEEAAAALKGAGQEVTIVAQYRPEEYNRFEARIHDLKQ--AMSQQMTSTLLRTSQKR  1139

Query  622   SLYVRALFDYDPNRDDGLPSRGLPFKHGDILHVTNASDDEWWQARRVLGDNEDEQIGIVP  681
             +LYVRALF+YDPN+DDGLPSRGLPF  GDILHVTNASDDEWWQARRVL   E+E IGI+P
Sbjct  1138  TLYVRALFEYDPNKDDGLPSRGLPFHFGDILHVTNASDDEWWQARRVLPSGEEEGIGIIP  959

Query  682   SKRRWERKMRARDRSVKFQGHAAANNNLDKQSTLDRKKKNFTFSRKFPFMKSRDEKNEDG  741
             SKRRWERK RARDR+VKFQGH       +DKQSTL+RKKKNF+FSRKFPFMKS+D+K+E+G
Sbjct  958   SKRRWERKQRARDRTVKFQGHIPVM--IDKQSTLERKKKNFSFSRKFPFMKSKDDKSEEG  785

Query  742   SDQEPFMLCYTQDDANAEGASEENVLSYEAVQRLSINYTRPVIILGPLKDRINDDLISEY  801
             SDQEPFMLCY Q+D ++EG +EE VLSYE V +  ++Y RPVIILGPLKDRINDDLISE+
Sbjct  784   SDQEPFMLCYAQEDPSSEG-TEETVLSYEPVSQEVVSYARPVIILGPLKDRINDDLISEF  608

Query  802   PDKFGSCVPHTTRPKREYEVDGRDYHFVSSREQMERDIQNHLFIEAGQYNDNLYGTSVAS  861
             PD+FGSCVPHTTRPKREYEVDGRDYHFVSSRE+ME+DIQNHLFIEAGQYN+NLYGTSV+S
Sbjct  607   PDRFGSCVPHTTRPKREYEVDGRDYHFVSSREEMEKDIQNHLFIEAGQYNENLYGTSVSS  428

Query  862   VREVAEKGKHCILDVSGNAIKRLQVAQLYPVAVFIKPKSVDSVMEMNRRMTEEQAKKTYE  921
             V+EVAEKGKHCILDVSGNAIKRLQVA+L+P+A+FIKPKSV+SVMEMN+RMTEEQAKKT E
Sbjct  427   VKEVAEKGKHCILDVSGNAIKRLQVAKLHPIAIFIKPKSVESVMEMNKRMTEEQAKKTCE  248
```

```
Query  922   RAIKMEQEFGEYFTGVVQGDTIEEIYSKVKSMIWSQSGPTIWVPSKESL  970
             RA+K+EQ+FGEYFT +VQGDT EEIY KV  +I  QSGPTIWV +K+ L
Sbjct  247   RALKLEQDFGEYFTAIVQGDTPEEIYQKVNQVIQDQSGPTIWVSTKDPL  101
```

TSA: Oncopeltus fasciatus breed wildtype s6733_L_16687_1_60_6_FORK, transcribed RNA sequence

Sequence ID: **GCXY01049861.1** Length: 1617 Number of Matches: 1

Range 1: 101 to 1492

Score	Expect	Method	Identities	Positives	Gaps	Frame
729 bits(1881)	0.0()	Compositional matrix adjust.	357/469(76%)	415/469(88%)	5/469(1%)	-3

```
Query  502   REPRTITIQKGPQGLGFNIVGGEDGQGIYVSFILAGGPADLGSELKRGDQLLSVNNVNLT  561
             ++ RTI + KG  GLGFNIVGGEDG+GI+VSFILAGGPADL   +L+RGD++LSVN  +L+
Sbjct  1492  KDARTIILNKGSSGLGFNIVGGEDGEGIFVSFILAGGPADLSGDLRRGDRILSVNGQDLS  1313

Query  562   HATHEEAAQALKTSGGVVTLLAQYRPEEYNRFEARIQELKQQaalgaggsgtllrttQKR  621
              ATHEEAA ALK +G  VT++AQYRPEEYNRFEARI +LKQ  A+    + TLLRT+QKR
Sbjct  1312  TATHEEAAAALKGAGQEVTIVAQYRPEEYNRFEARIHDLKQ--AMSQQMTSTLLRTSQKR  1139

Query  622   SLYVRALFDYDPNRDDGLPSRGLPFKHGDILHVTNASDDEWWQARRVLGDNEDEQIGIVP  681
             +LYVRALF+YDPN+DDGLPSRGLPF  GDILHVTNASDDEWWQARRVL   E+E IGI+P
Sbjct  1138  TLYVRALFEYDPNKDDGLPSRGLPFHFGDILHVTNASDDEWWQARRVLPSGEEEGIGIIP  959

Query  682   SKRRWERKMRARDRSVKFQGHAAANNNLDKQSTLDRKKKNFTFSRKFPFMKSRDEKNEDG  741
             SKRRWERK RARDR+VKFQGH      +DKQSTL+RKKKNF+FSRKFPFMKS+D+K+E+G
Sbjct  958   SKRRWERKQRARDRTVKFQGHIPVM--IDKQSTLERKKKNFSFSRKFPFMKSKDDKSEEG  785

Query  742   SDQEPFMLCYTQDDANAEGASEENVLSYEAVQRLSINYTRPVIILGPLKDRINDDLISEY  801
             SDQEPFMLCY Q+D ++EG +EE VLSYE V +  ++Y RPVIILGPLKDRINDDLISE+
Sbjct  784   SDQEPFMLCYAQEDPSSEG-TEETVLSYEPVSQEVVSYARPVIILGPLKDRINDDLISEF  608

Query  802   PDKFGSCVPHTTRPKREYEVDGRDYHFVSSREQMERDIQNHLFIEAGQYNDNLYGTSVAS  861
             PD+FGSCVPHTTRPKREYEVDGRDYHFVSSRE+ME+DIQNHLFIEAGQYN+NLYGTSV+S
Sbjct  607   PDRFGSCVPHTTRPKREYEVDGRDYHFVSSREEMEKDIQNHLFIEAGQYNENLYGTSVSS  428

Query  862   VREVAEKGKHCILDVSGNAIKRLQVAQLYPVAVFIKPKSVDSVMEMNRRMTEEQAKKTYE  921
             V+EVAEKGKHCILDVSGNAIKRLQVA+L+P+A+FIKPKSV+SVMEMN+RMTEEQAKKT E
Sbjct  427   VKEVAEKGKHCILDVSGNAIKRLQVAKLHPIAIFIKPKSVESVMEMNKRMTEEQAKKTCE  248

Query  922   RAIKMEQEFGEYFTGVVQGDTIEEIYSKVKSMIWSQSGPTIWVPSKESL  970
             RA+K+EQ+FGEYFT +VQGDT EEIY KV  +I  QSGPTIWV +K+ L
Sbjct  247   RALKLEQDFGEYFTAIVQGDTPEEIYQKVNQVIQDQSGPTIWVSTKDPL  101
```

TSA: Oncopeltus fasciatus breed wildtype C252450_36_0, transcribed RNA sequence

Sequence ID: **GCXY01035507.1** Length: 3507 Number of Matches: 1

Range 1: 556 to 1839

Score	Expect	Method	Identities	Positives	Gaps	Frame
173 bits(438)	2e-41()	Compositional matrix adjust.	131/480(27%)	222/480(46%)	70/480(14%)	-1

```
Query  505   RTITIQKGPQGLGFNIVGGEDGQGIYVSFILAGGPADLGSELKRGDQLLSVNNVNLTHAT  564
             + I I+K  + LG  +    +G  + +  ++ GG AD    L  GD++L VN V +   +
Sbjct  1839  KIIKIEKTNEPLGATVRN--EGDSVIIGRVVRGGAADKSGLLHEGDEVLEVNGVEMRGKS  1666

Query  565   HEEAAQALKTSGGVVTLLAQYRPEEYNRFEARIQELKQQaalgaggsgtllrttQKRSLY  624
               E    L    G +T L    P+ +         L                      ++
Sbjct  1665  VNEVCDLLSAMTGTLTFLIVPAPDPHAHLHHHRDTL----------------------MH  1552

Query  625   VRALFDYDPNRDDGLPSR--GLPFKHGDILHVTNASDDEWWQARRVLGDNEDEQIGIVPS  682
             ++A F YDP  D  +P R  G+ F  GDILHV    D  WWQA R  G+ +    G++PS
Sbjct  1551  MKANFSYDPEDDLYIPCRELGISFMKGDILHVICQDDPHWWQAYRE-GEEDQTLAGLIPS  1375

Query  683   KRRWERKMRARDRSVKFQGHAAANNNLDKQSTLDRKKK---NFTFSRKFPFMKSRDEKNE  739
             K              + Q      +    + T D+ KK    F  ++K    K +   +E
Sbjct  1374  K------------GFQHQREVIKQSITGDKLTRDKSKKGGGTFLCAKKNTKKKKKKSYSE  1231

Query  740   DGSDQEPFMLCYTQDDANAEGASEENVLSYE--AVQRLSINYTRPVIILGP-------LK  790
              G     + +C     +N +    + +L+Y+  A+     N+ RP++++GP       L+
Sbjct  1230  SG-----YPIC----SSNTDDFDGDEILTYDEVALYYPRANHKRPIVLIGPPNIGRHELR  1078

Query  791   DRINDDLISEYPDKFGSCVPHTTRPKREYEVDGRDYHFVSSREQMERDIQNHLFIEAGQY  850
              R+ +D      D+F + +PHT+R ++E EVDG+DYHF++ R Q E DI    F+E G+Y
Sbjct  1077  QRLMED-----SDRFAAAIPHTSRARKEGEVDGQDYHFIT-RAQFEADILARKFVEHGEY  916

Query  851   NDNLYGTSVASVREVAEKGKHCILDVSGNAIKRLQVAQLYPVAVFIKPKSVDSVMEMNRR  910
                 YGTS+ ++R V   G+ C+L++   +++ L+ + L P  VF+ P S++ + +   R
Sbjct  915   ERAYYGTSLEAIRSVVNAGRICVLNLHPQSLRILRNSDLKPYVVFVNPPSLEKLRQKKIR  736

Query  911   MTE----EQAKKTYERAIKMEQEFGEYFTGVVQGDTIEEIYSKVKSMIWSQSGPTIWVPS  966
               E    E+ K+  E+A +ME ++G YF  ++     E  Y +++  I        WVP+
Sbjct  735   NGEAYKEEELKEIIEKAREMEDKYGHYFDMIIHNSDTERSYHQLREEINILEREPQWVPA  556
```

TSA: Oncopeltus fasciatus breed wildtype C219282_8_0, transcribed RNA sequence

Sequence ID: **GCXY01029008.1** Length: 592 Number of Matches: 1
