# Supplementary material for: The large milkweed bugs’ Na,K-ATPase β-subunits colocalize with septate junction proteins in a tissue-specific manner
Source: Cell Tissue Res. 2025 Mar 26;400(3):347–63. doi: 10.1007/s00441-025-03965-3 (PMC12125057; doi:10.1007/s00441-025-03965-3)
Supplement: Supplementary file 4 — Supplementary Material 4 (PDF 302 KB) [file 441_2025_3965_MOESM4_ESM.pdf]

The large milkweed bugs' Na,K-ATPase β-subunits colocalize with septate junction proteins in a tissue-specific manner

Marlena Herbertz[1*], Christian Lohr[2], Susanne Dobler[1]

[1] Institute of Cell and Systems Biology of Animals, Molecular Evolutionary Biology, Universität Hamburg, 20146 Hamburg, Germany

[2] Institute of Zell and Systems Biology of Animals, Neurophysiology, Universität Hamburg, 20146 Hamburg, Germany

*corresponding author: marlena.herbertz@uni-hamburg.de

The coracle protein sequence, also known as protein 4.1 homolog (UniProt: Q9V8R9 (EPB41_DROME) *Drosophila melanogaster*), were used as a reference to search via tblastn for potential homologs in a transcriptome of *O. fasciatus* using the transcriptome shotgun assembly (TSA) method. The coracle protein sequences of different insects were aligned with the coracle protein sequences of *D. melanogaster* (Diptera, Drosophilidae, UniProt accession numbers: Q9V8R9, A0A0B4LFX4, A0A0B4LG23) to search for conserved regions. The species included *Pararge aegeria* (Lepidoptera, Nymphalidae, UniProt accession numbers: S4NVH6, S4P6G9, S4PZT5), *Operophtera brumata* (Lepidoptera, Geometridae, UniProt accession numbers: A0A0L7KHN0, A0A0L7L3N5), *Danaus plexippus plexippus* (Lepidoptera, Nymphalidae, UniProt accession number: A0A212EJP2), *Corethrella appendiculata* (Diptera, Corethrellidae, UniProt accession number: W4VRQ9), *Cotesia congregata* (Hymenoptera, Braconidae, UniProt accession number: B1GS95), *Rhodnius prolixus* (Hemiptera, Reduviidae, UniProt accession number: G1K0N0) and *Oncopeltus fasciatus* (Hemiptera, Lygaeidae, NCBI TSA sequence ID: GCXY01047324.1). The multiple sequence alignment was performed with the online tool Clustal Omega provided by EMBL-EBI

Here are the results. The antibody epitope is highlighted in blue.

CLUSTAL O(1.2.4) multiple sequence alignment

```
sp|Q9V8R9|EPB41_DROME          MPAEIKP-SAP----------------------------AEP-----------------      13
tr|A0A0B4LFX4|A0A0B4LFX4_DROME MPAEIKP-SAP----------------------------AEP-----------------      13
tr|A0A0B4LG23|A0A0B4LG23_DROME MPAEIKP-SAP----------------------------AEP-----------------      13
GCXY01047324.1,                ------------------------------------------------------------     0
GCXY01047324.2,                ------------------------------------------------------------     0
tr|W4VRQ9|W4VRQ9_9DIPT         MPGESAKTVSP----------------------------TEP-----------------      14
tr|S4NVH6|S4NVH6_9NEOP         ------------------------------------------------------------     0
tr|S4P6G9|S4P6G9_9NEOP         MRESLRRLASDDGMTAR---LNAGRVRVELLTGEHITVDVER------------------      39
tr|S4PZT5|S4PZT5_9NEOP         ------------------------------------------------------------     0
tr|B1GS95|B1GS95_COTCN         ------------------------------------------------------------     0
tr|G1K0N0|G1K0N0_RHOPR         ------------------------------------------------------------     0
tr|A0A0L7KHN0|A0A0L7KHN0_9NEOP MPESVAKEAKV----------------------------AKS-----------------      14
tr|A0A0L7L3N5|A0A0L7L3N5_9NEOP ------------------------------------------------------------     0
tr|A0A212EJP2|A0A212EJP2_DANPL MPEGVAKEAKD----------------------------EKP-----------------      14
                                                 ***                         *****************

sp|Q9V8R9|EPB41_DROME          --------ETPTKSKPKSSSSSHGKPALARVTLLDGSLLDVSIDRKAIGRDVIN--SICA      63
tr|A0A0B4LFX4|A0A0B4LFX4_DROME --------ETPTKSKPKSSSSSHGKPALARVTLLDGSLLDVSIDRKAIGRDVIN--SICA      63
tr|A0A0B4LG23|A0A0B4LG23_DROME --------ETPTKSKPKSSSSSHGKPALARVTLLDGSLLDVSIDRKAIGRDVIN--SICA      63
GCXY01047324.1,                -----------------------GKPALAVVQLLDDKTLDVYVERKAKGEKIFD--EVCA      35
GCXY01047324.2,                ------------------------------------------------------------     0
tr|W4VRQ9|W4VRQ9_9DIPT         -------VATPTTPKKKQTNTSGGKAALAKVTLLDGSVLDVTIYRKATGRDLLN--SVCA      65
tr|S4NVH6|S4NVH6_9NEOP         ------------------------------------------------------------     0
tr|S4P6G9|S4P6G9_9NEOP         ---------------------------------------------KATGADLLDK--VCE      52
tr|S4PZT5|S4PZT5_9NEOP         ------------------------------------------------------------     0
tr|B1GS95|B1GS95_COTCN         ------------------------------------------------------------     0
tr|G1K0N0|G1K0N0_RHOPR         ------------------------------------------------------------     0
tr|A0A0L7KHN0|A0A0L7KHN0_9NEOP -------DAKAKAKEASPRRRPTGNLAKVLK--VAR----QHAGERGQGGQSGQ--ERCE      59
tr|A0A0L7L3N5|A0A0L7L3N5_9NEOP ------------------------------------------------------------     0
tr|A0A212EJP2|A0A212EJP2_DANPL -------KAKE------SPKRRSGNLARIKVELLDGSTMDLEADRKIRGHDLLS--KVCD      59
                               *******                                              *

sp|Q9V8R9|EPB41_DROME          GLNLIEKDY-FGLTYETPTDPR-----TWLDLEKPVS-----------------------      94
tr|A0A0B4LFX4|A0A0B4LFX4_DROME GLNLIEKDY-FGLTYETPTDPR-----TWLDLEKPVS-----------------------      94
tr|A0A0B4LG23|A0A0B4LG23_DROME GLNLIEKDY-FGLTYETPTDPR-----TWLDLEKPVS-----------------------      94
GCXY01047324.1,                HLDLREKDY-FGLNYRDKAGRR-----TWLDLDKRVG-----------------------      66
GCXY01047324.2,                ------------------------------------------------------------     0
tr|W4VRQ9|W4VRQ9_9DIPT         GLNILEKDY-FGLIYSTPNDPR-----VWLELEKPVG-----------------------      96
tr|S4NVH6|S4NVH6_9NEOP         ------------------------------------------------------------     0
tr|S4P6G9|S4P6G9_9NEOP         VLDVIESDY-FGLLHVQRGDP-----RVWVDLGRRLS-----------------------      83
tr|S4PZT5|S4PZT5_9NEOP         ------------------------------------------------------------     0
tr|B1GS95|B1GS95_COTCN         ------------------------------------------------------------     0
tr|G1K0N0|G1K0N0_RHOPR         ------------------------------------------------------------     0
```

```
tr|A0A0L7KHN0|A0A0L7KHN0_9NEOP      ------------------------------------------------------------    59
tr|A0A0L7L3N5|A0A0L7L3N5_9NEOP      ------------------------------------------------------------    0
tr|A0A212EJP2|A0A212EJP2_DANPL      SLNLVEKDY-FGLLYEDRGDPR----VWIDLDKRVS------------------------    90
                                             *            ****          ************************

sp|Q9V8R9|EPB41_DROME               ---KFFRTDTWPLTFAVKFYP------PEPSQLKEDITRYHLCLQVR-NDILEGRLPCTF    144
tr|A0A0B4LFX4|A0A0B4LFX4_DROME      ---KFFRTDTWPLTFAVKFYP------PEPSQLKEDITRYHLCLQVR-NDILEGRLPCTF    144
tr|A0A0B4LG23|A0A0B4LG23_DROME      ---KFFRTDTWPLTFAVKFYP------PEPSQLKEDITRYHLCLQVR-NDILEGRLPCTF    144
GCXY01047324.1,                     ---KVLKAEPWKLDFEVKFYP------KDPSQLQEDFTRYLLCLQVR-NDILSGKLPCSF    116
GCXY01047324.2,                     ------------------------------------------------------------    0
tr|W4VRQ9|W4VRQ9_9DIPT              ---KFFRSDPWNVTFAVKFYP------PEPAQLQEDITRYHLCLQVR-NDILEERLPSSF    146
tr|S4NVH6|S4NVH6_9NEOP              ------------------------------------------------------------    0
tr|S4P6G9|S4P6G9_9NEOP              ---KTFRNEPWDVKFTAKFYP------PEPSELRDDQSRYQLGLS---------------    119
tr|S4PZT5|S4PZT5_9NEOP              ------------------------------------RYQLGLS---VRRDLMEGRLTCSN    21
tr|B1GS95|B1GS95_COTCN              ------------------------------------------------------------    0
tr|G1K0N0|G1K0N0_RHOPR              ------------------------------------------------------------    0
tr|A0A0L7KHN0|A0A0L7KHN0_9NEOP      -----------------------------------GQSQGGVSPTPTHREPRQGRLPCST    84
tr|A0A0L7L3N5|A0A0L7L3N5_9NEOP      ------------------------------------------------------------    0
tr|A0A212EJP2|A0A212EJP2_DANPL      ---KMLKHEPWEVRFAVKFYP------PEPTQLQEELTRYQLVLAIRR-DLLEGRLPCST    140
                                    ***                  ******

sp|Q9V8R9|EPB41_DROME               VTHALLGSYLVQSEMGDYDAEEMPTRAYLKDFKIAP---------N--QTAELEDKVMDL    193
tr|A0A0B4LFX4|A0A0B4LFX4_DROME      VTHALLGSYLVQSEMGDYDAEEMPTRAYLKDFKIAP---------N--QTAELEDKVMDL    193
tr|A0A0B4LG23|A0A0B4LG23_DROME      VTHALLGSYLVQSEMGDYDAEEMPTRAYLKDFKIAP---------N--QTAELEDKVMDL    193
GCXY01047324.1,                     VTHAMLGSYLVQSQLGDYSAEEYPDASYLAGFKFCP---------DHDAAPGLEERVMTL    167
GCXY01047324.2,                     ------------------------------------------------------------    0
tr|W4VRQ9|W4VRQ9_9DIPT              VTHALLGSFLVQSELGDYDPIEMKDRAYLKDFKFAP---------H--QTPELEDKVIDL    195
tr|S4NVH6|S4NVH6_9NEOP              ------------------------------------------------------------    0
tr|S4P6G9|S4P6G9_9NEOP              ------------------------------------------------------------    119
tr|S4PZT5|S4PZT5_9NEOP              ITYALLASYVLQSEVGDREAR--VSTTLLSAHDSVP---------LHVLTPDLEEKIDEL    70
tr|B1GS95|B1GS95_COTCN              ------------------------------------------------------------    0
tr|G1K0N0|G1K0N0_RHOPR              ------------------------------------------------------------    0
tr|A0A0L7KHN0|A0A0L7KHN0_9NEOP      VTHALLASYLLQSELGDYDGKE-AGAGLCKQLKLVP---------PAACTPDLEEKVTEL    134
tr|A0A0L7L3N5|A0A0L7L3N5_9NEOP      ------------------------------------------------------------    0
tr|A0A212EJP2|A0A212EJP2_DANPL      VTHALLASYLLQSELGDYDQPAPG---LCKQLKLVP---------PAACTPELEEKVLEL    188
                                                                        *********

sp|Q9V8R9|EPB41_DROME               HKTHKGQSPAEAELHYLENAK--KLAMYGVDLHPAKDS-EGVDIMLGVCASG---LLVYR    247
tr|A0A0B4LFX4|A0A0B4LFX4_DROME      HKTHKGQSPAEAELHYLENAK--KLAMYGVDLHPAKDS-EGVDIMLGVCASG---LLVYR    247
tr|A0A0B4LG23|A0A0B4LG23_DROME      HKTHKGQSPAEAELHYLENAK--KLAMYGVDLHPAKDS-EGVDIMLGVCASG---LLVYR    247
GCXY01047324.1,                     HKGHKGQTPAEAELHYLENAK--KLSMYGVDMHPAKDS-EGVDILLGVNSTG---IQVYQ    221
GCXY01047324.2,                     ------------------------------------------------------------    0
tr|W4VRQ9|W4VRQ9_9DIPT              HKTHKGQTPAEAELNYLENAK--RLNLYGVDLHPAKDS-EGVDIFIGVCASG---LLVFK    249
tr|S4NVH6|S4NVH6_9NEOP              --------------------------MYGAEVHNVKDS-DDVDISLAVCAAG---ISVAR    30
tr|S4P6G9|S4P6G9_9NEOP              ------------------------------------------------------------    119
tr|S4PZT5|S4PZT5_9NEOP              YRK---------------------------------------------------------    73
tr|B1GS95|B1GS95_COTCN              ------------------------------------------------------------    0
```

```
tr|G1K0N0|G1K0N0_RHOPR           ------------------------------------------------------------    0
tr|A0A0L7KHN0|A0A0L7KHN0_9NEOP   HKTHNYLLQSEL--GDYEGKEAGTGLCKQLKLVPP-----------AACTPDLEEKVTEA    181
tr|A0A0L7L3N5|A0A0L7L3N5_9NEOP   ------------------------------------------------------------    0
tr|A0A212EJP2|A0A212EJP2_DANPL   YKTHRGQTPAEAELNYLENAK--KLAMYGVDLHPAKDS-ENVDITLGVCSSG---LLVHR    242
                                                                       *

sp|Q9V8R9|EPB41_DROME            DKLRINRFAWPKILKISYKRHHFY---IKIRPGEFEQYESTIGFKLANHRAAKKLWKSCV    304
tr|A0A0B4LFX4|A0A0B4LFX4_DROME   DKLRINRFAWPKILKISYKRHHFY---IKIRPGEFEQYESTIGFKLANHRAAKKLWKSCV    304
tr|A0A0B4LG23|A0A0B4LG23_DROME   DKLRINRFAWPKILKISYKRHHFY---IKIRPGEFEQYESTIGFKLANHRAAKKLWKSCV    304
GCXY01047324.1,                  EKLRINRFAWPKILKISYRRNNFY---IKIRPGEFEQYEQTVGFKLDSSRAAKNLWKVCV    278
GCXY01047324.2,                  ------------------------------------------------------------    0
tr|W4VRQ9|W4VRQ9_9DIPT           DKLRINRFAWPKILKISYKRSNFY---IKLRPGEFEQYESTVGFKLENHRAAKKLWKACV    306
tr|S4NVH6|S4NVH6_9NEOP           DGLVMNRFPWTKILKISYHKRVYT---LRLRASEFDEYETHLSFKLPSSRASKR------    81
tr|S4P6G9|S4P6G9_9NEOP           ------------------------------------------------------------    119
tr|S4PZT5|S4PZT5_9NEOP           ------------------------------------------------------------    73
tr|B1GS95|B1GS95_COTCN           --------------------------------------ESTIGFKLANHRAAKKLWKVCV    22
tr|G1K0N0|G1K0N0_RHOPR           ------------------------------------------------------------    0
tr|A0A0L7KHN0|A0A0L7KHN0_9NEOP   HKLRINRFAWPKILKISYKRHNFY---VKLRPGEFEQFESTVGFKLGNHRAAKKLWKTCS    238
tr|A0A0L7L3N5|A0A0L7L3N5_9NEOP   ------------------------------------------------------------    0
tr|A0A212EJP2|A0A212EJP2_DANPL   EKLRINRFAWPKILKISYKRHNFY---VKLRPGEFEQFESTVGFKLANHRAAKKLWKTCV    299
                                                         ***

sp|Q9V8R9|EPB41_DROME            EHHTFFRLMTPEPVS------KSK-MFPVFGSTY-RYKGRTQAESTNTP-----------    345
tr|A0A0B4LFX4|A0A0B4LFX4_DROME   EHHTFFRLMTPEPVS------KSK-MFPVFGSTY-RYKGRTQAESTNTP-----------    345
tr|A0A0B4LG23|A0A0B4LG23_DROME   EHHTFFRLMTPEPVS------KSK-MFPVFGSTY-RYKGRTQAESTNTP-----------    345
GCXY01047324.1,                  DQHTFFRLMSADATK------RPGLLFPRLGSLY-RFNRKTQAEVMVSG-----------    320
GCXY01047324.2,                  ------------------------------------------------------------    0
tr|W4VRQ9|W4VRQ9_9DIPT           EHHTFFRLMTPEPIT------NRG-IFPRLGSKF-RYSGRTHYETRQQP-----------    347
tr|S4NVH6|S4NVH6_9NEOP           ------------------------------------------------------------    81
tr|S4P6G9|S4P6G9_9NEOP           ------------------------------------------------------------    119
tr|S4PZT5|S4PZT5_9NEOP           ------------------------------------------------------------    73
tr|B1GS95|B1GS95_COTCN           EHHTFFRLMSPEPVK------KVG-LLPHLGSRF-RYSGRTHYETKKIP-----------    63
tr|G1K0N0|G1K0N0_RHOPR           ------------------------------------------------------------    0
tr|A0A0L7KHN0|A0A0L7KHN0_9NEOP   FTPTPNSVSTYTTVVHFIPSANSTDIIPEWRPPTEVFRGPF-------------------    279
tr|A0A0L7L3N5|A0A0L7L3N5_9NEOP   ------------------------------------------------------------    0
tr|A0A212EJP2|A0A212EJP2_DANPL   EHHTFFRLLSPEP-------VSRSTLFPRLGSRF-RYSGRTLHETRNEHPRRTQPTFARA    351

sp|Q9V8R9|EPB41_DROME            ------------------------------------------------------------    345
tr|A0A0B4LFX4|A0A0B4LFX4_DROME   ------------------------------------------------------------    345
tr|A0A0B4LG23|A0A0B4LG23_DROME   ------------------------------------------------------------    345
GCXY01047324.1,                  ------------------------------------------------------------    320
GCXY01047324.2,                  ------------------------------------------------------------    0
tr|W4VRQ9|W4VRQ9_9DIPT           ------------------------------------------------------------    347
tr|S4NVH6|S4NVH6_9NEOP           ------------------------------------------------------------    81
tr|S4P6G9|S4P6G9_9NEOP           ------------------------------------------------------------    119
tr|S4PZT5|S4PZT5_9NEOP           ------------------------------------------------------------    73
```

```
tr|B1GS95|B1GS95_COTCN              ------------------------------------------------------------        63
tr|G1K0N0|G1K0N0_RHOPR              ------------------------------------------------------------        0
tr|A0A0L7KHN0|A0A0L7KHN0_9NEOP      ------------------------------------------------------------        279
tr|A0A0L7L3N5|A0A0L7L3N5_9NEOP      -----------------NSVSVPALAAGEKEEPMPEASKRHTMPPQPAPRPTIKDKK---        40
tr|A0A212EJP2|A0A212EJP2_DANPL      LSHRR------LSSRSADALASG-----DKEEMPPEATKRHTMPPQPAPRPTVKDKK---        397
                                         ******                                              ***

sp|Q9V8R9|EPB41_DROME               ------------------------------------------------------------        345
tr|A0A0B4LFX4|A0A0B4LFX4_DROME      ------------------------------------------------------------        345
tr|A0A0B4LG23|A0A0B4LG23_DROME      ------------------------------------------------------------        345
GCXY01047324.1,                     -----------------------------------------NPRK---------------        324
GCXY01047324.2,                     ------------------------------------------------------------        0
tr|W4VRQ9|W4VRQ9_9DIPT              ------------------------------------------------------------        347
tr|S4NVH6|S4NVH6_9NEOP              ------------------------------------------------------------        81
tr|S4P6G9|S4P6G9_9NEOP              ------------------------------------------------------------        119
tr|S4PZT5|S4PZT5_9NEOP              ------------------------------------------------------------        73
tr|B1GS95|B1GS95_COTCN              -------------------------------------IERQP-----------PQFER--        73
tr|G1K0N0|G1K0N0_RHOPR              ------------------------------------------------------------        0
tr|A0A0L7KHN0|A0A0L7KHN0_9NEOP      ------------------------------------------------------------        279
tr|A0A0L7L3N5|A0A0L7L3N5_9NEOP      ----------------PPPGAVKVMPTAPDKKKEEKLVEKKIPAENGTETSSDPGITNNT        84
tr|A0A212EJP2|A0A212EJP2_DANPL      ----------------PPPGAVRVMPAPAR--------PNDTPAENGTDTTSDPGITNNV        433
                                    ****************

sp|Q9V8R9|EPB41_DROME               ------------------------------------------------------------        345
tr|A0A0B4LFX4|A0A0B4LFX4_DROME      ------------------------------------------------------------        345
tr|A0A0B4LG23|A0A0B4LG23_DROME      ------------------------------------------------------------        345
GCXY01047324.1,                     ------------------------------DGNFNRH----------------HSLG--P        336
GCXY01047324.2,                     ------------------------------------------------------------        0
tr|W4VRQ9|W4VRQ9_9DIPT              ------------------------------------------------------------        347
tr|S4NVH6|S4NVH6_9NEOP              ------------------------------------------------------------        81
tr|S4P6G9|S4P6G9_9NEOP              ------------------------------------------------------------        119
tr|S4PZT5|S4PZT5_9NEOP              ------------------------------------------------------------        73
tr|B1GS95|B1GS95_COTCN              ---------------SLS-------GRRLTSRSMDALGGSQTVETYG-------------        98
tr|G1K0N0|G1K0N0_RHOPR              ------------------------------------------------------------        0
tr|A0A0L7KHN0|A0A0L7KHN0_9NEOP      ------------------------------------------------------------        279
tr|A0A0L7L3N5|A0A0L7L3N5_9NEOP      EGTPKKSKVSVQTSTEISNFKFISKNVRLLTGGFGLFGGKKEKSPKEKEEKSPKSKDKSP        144
tr|A0A212EJP2|A0A212EJP2_DANPL      ETPR------------------------KSKGSFGLFGGKKEKSPKEEKSPKEKPLKIKE        469


sp|Q9V8R9|EPB41_DROME               ------------------------------------------------------------        345
tr|A0A0B4LFX4|A0A0B4LFX4_DROME      ------------------------------------------------------------        345
tr|A0A0B4LG23|A0A0B4LG23_DROME      ------------------------------------------------------------        345
GCXY01047324.1,                     SRSLEPL-----------------------------------------------------        343
GCXY01047324.2,                     ------------------------------------------------------------        0
tr|W4VRQ9|W4VRQ9_9DIPT              ------------------------------------------------------------        347
tr|S4NVH6|S4NVH6_9NEOP              ------------------------------------------------------------        81
tr|S4P6G9|S4P6G9_9NEOP              ------------------------------------------------------------        119
```

```
tr|S4PZT5|S4PZT5_9NEOP              ------------------------------------------------------------        73
tr|B1GS95|B1GS95_COTCN              ------------------------------------------------------------        98
tr|G1K0N0|G1K0N0_RHOPR              ------------------------------------------------------------        0
tr|A0A0L7KHN0|A0A0L7KHN0_9NEOP      ------------------------------------------------------------        279
tr|A0A0L7L3N5|A0A0L7L3N5_9NEOP      KADKSPKSKDKKAKEP--------------------------------------KTKVAV        166
tr|A0A212EJP2|A0A212EJP2_DANPL      KSDKSPKSKDKSDKSPKDKSPKDKVKDP--------------------------KAKVAV        503
                                                                **************************

sp|Q9V8R9|EPB41_DROME               -------------VDR-------------TPPKFNRT-----------------------        356
tr|A0A0B4LFX4|A0A0B4LFX4_DROME      -------------VDR-------------TPPKFNRT-----------------------        356
tr|A0A0B4LG23|A0A0B4LG23_DROME      -------------VDR-------------TPPKFNRT-----------------------        356
GCXY01047324.1,                     ------------------------------------------------------------        343
GCXY01047324.2,                     ------------------------------------------------------------        0
tr|W4VRQ9|W4VRQ9_9DIPT              -------------VDR-------------PAPDFKRS-----------------------        358
tr|S4NVH6|S4NVH6_9NEOP              ------------------------------------------------------------        81
tr|S4P6G9|S4P6G9_9NEOP              ------------------------------------------------------------        119
tr|S4PZT5|S4PZT5_9NEOP              ------------------------------------------------------------        73
tr|B1GS95|B1GS95_COTCN              -------------------------------------------------SEPSKRHTMSY        109
tr|G1K0N0|G1K0N0_RHOPR              -----------------------------------------------KKKKSSPSKGFSY        13
tr|A0A0L7KHN0|A0A0L7KHN0_9NEOP      ------------------------------------------------------------        279
tr|A0A0L7L3N5|A0A0L7L3N5_9NEOP      LDTSNVSSNPDNSREKSPAK----SPSKDEKPSFTKPYEYTDTERSPTRNKPFIQGAFSY        222
tr|A0A212EJP2|A0A212EJP2_DANPL      LDTSDVVSD--NSLDKSPVKDEKPS--------FTKPYEYTDTEKSPAR-KPLTKGAFSY        552

sp|Q9V8R9|EPB41_DROME               -------------LSGARLTSRSMDALALA----EKEKVA---------RKSSTLDHRGD        390
tr|A0A0B4LFX4|A0A0B4LFX4_DROME      -------------LSGARLTSRSMDALALA----EKEKVA---------RKSSTLDHRGD        390
tr|A0A0B4LG23|A0A0B4LG23_DROME      -------------LSGARLTSRSMDALALA----EKEKVA---------RKSSTLDHRGD        390
GCXY01047324.1,                     ------------------------------------------------------------        343
GCXY01047324.2,                     ------------------------------------------------------------        0
tr|W4VRQ9|W4VRQ9_9DIPT              -------------LTGKGLSSRSMDALALQ----KEKEAE---------KNVN-------        385
tr|S4NVH6|S4NVH6_9NEOP              ------------------------------------------------------------        81
tr|S4P6G9|S4P6G9_9NEOP              ------------------------------------------------------------        119
tr|S4PZT5|S4PZT5_9NEOP              ------------------------------------------------------------        73
tr|B1GS95|B1GS95_COTCN              EPEMI-PDMEH--IDQ--RPSI--------------------------------------        126
tr|G1K0N0|G1K0N0_RHOPR              EAKKEQSEDDESAAENQTTPVKVGVGLAFNYAPNAQELVKVDDEEENKR-----LSE---        65
tr|A0A0L7KHN0|A0A0L7KHN0_9NEOP      ------------------------------------------------------------        279
tr|A0A0L7L3N5|A0A0L7L3N5_9NEOP      EKEPI-SDEKQRAADEAQSPTTRKAGLAFNYAPGEDKKLAESAEKRKTPEDPSKLKTPGL        281
tr|A0A212EJP2|A0A212EJP2_DANPL      DKEPV-SDEKQRALDGAQSPGTRKAGLAFNYAPGEEKKVAESAEKRKTPDDLSKLKTPGI        611

sp|Q9V8R9|EPB41_DROME               R------------------------------------NADGDAHSRSPIK----------        404
tr|A0A0B4LFX4|A0A0B4LFX4_DROME      R------------------------------------NADGDAHSRSPIK----------        404
tr|A0A0B4LG23|A0A0B4LG23_DROME      R------------------------------------NADGDAHSRSPIK----------        404
GCXY01047324.1,                     ------------------------------------------------------------        343
GCXY01047324.2,                     ------------------------------------------------------------        0
tr|W4VRQ9|W4VRQ9_9DIPT              ------------------------------------------------------------        385
tr|S4NVH6|S4NVH6_9NEOP              ------------------------------------------------------------        81
```

```
tr|S4P6G9|S4P6G9_9NEOP              ------------------------------------------------------------        119
tr|S4PZT5|S4PZT5_9NEOP              ------------------------------------------------------------        73
tr|B1GS95|B1GS95_COTCN              ------------------------------------------------------------        126
tr|G1K0N0|G1K0N0_RHOPR              -----RLIEEES---------------KNSLPPEVVVEE---EHKKVQVDVVKD----V-        97
tr|A0A0L7KHN0|A0A0L7KHN0_9NEOP      ------------------------------------------------------------        279
tr|A0A0L7L3N5|A0A0L7L3N5_9NEOP      DYVQSALLKEQA---------------KNLIDPTLALLDSERAHHEAPVAAAVAVPLTAA        326
tr|A0A212EJP2|A0A212EJP2_DANPL      DYVQSAALKETAKSP-----------RSNLIDPTLALLDSERAHHETPVAIAP-----VA        655
                                                   ***********

sp|Q9V8R9|EPB41_DROME               ----------------NKKEK---------------------------------------        409
tr|A0A0B4LFX4|A0A0B4LFX4_DROME      ----------------NKKEKNP------------------------------------N        412
tr|A0A0B4LG23|A0A0B4LG23_DROME      ----------------NKKEK---------------------------------------        409
GCXY01047324.1,                     ------------------------------------------------------------        343
GCXY01047324.2,                     ------------------------------------------------------------        0
tr|W4VRQ9|W4VRQ9_9DIPT              ------------------------------------------------------------        385
tr|S4NVH6|S4NVH6_9NEOP              ------------------------------------------------------------        81
tr|S4P6G9|S4P6G9_9NEOP              ------------------------------------------------------------        119
tr|S4PZT5|S4PZT5_9NEOP              ------------------------------------------------------------        73
tr|B1GS95|B1GS95_COTCN              ------------------------------------------------------------        126
tr|G1K0N0|G1K0N0_RHOPR              KKATDDTTKHRKSGGFG-------------------------------------------        114
tr|A0A0L7KHN0|A0A0L7KHN0_9NEOP      ------------------------------------------------------------        279
tr|A0A0L7L3N5|A0A0L7L3N5_9NEOP      KPAENEIQVVIITGRYNPKSKKLDDANGTVLVTKGTINRANGKIQTDKEQINTKSGQVIF        386
tr|A0A212EJP2|A0A212EJP2_DANPL      KKSDNEIQVVIITGRYNPKTKKLDDANGTILVTKGRLDKSTSKIQTEKELINTKSGQINY        715

sp|Q9V8R9|EPB41_DROME               -------DADKEAK-------------L----REKKQ-----------KEKEEKERKER-        433
tr|A0A0B4LFX4|A0A0B4LFX4_DROME      ELYLDLMDADKEAK-------------L----REKKQ-----------KEKEEKERKER-        443
tr|A0A0B4LG23|A0A0B4LG23_DROME      -------DADKEAK-------------L----REKKQ-----------KEKEEKERKER-        433
GCXY01047324.1,                     ------------------------------------------------------------        343
GCXY01047324.2,                     ------------------------------------------------------------        0
tr|W4VRQ9|W4VRQ9_9DIPT              ------KDANKRHTMSHPPDHIP---DLDSPTRGSRSP----------IKKDKKERKPVG        426
tr|S4NVH6|S4NVH6_9NEOP              ------------------------------------------------------------        81
tr|S4P6G9|S4P6G9_9NEOP              ------------------------------------------------------------        119
tr|S4PZT5|S4PZT5_9NEOP              ------------------------------------------------------------        73
tr|B1GS95|B1GS95_COTCN              ------------------------------------------------------------        126
tr|G1K0N0|G1K0N0_RHOPR              ------------------------------------------------------------        114
tr|A0A0L7KHN0|A0A0L7KHN0_9NEOP      ------------------------------------------------------------        279
tr|A0A0L7L3N5|A0A0L7L3N5_9NEOP      TDPVTGKQETKNGHLDSKSGHILFTSGVIDPKTGKIDPTLAQQYCFVEKSKDKVGAKPGR        446
tr|A0A212EJP2|A0A212EJP2_DANPL      TDPTTGKEDVKQGHVDSKTGHILFTSGVFDPKTGKLDPTLAQQYCFVEKLEDKVGNKPGR        775

sp|Q9V8R9|EPB41_DROME               ------------------------------------------------------------        433
tr|A0A0B4LFX4|A0A0B4LFX4_DROME      ------------------------------------------------------------        443
tr|A0A0B4LG23|A0A0B4LG23_DROME      ------------------------------------------------------------        433
GCXY01047324.1,                     ------------------------------------------------------------        343
GCXY01047324.2,                     ------------------------------------------------------------        0
tr|W4VRQ9|W4VRQ9_9DIPT              GVSVLPVAGKKDKD----------------------------------------------        440
```

```
tr|S4NVH6|S4NVH6_9NEOP              ------------------------------------------------------------        81
tr|S4P6G9|S4P6G9_9NEOP              ------------------------------------------------------------        119
tr|S4PZT5|S4PZT5_9NEOP              ------------------------------------------------------------        73
tr|B1GS95|B1GS95_COTCN              ------------------------------------------------------------        126
tr|G1K0N0|G1K0N0_RHOPR              ------------------------------------------------------------        114
tr|A0A0L7KHN0|A0A0L7KHN0_9NEOP      ------------------------------------------------------------        279
tr|A0A0L7L3N5|A0A0L7L3N5_9NEOP      EVDLVVITGKYDGKHKRLDVSHGHVDVSRAVVGSEGTVSSNYGVIDPRSGKIDYIDPKTG        506
tr|A0A212EJP2|A0A212EJP2_DANPL      EVDLVVITGKYDGKHKKLDASHGHVEVSKAIVAPDGTVHSNYGLIDPQKGKIDLIDVKTG        835


sp|Q9V8R9|EPB41_DROME               ------------------------------------------------------------        433
tr|A0A0B4LFX4|A0A0B4LFX4_DROME      ------------------------------------------------------------        443
tr|A0A0B4LG23|A0A0B4LG23_DROME      ------------------------------------------------------------        433
GCXY01047324.1,                     ------------------------------------------------------------        343
GCXY01047324.2,                     ------------------------------------------------------------        0
tr|W4VRQ9|W4VRQ9_9DIPT              ------------------------------------------------------------        440
tr|S4NVH6|S4NVH6_9NEOP              ------------------------------------------------------------        81
tr|S4P6G9|S4P6G9_9NEOP              ------------------------------------------------------------        119
tr|S4PZT5|S4PZT5_9NEOP              ------------------------------------------------------------        73
tr|B1GS95|B1GS95_COTCN              ------------------------------------------------------------        126
tr|G1K0N0|G1K0N0_RHOPR              ------------------------------------------------------------        114
tr|A0A0L7KHN0|A0A0L7KHN0_9NEOP      ------------------------------------------------------------        279
tr|A0A0L7L3N5|A0A0L7L3N5_9NEOP      KLEPKQAYVDLKTGNILVTTGVTDPKSGKVDSSLGQQFSIVEKDATKA-NREVRLVIVTS        565
tr|A0A212EJP2|A0A212EJP2_DANPL      KQDPKQAYVDQKTGNLLVTTGVHDPKSGKVDSSLGQQFSIVEKDATKA-NREVRLVVVTS        894
                                                                                    *

sp|Q9V8R9|EPB41_DROME               ---------------------------------------------------EKRELEEKK        442
tr|A0A0B4LFX4|A0A0B4LFX4_DROME      ---------------------------------------------------EKRELEEKK        452
tr|A0A0B4LG23|A0A0B4LG23_DROME      ---------------------------------------------------EKRELEEKK        442
GCXY01047324.1,                     ------------------------------------------------------------        343
GCXY01047324.2,                     ------------------------------------------------------------        0
tr|W4VRQ9|W4VRQ9_9DIPT              ---------------------------------------------------GKDGIKETN        449
tr|S4NVH6|S4NVH6_9NEOP              ------------------------------------------------------------        81
tr|S4P6G9|S4P6G9_9NEOP              ------------------------------------------------------------        119
tr|S4PZT5|S4PZT5_9NEOP              ------------------------------------------------------------        73
tr|B1GS95|B1GS95_COTCN              ------------------------------------------------------------        126
tr|G1K0N0|G1K0N0_RHOPR              ------------------------------------------------------------        114
tr|A0A0L7KHN0|A0A0L7KHN0_9NEOP      ------------------------------------------------------------        279
tr|A0A0L7L3N5|A0A0L7L3N5_9NEOP      KYDLKNKKLDPTFAHVDSVKGVLSGTDGKIYTEYGVIDPRTGEIQITDSATGKQEVKHAI        625
tr|A0A212EJP2|A0A212EJP2_DANPL      KYDLKNKKLEPSFAHIDSIKGVLSGTDGKIYTEYGVIDPRTGDILVTDSKTGKQEIKRAT        954


sp|Q9V8R9|EPB41_DROME               KAEKAAKAALAAGA--------------------------------------AAGAAVNG        464
tr|A0A0B4LFX4|A0A0B4LFX4_DROME      KAEKAAKAALAAGA--------------------------------------AAGAAVNG        474
tr|A0A0B4LG23|A0A0B4LG23_DROME      KAEKAAKAALAAGA--------------------------------------AAGAAVNG        464
GCXY01047324.1,                     ------------------------------------------------------------        343
GCXY01047324.2,                     ------------------------------------------------------------        0
```

```
tr|W4VRQ9|W4VRQ9_9DIPT              DVKKDGKDEI------------------------------------------VNGNNAAQ        467
tr|S4NVH6|S4NVH6_9NEOP              ------------------------------------------------------------        81
tr|S4P6G9|S4P6G9_9NEOP              ------------------------------------------------------------        119
tr|S4PZT5|S4PZT5_9NEOP              ------------------------------------------------------------        73
tr|B1GS95|B1GS95_COTCN              ------------------------------------------------------------        126
tr|G1K0N0|G1K0N0_RHOPR              ------------------------------------------------------------        114
tr|A0A0L7KHN0|A0A0L7KHN0_9NEOP      ------------------------------------------------------------        279
tr|A0A0L7L3N5|A0A0L7L3N5_9NEOP      VDPKTGNILLLSGVIDPRTNKLDSSLGQQYSIVDKPTDTFGALPGKEVQVVAITGKYDSK        685
tr|A0A212EJP2|A0A212EJP2_DANPL      VDPKTGNILLLSGVIDPRTGQLDTTLGQQYSIVDKPTVNFASLPGREVQVVAITGKYDAK        1014

sp|Q9V8R9|EPB41_DROME               NDELNDSNK-------------------------------------------SDKSSGRR        481
tr|A0A0B4LFX4|A0A0B4LFX4_DROME      NDELNDSNK-------------------------------------------SDKSSGRR        491
tr|A0A0B4LG23|A0A0B4LG23_DROME      NDELNDSNK-------------------------------------------SDKSSGRR        481
GCXY01047324.1,                     ------------------------------------------------------------        343
GCXY01047324.2,                     ------------------------------------------------------------        0
tr|W4VRQ9|W4VRQ9_9DIPT              NDTLNSSTE-------------------------------------------SKSPPGKR        484
tr|S4NVH6|S4NVH6_9NEOP              ------------------------------------------------------------        81
tr|S4P6G9|S4P6G9_9NEOP              ------------------------------------------------------------        119
tr|S4PZT5|S4PZT5_9NEOP              ------------------------------------------------------------        73
tr|B1GS95|B1GS95_COTCN              ------------------------------------------------------------        126
tr|G1K0N0|G1K0N0_RHOPR              ------------------------------------------------------------        114
tr|A0A0L7KHN0|A0A0L7KHN0_9NEOP      ------------------------------------------------------------        279
tr|A0A0L7L3N5|A0A0L7L3N5_9NEOP      NKKLDNPNGFVETSQGIISDKDGKVHTNFGVLDPNTGKIHYTDPKTGKRDSKQANIDPKT        745
tr|A0A212EJP2|A0A212EJP2_DANPL      NKKLDNPNGFVETSQAIVSDKDSKVHTNFGILDPNSGKIYYTDPKTGKRDSKQALLDPKN        1074

sp|Q9V8R9|EPB41_DROME               GVGIFSSGRKSKSGSPS---------KDGKD-KSGKDKDKEVGRLGLV-VTSGLGDNQQD        530
tr|A0A0B4LFX4|A0A0B4LFX4_DROME      GVGIFSSGRKSKSGSPS---------KDGKD-KSGKDKDKEVGRLGLV-VTSGLGDNQQD        540
tr|A0A0B4LG23|A0A0B4LG23_DROME      GVGIFSSGRKSKSGSPS---------KDGKD-KSGKDKDKEVGRLGLV-VTSGLGDNQQD        530
GCXY01047324.1,                     ------------------------------------------------------------        343
GCXY01047324.2,                     ------------------------------------------------------------        0
tr|W4VRQ9|W4VRQ9_9DIPT              RGFLFSSGRKSPKEKAE---------KVEKLAKAGSKGDS---PTKDT-VAAAAGDKQKQ        531
tr|S4NVH6|S4NVH6_9NEOP              ------------------------------------------------------------        81
tr|S4P6G9|S4P6G9_9NEOP              ------------------------------------------------------------        119
tr|S4PZT5|S4PZT5_9NEOP              ------------------------------------------------------------        73
tr|B1GS95|B1GS95_COTCN              ------------------------------------------------------------        126
tr|G1K0N0|G1K0N0_RHOPR              ------------------------------------------------------------        114
tr|A0A0L7KHN0|A0A0L7KHN0_9NEOP      ------------------------------------------------------------        279
tr|A0A0L7L3N5|A0A0L7L3N5_9NEOP      GSFILTSGVIDPKTGKADSSLAQQLAVVDKDAPKGIP---ER-LANLVIVTAKYDPKSKK        801
tr|A0A212EJP2|A0A212EJP2_DANPL      GSLLLTTGVIDPKTGKTDSSLAQQLTVVDKDAPQGIR---ER-QVNLVIVTSKYDLKNKK        1130

sp|Q9V8R9|EPB41_DROME               QNL---------------------------------------------------------        533
tr|A0A0B4LFX4|A0A0B4LFX4_DROME      QNL---------------------------------------------------------        543
tr|A0A0B4LG23|A0A0B4LG23_DROME      QNL---------------------------------------------------------        533
GCXY01047324.1,                     ------------------------------------------------------------        343
```

```
GCXY01047324.2,                  ------------------------------------------------------------      0
tr|W4VRQ9|W4VRQ9_9DIPT           KDK---------------------------------------------------------      534
tr|S4NVH6|S4NVH6_9NEOP           ------------------------------------------------------------      81
tr|S4P6G9|S4P6G9_9NEOP           ------------------------------------------------------------      119
tr|S4PZT5|S4PZT5_9NEOP           ------------------------------------------------------------      73
tr|B1GS95|B1GS95_COTCN           ------------------------------------------------------------      126
tr|G1K0N0|G1K0N0_RHOPR           ------------------------------------------------------------      114
tr|A0A0L7KHN0|A0A0L7KHN0_9NEOP   ------------------------------------------------------------      279
tr|A0A0L7L3N5|A0A0L7L3N5_9NEOP   LDVTNAHVDSVPGKYDDDD-KVHSAFGIVDPSTGEIIVTDPISGKQEVKKSSIDPKTGNL      860
tr|A0A212EJP2|A0A212EJP2_DANPL   LDLTNPHINTVT------------------------------------------------      1142
                                                    *

sp|Q9V8R9|EPB41_DROME            -------------------------D----------------------------------      534
tr|A0A0B4LFX4|A0A0B4LFX4_DROME   -------------------------D----------------------------------      544
tr|A0A0B4LG23|A0A0B4LG23_DROME   -------------------------D----------------------------------      534
GCXY01047324.1,                  ------------------------------------------------------------      343
GCXY01047324.2,                  ------------------------------------------------------------      0
tr|W4VRQ9|W4VRQ9_9DIPT           -------------------------EKEKDKTKPV-------------------------      544
tr|S4NVH6|S4NVH6_9NEOP           ------------------------------------------------------------      81
tr|S4P6G9|S4P6G9_9NEOP           ------------------------------------------------------------      119
tr|S4PZT5|S4PZT5_9NEOP           ------------------------------------------------------------      73
tr|B1GS95|B1GS95_COTCN           ------------------------------------------------------------      126
tr|G1K0N0|G1K0N0_RHOPR           ------------------------------------------------------------      114
tr|A0A0L7KHN0|A0A0L7KHN0_9NEOP   ------------------------------------------------------------      279
tr|A0A0L7L3N5|A0A0L7L3N5_9NEOP   LLTSGVIDPRTGQHDSSLGQQITVIDKPKDRFAAVPGKEVQLVIITSKYDSKYKRLDNPN      920
tr|A0A212EJP2|A0A212EJP2_DANPL   ------------------------------------------------------------      1142

sp|Q9V8R9|EPB41_DROME            ------------------------------------------------------------      534
tr|A0A0B4LFX4|A0A0B4LFX4_DROME   ------------------------------------------------------------      544
tr|A0A0B4LG23|A0A0B4LG23_DROME   ------------------------------------------------------------      534
GCXY01047324.1,                  ------------------------------------------------------------      343
GCXY01047324.2,                  ------------------------------------------------------------      0
tr|W4VRQ9|W4VRQ9_9DIPT           ------------------------------------------------------------      544
tr|S4NVH6|S4NVH6_9NEOP           ------------------------------------------------------------      81
tr|S4P6G9|S4P6G9_9NEOP           ------------------------------------------------------------      119
tr|S4PZT5|S4PZT5_9NEOP           ------------------------------------------------------------      73
tr|B1GS95|B1GS95_COTCN           ------------------------------------------------------------      126
tr|G1K0N0|G1K0N0_RHOPR           ------------------------------------------------------------      114
tr|A0A0L7KHN0|A0A0L7KHN0_9NEOP   ------------------------------------------------------------      279
tr|A0A0L7L3N5|A0A0L7L3N5_9NEOP   GHVECSRGIVSSDGKVHTNFGVIDPRTGKVEYVDPKTGKQEIKQAVADPKTGHLIISSGV      980
tr|A0A212EJP2|A0A212EJP2_DANPL   ------------------------------------------------------------      1142

sp|Q9V8R9|EPB41_DROME            -----------------------------------EAARNA-------------------      540
tr|A0A0B4LFX4|A0A0B4LFX4_DROME   -----------------------------------EAARNA-------------------      550
tr|A0A0B4LG23|A0A0B4LG23_DROME   -----------------------------------EAARNA-------------------      540
```

```
GCXY01047324.1,                      ------------------------------------------------------------      343
GCXY01047324.2,                      ------------------------------------------------------------      0
tr|W4VRQ9|W4VRQ9_9DIPT               ----------------------------------AVGVDIK-------------------      551
tr|S4NVH6|S4NVH6_9NEOP               ------------------------------------------------------------      81
tr|S4P6G9|S4P6G9_9NEOP               ------------------------------------------------------------      119
tr|S4PZT5|S4PZT5_9NEOP               ------------------------------------------------------------      73
tr|B1GS95|B1GS95_COTCN               ------------------------------------------------------------      126
tr|G1K0N0|G1K0N0_RHOPR               ------------------------------------------------------------      114
tr|A0A0L7KHN0|A0A0L7KHN0_9NEOP       ------------------------------------------------------------      279
tr|A0A0L7L3N5|A0A0L7L3N5_9NEOP       ADPKTGKTDSSLAQQLTIVDRDSKGAPEKLVNLVIVTAKYDVKNKKLDLANAHVDTIPGK      1040
tr|A0A212EJP2|A0A212EJP2_DANPL       ------------------------------------------------------------      1142


sp|Q9V8R9|EPB41_DROME                ---AK-------------------------------------------------------      542
tr|A0A0B4LFX4|A0A0B4LFX4_DROME       ---AK-------------------------------------------------------      552
tr|A0A0B4LG23|A0A0B4LG23_DROME       ---AK-------------------------------------------------------      542
GCXY01047324.1,                      ------------------------------------------------------------      343
GCXY01047324.2,                      ------------------------------------------------------------      0
tr|W4VRQ9|W4VRQ9_9DIPT               ---VD-------------------------------------------------------      553
tr|S4NVH6|S4NVH6_9NEOP               ------------------------------------------------------------      81
tr|S4P6G9|S4P6G9_9NEOP               ------------------------------------------------------------      119
tr|S4PZT5|S4PZT5_9NEOP               ------------------------------------------------------------      73
tr|B1GS95|B1GS95_COTCN               ------------------------------------------------------------      126
tr|G1K0N0|G1K0N0_RHOPR               ------------------------------------------------------------      114
tr|A0A0L7KHN0|A0A0L7KHN0_9NEOP       ------------------------------------------------------------      279
tr|A0A0L7L3N5|A0A0L7L3N5_9NEOP       IG-ADDKVHTAFGVVDPNTGDIVVTDPITGKQEIKKASLDPKTGNLLLTSSVVDPLSGQV      1099
tr|A0A212EJP2|A0A212EJP2_DANPL       ------------------------------------------------------------      1142
                                       *

sp|Q9V8R9|EPB41_DROME                ------------------------------------------------------------      542
tr|A0A0B4LFX4|A0A0B4LFX4_DROME       ------------------------------------------------------------      552
tr|A0A0B4LG23|A0A0B4LG23_DROME       ------------------------------------------------------------      542
GCXY01047324.1,                      ------------------------------------------------------------      343
GCXY01047324.2,                      ------------------------------------------------------------      0
tr|W4VRQ9|W4VRQ9_9DIPT               ------------------------------------------------------------      553
tr|S4NVH6|S4NVH6_9NEOP               ------------------------------------------------------------      81
tr|S4P6G9|S4P6G9_9NEOP               ------------------------------------------------------------      119
tr|S4PZT5|S4PZT5_9NEOP               ------------------------------------------------------------      73
tr|B1GS95|B1GS95_COTCN               ------------------------------------------------------------      126
tr|G1K0N0|G1K0N0_RHOPR               ------------------------------------------------------------      114
tr|A0A0L7KHN0|A0A0L7KHN0_9NEOP       ------------------------------------------------------------      279
tr|A0A0L7L3N5|A0A0L7L3N5_9NEOP       DPTLGQQISVVDKPKDRFATVPGKEVQLVVITSKYDPKNKRLDNPNGYVEVSRGIIAP-D      1158
tr|A0A212EJP2|A0A212EJP2_DANPL       ------------------------------------------------------------      1142
                                                                                               *

sp|Q9V8R9|EPB41_DROME                --------------------------------------------------NRGSTTPGVT      552
tr|A0A0B4LFX4|A0A0B4LFX4_DROME       --------------------------------------------------NRGSTTPGVT      562
```

```
tr|A0A0B4LG23|A0A0B4LG23_DROME      --------------------------------------------------NRGSTTPGVT      552
GCXY01047324.1,                     ------------------------------------------------------------      343
GCXY01047324.2,                     ------------------------------------------------------------      0
tr|W4VRQ9|W4VRQ9_9DIPT              --------------------------------------------------EKQQQKPSVT      563
tr|S4NVH6|S4NVH6_9NEOP              ------------------------------------------------------------      81
tr|S4P6G9|S4P6G9_9NEOP              ------------------------------------------------------------      119
tr|S4PZT5|S4PZT5_9NEOP              ------------------------------------------------------------      73
tr|B1GS95|B1GS95_COTCN              ------------------------------------------------------------      126
tr|G1K0N0|G1K0N0_RHOPR              ------------------------------------------------------------      114
tr|A0A0L7KHN0|A0A0L7KHN0_9NEOP      ------------------------------------------------------------      279
tr|A0A0L7L3N5|A0A0L7L3N5_9NEOP      GRIHTNFGIVDTKTGKIDQIDPRTGKSETKQASTDSKTGHLFITSGVIDPKTGKTDPSLA      1218
tr|A0A212EJP2|A0A212EJP2_DANPL      ------------------------------------------------------------      1142

sp|Q9V8R9|EPB41_DROME               RQYEYAVDNDGNTSPTRKSY--------------------------TPGGFRYD------      580
tr|A0A0B4LFX4|A0A0B4LFX4_DROME      RQYEYAVDNDGNTSPTRKSY--------------------------TPGGFRYD------      590
tr|A0A0B4LG23|A0A0B4LG23_DROME      RQYEYAVDNDGNTSPTRKSY--------------------------TPGGFRYD------      580
GCXY01047324.1,                     ------------------------------------------------------------      343
GCXY01047324.2,                     ------------------------------------------------------------      0
tr|W4VRQ9|W4VRQ9_9DIPT              KPYEYQE-QDPNTSPTKKNY--------------------------VKGGFKYD------      590
tr|S4NVH6|S4NVH6_9NEOP              ------------------------------------------------------------      81
tr|S4P6G9|S4P6G9_9NEOP              ------------------------------------------------------------      119
tr|S4PZT5|S4PZT5_9NEOP              ------------------------------------------------------------      73
tr|B1GS95|B1GS95_COTCN              ------------------------------------------------------------      126
tr|G1K0N0|G1K0N0_RHOPR              ------------------------------------------------------------      114
tr|A0A0L7KHN0|A0A0L7KHN0_9NEOP      ------------------------------------------------------------      279
tr|A0A0L7L3N5|A0A0L7L3N5_9NEOP      QQFAVVDKDAPR-----ERYVNLVIVTSKYDLKNKKLDLANAHVDTIPGKVGADGNVHTE      1273
tr|A0A212EJP2|A0A212EJP2_DANPL      ------------------------------------------------GTIDDNDKVHTE      1154

sp|Q9V8R9|EPB41_DROME               -------------QDPNSRKS-----GAD---G------------QEQLSPTSQ-----Q      602
tr|A0A0B4LFX4|A0A0B4LFX4_DROME      -------------QDPNSRKS-----GAD---G------------QEQLSPTSQ-----Q      612
tr|A0A0B4LG23|A0A0B4LG23_DROME      -------------QDPNSRKS-----GAD---G------------QEQLSPTSQ-----Q      602
GCXY01047324.1,                     ------------------------------------------------------------      343
GCXY01047324.2,                     ------------------------------------------------------------      0
tr|W4VRQ9|W4VRQ9_9DIPT              -------------EDPNLRNK-----TND-----------------EQLSPNSQ----TR      611
tr|S4NVH6|S4NVH6_9NEOP              ------------------------------------------------------------      81
tr|S4P6G9|S4P6G9_9NEOP              ------------------------------------------------------------      119
tr|S4PZT5|S4PZT5_9NEOP              ------------------------------------------------------------      73
tr|B1GS95|B1GS95_COTCN              ------------------------------------------------------------      126
tr|G1K0N0|G1K0N0_RHOPR              ------------------------------------------------------------      114
tr|A0A0L7KHN0|A0A0L7KHN0_9NEOP      ------------------------------------------------------------      279
tr|A0A0L7L3N5|A0A0L7L3N5_9NEOP      FGVVDPVTGDITITDPVTGKRETKKASVDPKTGNLVLTSGVVDPVTGQVDPTLAQQMSVV      1333
tr|A0A212EJP2|A0A212EJP2_DANPL      LGIIDPATGQITITDPVSGKQEVKKATVDSKTGNMLLTSGVIDPNTGVVDPTLGQQYSVV      1214

sp|Q9V8R9|EPB41_DROME               KKIGLAFNYAPGNENALKETAEKLKA----------------GQLSPRTQDKLNRGQLSP      646
```

```
tr|A0A0B4LFX4|A0A0B4LFX4_DROME      KKIGLAFNYAPGNENALKETAEKLKA----------------GQLSPRTQDKLNRGQLSP        656
tr|A0A0B4LG23|A0A0B4LG23_DROME      KKIGLAFNYAPGNENALKETAEKLKA----------------GQLSPRTQDKLNRGQLSP        646
GCXY01047324.1,                     ------------------------------------------------------------        343
GCXY01047324.2,                     ------------------------------------------------------------        0
tr|W4VRQ9|W4VRQ9_9DIPT              RATGLAFNYAPGEEKNLRESIEKRKS----------------P------------EELSP        643
tr|S4NVH6|S4NVH6_9NEOP              ------------------------------------------------------------        81
tr|S4P6G9|S4P6G9_9NEOP              ------------------------------------------------------------        119
tr|S4PZT5|S4PZT5_9NEOP              ------------------------------------------------------------        73
tr|B1GS95|B1GS95_COTCN              ------------------------------------------------------------        126
tr|G1K0N0|G1K0N0_RHOPR              ------------------------------------------------------------        114
tr|A0A0L7KHN0|A0A0L7KHN0_9NEOP      ------------------------------------------------------------        279
tr|A0A0L7L3N5|A0A0L7L3N5_9NEOP      DKPKDSFKSVPGREVQLVIITCKYDAKNKKLDNPNGHIETSRGIIAADGRVHTNYGIIDP        1393
tr|A0A212EJP2|A0A212EJP2_DANPL      NKPKDTFASIPGKEVQLVIITNKYDHKYKRLDNPNGHIETSRGIVAADGRVHSNFGILDP        1274

sp|Q9V8R9|EPB41_DROME               KS----------------------------RAKLLQDPLLSPTTRAKL-----QGSAVDA        673
tr|A0A0B4LFX4|A0A0B4LFX4_DROME      KS----------------------------RAKLLQDPLLSPTTRAKL-----QGSAVDA        683
tr|A0A0B4LG23|A0A0B4LG23_DROME      KS----------------------------RAKLLQDPLLSPTTRAKL-----QGSAVDA        673
GCXY01047324.1,                     ------------------------------------------------------------        343
GCXY01047324.2,                     ------------------------------------------------------------        0
tr|W4VRQ9|W4VRQ9_9DIPT              KS----------------------------KEKFLKGGNLSPKSANKF-----ITTDVES        670
tr|S4NVH6|S4NVH6_9NEOP              ------------------------------------------------------------        81
tr|S4P6G9|S4P6G9_9NEOP              ------------------------------------------------------------        119
tr|S4PZT5|S4PZT5_9NEOP              ------------------------------------------------------------        73
tr|B1GS95|B1GS95_COTCN              ------------------------------------------------------------        126
tr|G1K0N0|G1K0N0_RHOPR              ------------------------------------------------------------        114
tr|A0A0L7KHN0|A0A0L7KHN0_9NEOP      ------------------------------------------------------------        279
tr|A0A0L7L3N5|A0A0L7L3N5_9NEOP      KSGKIEHIDPKTGKTEVKHATADPGLGFKPGTLILTSGVVDPQTGKVDSSLAQQLTIVEK        1453
tr|A0A212EJP2|A0A212EJP2_DANPL      KTGKIEKVDPVTGNSDVKNAIADP----KTGHLILTSGVVDPKTGKVDTSLAQQINIVDK        1330

sp|Q9V8R9|EPB41_DROME               AAVPL------------SDSQKRSYSPTKGPQGYSSGAPGS----------YKPI-----        706
tr|A0A0B4LFX4|A0A0B4LFX4_DROME      AAVPL------------SDSQKRSYSPTKGPQGYSSGAPGS----------YKPI-----        716
tr|A0A0B4LG23|A0A0B4LG23_DROME      AAVPL------------SDSQKRSYSPTKGPQGYSSGAPGS----------YKPI-----        706
GCXY01047324.1,                     ------------------------------------------------------------        343
GCXY01047324.2,                     ------------------------------------------------------------        0
tr|W4VRQ9|W4VRQ9_9DIPT              E---K------------AKATGKSYSPNSGKPIETPGTPGT----------YRSL-----        700
tr|S4NVH6|S4NVH6_9NEOP              ------------------------------------------------------------        81
tr|S4P6G9|S4P6G9_9NEOP              ------------------------------------------------------------        119
tr|S4PZT5|S4PZT5_9NEOP              ------------------------------------------------------------        73
tr|B1GS95|B1GS95_COTCN              ------------------------------------------------------------        126
tr|G1K0N0|G1K0N0_RHOPR              ------------------------------------------------------------        114
tr|A0A0L7KHN0|A0A0L7KHN0_9NEOP      ------------------------------------------------------------        279
tr|A0A0L7L3N5|A0A0L7L3N5_9NEOP      ETKPVEREIHLVIITTKYDPRTKKIDPSQGHVDTVTGTLGPDGKIRTAVGIIDPATGEIL        1513
tr|A0A212EJP2|A0A212EJP2_DANPL      NAKPVEREIHLVIITTKYDPRTKKIDPTQGTVDTVSGTVGPDGKIRTEFGTVDPATGEIT        1390
```

```
sp|Q9V8R9|EPB41_DROME              -SDP--------------------------------------------TADFLES-----    716
tr|A0A0B4LFX4|A0A0B4LFX4_DROME     -SDP--------------------------------------------TADFLES-----    726
tr|A0A0B4LG23|A0A0B4LG23_DROME     -SDP--------------------------------------------TADFLES-----    716
GCXY01047324.1,                    ------------------------------------------------------------    343
GCXY01047324.2,                    ------------------------------------------------------------    0
tr|W4VRQ9|W4VRQ9_9DIPT             -GDQ--------------------------------------------SKPAATE-----    710
tr|S4NVH6|S4NVH6_9NEOP             ------------------------------------------------------------    81
tr|S4P6G9|S4P6G9_9NEOP             ------------------------------------------------------------    119
tr|S4PZT5|S4PZT5_9NEOP             ------------------------------------------------------------    73
tr|B1GS95|B1GS95_COTCN             ------------------------------------------------------------    126
tr|G1K0N0|G1K0N0_RHOPR             ------------------------------------------------------------    114
tr|A0A0L7KHN0|A0A0L7KHN0_9NEOP     ------------------------------------------------------------    279
tr|A0A0L7L3N5|A0A0L7L3N5_9NEOP     VTDPKTGKSEIKKADLNPATGHMVISSQVVDPKTGKVDPTLVQQFSIVNKPVVAHPKPAS    1573
tr|A0A212EJP2|A0A212EJP2_DANPL     FTDPKTGKQDIKKAQVDPSTGHMLVTSQVVDPKTGKVDPTLAQQYSIVNKPVVAHAKPPA    1450

sp|Q9V8R9|EPB41_DROME              ------------------------QRYNKEPGYVGPS-----------------------    729
tr|A0A0B4LFX4|A0A0B4LFX4_DROME     ------------------------QRYNKEPGYVGPS-----------------------    739
tr|A0A0B4LG23|A0A0B4LG23_DROME     ------------------------QRYNKEPGYVGPS-----------------------    729
GCXY01047324.1,                    ------------------------------------------------------------    343
GCXY01047324.2,                    ------------------------------------------------------------    0
tr|W4VRQ9|W4VRQ9_9DIPT             ------------------------QKPQQQQ-----------------------------    717
tr|S4NVH6|S4NVH6_9NEOP             ------------------------------------------------------------    81
tr|S4P6G9|S4P6G9_9NEOP             ------------------------------------------------------------    119
tr|S4PZT5|S4PZT5_9NEOP             ------------------------------------------------------------    73
tr|B1GS95|B1GS95_COTCN             ------------------------------------------------------------    126
tr|G1K0N0|G1K0N0_RHOPR             ------------------------------------------------------------    114
tr|A0A0L7KHN0|A0A0L7KHN0_9NEOP     ------------------------------------------------------------    279
tr|A0A0L7L3N5|A0A0L7L3N5_9NEOP     KGEVRLVIVTSKYDPYTKTVDANSGTVDAAKGYVSAEDGKIHTDFGIIDPRSGQILYKDP    1633
tr|A0A212EJP2|A0A212EJP2_DANPL     KGEVRLVIITSKFDPKSKTVDAGAGTVDASKGYVSAEDGKIHTDFGIIDPKSGQILFKDP    1510

sp|Q9V8R9|EPB41_DROME              ------------------------KADVAAGLAG--------------------------    739
tr|A0A0B4LFX4|A0A0B4LFX4_DROME     ------------------------KADVAAGLAG--------------------------    749
tr|A0A0B4LG23|A0A0B4LG23_DROME     ------------------------KADVAAGLAG--------------------------    739
GCXY01047324.1,                    ------------------------------------------------------------    343
GCXY01047324.2,                    ------------------------------------------------------------    0
tr|W4VRQ9|W4VRQ9_9DIPT             -------------------------QQPAGGLV---------------------------    725
tr|S4NVH6|S4NVH6_9NEOP             ------------------------------------------------------------    81
tr|S4P6G9|S4P6G9_9NEOP             ------------------------------------------------------------    119
tr|S4PZT5|S4PZT5_9NEOP             ------------------------------------------------------------    73
tr|B1GS95|B1GS95_COTCN             ------------------------------------------------------------    126
tr|G1K0N0|G1K0N0_RHOPR             ------------------------------------------------------------    114
tr|A0A0L7KHN0|A0A0L7KHN0_9NEOP     ------------------------------------------------------------    279
tr|A0A0L7L3N5|A0A0L7L3N5_9NEOP     LTGKQELKQAEIDPKTRNLIVTTAVVDPATGKVDQSFAQQLTIVDKQNVLKPVPPPISQR    1693
tr|A0A212EJP2|A0A212EJP2_DANPL     VTGKQELKQADIDPKTGHITVTSSVVDPKTGKVDSSFAQQLVIVDKQNVMAKVVP---QR    1567
```

```
sp|Q9V8R9|EPB41_DROME                 --AAGSKKPGSPTKTGKGAPGA---------------------------AAAAAAGAAGA        770
tr|A0A0B4LFX4|A0A0B4LFX4_DROME        --AAGSKKPGSPTKTGKGAPGA---------------------------AAAAAAGAAGA        780
tr|A0A0B4LG23|A0A0B4LG23_DROME        --AAGSKKPGSPTKTGKGAPGA---------------------------AAAAAAGAAGA        770
GCXY01047324.1,                       ------------------------------------------------------------        343
GCXY01047324.2,                       ------------------------------------------------------------        0
tr|W4VRQ9|W4VRQ9_9DIPT                ---------------TKIDPNA---------------------------AFIAGEQFAAN        743
tr|S4NVH6|S4NVH6_9NEOP                ------------------------------------------------------------        81
tr|S4P6G9|S4P6G9_9NEOP                ------------------------------------------------------------        119
tr|S4PZT5|S4PZT5_9NEOP                ------------------------------------------------------------        73
tr|B1GS95|B1GS95_COTCN                ------------------------------------------------------------        126
tr|G1K0N0|G1K0N0_RHOPR                ------------------------------------------------------------        114
tr|A0A0L7KHN0|A0A0L7KHN0_9NEOP        ------------------------------------------------------------        279
tr|A0A0L7L3N5|A0A0L7L3N5_9NEOP        VSVSPAKQVPSPVKTPTPTPTQTPLQSPVRTSSPIISSFAKSSPLPSPVHKTTPVKPTMA        1753
tr|A0A212EJP2|A0A212EJP2_DANPL        ASASPARQTPSPIKTPNTTPVHTPLQTPVRTTAPVIGHLQKASPVT-PVRAVTPAKPTSA        1626


sp|Q9V8R9|EPB41_DROME                 AAAAAKPKKRRVKIMVITSKFDPSTKRID-AENGSIEHSTG-ILDPATGLIDTKYGVIDP        828
tr|A0A0B4LFX4|A0A0B4LFX4_DROME        AAAAAKPKKRRVKIMVITSKFDPSTKRID-AENGSIEHSTG-ILDPATGLIDTKYGVIDP        838
tr|A0A0B4LG23|A0A0B4LG23_DROME        AAAAAKPKKRRVKIMVITSKFDPSTKRID-AENGSIEHSTG-ILDPATGLIDTKYGVIDP        828
GCXY01047324.1,                       ------------------------------------------------------------        343
GCXY01047324.2,                       ------------------------------------------------------------        0
tr|W4VRQ9|W4VRQ9_9DIPT                VPPAAEPLKKKVKIMVVISKIDPKTKRIVDTSNGTVEHSTG-VLDTSTGQIESKYGLIDP        802
tr|S4NVH6|S4NVH6_9NEOP                ------------------------------------------------------------        81
tr|S4P6G9|S4P6G9_9NEOP                ------------------------------------------------------------        119
tr|S4PZT5|S4PZT5_9NEOP                ------------------------------------------------------------        73
tr|B1GS95|B1GS95_COTCN                ------------------------------------------------------------        126
tr|G1K0N0|G1K0N0_RHOPR                ------------------------------------------------------------        114
tr|A0A0L7KHN0|A0A0L7KHN0_9NEOP        ------------------------------------------------------------        279
tr|A0A0L7L3N5|A0A0L7L3N5_9NEOP        PPEPPTPKRKIVKIMVIFTKIDPKSKKPD-LHTAEVEHLTG-ILDP-NGQIETKYGVINS        1810
tr|A0A212EJP2|A0A212EJP2_DANPL        PPEP--PRRKMVKIMIIFTRLDPKTKKPD-FVTADVEHLAG-VLDP-NGLVETKYGVIDT        1681
                                                                               *

sp|Q9V8R9|EPB41_DROME                 KKGTLEALNTKTGKKEVFQG----DVDGKTG-NLHLVSGVADPKTGRLDDTLG-QIVCIT        882
tr|A0A0B4LFX4|A0A0B4LFX4_DROME        KKGTLEALNTKTGKKEVFQG----DVDGKTG-NLHLVSGVADPKTGRLDDTLG-QIVCIT        892
tr|A0A0B4LG23|A0A0B4LG23_DROME        KKGTLEALNTKTGKKEVFQG----DVDGKTG-NLHLVSGVADPKTGRLDDTLG-QIVCIT        882
GCXY01047324.1,                       ------------------------------------------------------------        343
GCXY01047324.2,                       ------------------------------------------------------------        0
tr|W4VRQ9|W4VRQ9_9DIPT                KAGTVQNLNPRTGQNEVFQG----HTDPKTG-HIYLTSGVQDPNNPSKIDDTLGQIISIA        857
tr|S4NVH6|S4NVH6_9NEOP                ------------------------------------------------------------        81
tr|S4P6G9|S4P6G9_9NEOP                ------------------------------------------------------------        119
tr|S4PZT5|S4PZT5_9NEOP                ------------------------------------------------------------        73
tr|B1GS95|B1GS95_COTCN                ------------------------------------------------------------        126
tr|G1K0N0|G1K0N0_RHOPR                ------------------------------------------------------------        114
tr|A0A0L7KHN0|A0A0L7KHN0_9NEOP        ------------------------------------------------------------        279
tr|A0A0L7L3N5|A0A0L7L3N5_9NEOP        RTGHIVITD-QAGQKQTKEGYLLSETG-----QIFINSGVIDPKTGKIDP-HLGMIMSVA        1863
tr|A0A212EJP2|A0A212EJP2_DANPL        KKGNIVVTD-SAGQKLTKDGLILNETG-----QIFINSGAIDPKTGKIDP-NMGMIVSMA        1734
```

```
                                                    *

sp|Q9V8R9|EPB41_DROME                 PQDNPVVELTVITSRIDPATGKIDTVNGDVERSLGVLNLDTGLLDTKYGEINTRTGELKA        942
tr|A0A0B4LFX4|A0A0B4LFX4_DROME        PQDNPVVELTVITSRIDPATGKIDTVNGDVERSLGVLNLDTGLLDTKYGEINTRTGELKA        952
tr|A0A0B4LG23|A0A0B4LG23_DROME        PQDNPVVELTVITSRIDPATGKIDTVNGDVERSLGVLNLDTGLLDTKYGEINTRTGELKA        942
GCXY01047324.1,                       ------------------------------------------------------------        343
GCXY01047324.2,                       ------------------------------------------------------------        0
tr|W4VRQ9|W4VRQ9_9DIPT                PDDQSIVEVTTITSKLDPQTGKVDHVNGDVERTRGILNTKTGIIKTKYGDINTKTGEVKV        917
tr|S4NVH6|S4NVH6_9NEOP                ------------------------------------------------------------        81
tr|S4P6G9|S4P6G9_9NEOP                ------------------------------------------------------------        119
tr|S4PZT5|S4PZT5_9NEOP                ------------------------------------------------------------        73
tr|B1GS95|B1GS95_COTCN                ------------------------------------------------------------        126
tr|G1K0N0|G1K0N0_RHOPR                ------------------------------------------------------------        114
tr|A0A0L7KHN0|A0A0L7KHN0_9NEOP        ------------------------------------------------------------        279
tr|A0A0L7L3N5|A0A0L7L3N5_9NEOP        KQDDPVVEITTITGPINERTGKVNIEEGTVELTKGKVDADTGHISTKYGVIDPSN--GVI        1921
tr|A0A212EJP2|A0A212EJP2_DANPL        KQDDPVVEITTITGPVDAVTGKIHIENGVVEHTKGKVDAETGNISTKYGVIDPAN--GVI        1792

sp|Q9V8R9|EPB41_DROME                 IDPKSGKIVVSKNVKVDPGTGQITILGIVDPKTNKIDPNQGRLIEVGQQIDPIVEVTSLA        1002
tr|A0A0B4LFX4|A0A0B4LFX4_DROME        IDPKSGKIVVSKNVKVDPGTGQITILGIVDPKTNKIDPNQGRLIEVGQQIDPIVEVTSLA        1012
tr|A0A0B4LG23|A0A0B4LG23_DROME        IDPKSGKIVVSKNVKVDPGTGQITILGIVDPKTNKIDPNQGRLIEVGQQIDPIVEVTSLA        1002
GCXY01047324.1,                       ------------------------------------------------------------        343
GCXY01047324.2,                       ------------------------------------------------------------        0
tr|W4VRQ9|W4VRQ9_9DIPT                TDPKTHKIVSKDGKLDKVSG-QIQITGVADPKSNKIDPNQAHLVAIGNQIDPVVEVTSVL        976
tr|S4NVH6|S4NVH6_9NEOP                ------------------------------------------------------------        81
tr|S4P6G9|S4P6G9_9NEOP                ------------------------------------------------------------        119
tr|S4PZT5|S4PZT5_9NEOP                ------------------------------------------------------------        73
tr|B1GS95|B1GS95_COTCN                ------------------------------------------------------------        126
tr|G1K0N0|G1K0N0_RHOPR                ------------------------------------------------------------        114
tr|A0A0L7KHN0|A0A0L7KHN0_9NEOP        ------------------------------------------------------------        279
tr|A0A0L7L3N5|A0A0L7L3N5_9NEOP        FVSDTS---DTKPVEIDENNGQITIRGVVDPKTAKYDANLGQVIVVGTHIDPVVEVTTFV        1978
tr|A0A212EJP2|A0A212EJP2_DANPL        FVTDTLGTQDAKSIQIDENNGQITVIGVTDPKTGKIDPKLGQVLVVGSHIDPVVEVTTFV        1852

sp|Q9V8R9|EPB41_DROME                 GKFDSKRNIIDPKTAQVETSGGQFDPKAGKIDTKYGQIDLVKHTITFNDPKSGKTVTRDI        1062
tr|A0A0B4LFX4|A0A0B4LFX4_DROME        GKFDSKRNIIDPKTAQVETSGGQFDPKAGKIDTKYGQIDLVKHTITFNDPKSGKTVTRDI        1072
tr|A0A0B4LG23|A0A0B4LG23_DROME        GKFDSKRNIIDPKTAQVETSGGQFDPKAGKIDTKYGQIDLVKHTITFNDPKSGKTVTRDI        1062
GCXY01047324.1,                       ------------------------------------------------------------        343
GCXY01047324.2,                       ------------------------------------------------------------        0
tr|W4VRQ9|W4VRQ9_9DIPT                GKIDKKGVVDP-KTISYDKSTGQLDTENGKINTKFGQLDLNKQTLTYVDPKSGKTETKDV        1035
tr|S4NVH6|S4NVH6_9NEOP                ------------------------------------------------------------        81
tr|S4P6G9|S4P6G9_9NEOP                ------------------------------------------------------------        119
tr|S4PZT5|S4PZT5_9NEOP                ------------------------------------------------------------        73
tr|B1GS95|B1GS95_COTCN                ------------------------------------------------------------        126
tr|G1K0N0|G1K0N0_RHOPR                ------------------------------------------------------------        114
tr|A0A0L7KHN0|A0A0L7KHN0_9NEOP        ------------------------------------------------------------        279
tr|A0A0L7L3N5|A0A0L7L3N5_9NEOP        GKVDSKKGLIEPKHSLIESTTGQFNPENNKIDTKYGQIDLVKGTVTYNDPKTGKFESRDL        2038
```

```
tr|A0A212EJP2|A0A212EJP2_DANPL      GKLDNKKGIIEPKHSVIESTTGQLNPDNNIINTKYGQIDLVKGTVTYNDPKTGRFESKEF        1912


sp|Q9V8R9|EPB41_DROME               KIEPTTGQIVLK----NQVNPKNNKPDKDYARIISLRIVQQRVDPATKAPITEVSASKDK        1118
tr|A0A0B4LFX4|A0A0B4LFX4_DROME      KIEPTTGQIVLK----NQVNPKNNKPDKDYARIISLRIVQQRVDPATKAPITEVSASKDK        1128
tr|A0A0B4LG23|A0A0B4LG23_DROME      KIEPTTGQIVLK----NQVNPKNNKPDKDYARIISLRIVQQRVDPATKAPITEVSASKDK        1118
GCXY01047324.1,                     ------------------------------------------------------------        343
GCXY01047324.2,                     ------------------------------------------------------------        0
tr|W4VRQ9|W4VRQ9_9DIPT              KVDPTTGQITLK----NQINPKTNKPDKDFGRIISIRIVQNRIDPVSGKQISN---IVDK        1088
tr|S4NVH6|S4NVH6_9NEOP              ------------------------------------------------------------        81
tr|S4P6G9|S4P6G9_9NEOP              ------------------------------------------------------------        119
tr|S4PZT5|S4PZT5_9NEOP              ------------------------------------------------------------        73
tr|B1GS95|B1GS95_COTCN              ------------------------------------------------------------        126
tr|G1K0N0|G1K0N0_RHOPR              ------------------------------------------------------------        114
tr|A0A0L7KHN0|A0A0L7KHN0_9NEOP      ------------------------------------------------------------        279
tr|A0A0L7L3N5|A0A0L7L3N5_9NEOP      KVDPVTGQFLLR---TGQVNPKSGKPDKDIGRLICLRIIRNKVDPISGKQIVS---NDPK        2092
tr|A0A212EJP2|A0A212EJP2_DANPL      KVDPVTGQFLLR---SGQVNPKSGKPDKDIGRLICLRIIQTKVDPVSGKQIVS---NDPK        1966
                                                ***

sp|Q9V8R9|EPB41_DROME               DIVVDPKS----NQIWVPTGATDPATKEQQYISSSVDPKTGYVITIYGYLDPKTNEIKKQ        1174
tr|A0A0B4LFX4|A0A0B4LFX4_DROME      DIVVDPKS----NQIWVPTGATDPATKEQQYISSSVDPKTGYVITIYGYLDPKTNEIKKQ        1184
tr|A0A0B4LG23|A0A0B4LG23_DROME      DIVVDPKS----NQIWVPTGATDPATKEQQYISSSVDPKTGYVITIYGYLDPKTNEIKKQ        1174
GCXY01047324.1,                     ------------------------------------------------------------        343
GCXY01047324.2,                     ------------------------------------------------------------        0
tr|W4VRQ9|W4VRQ9_9DIPT              DIKVDPKTN----QIWLPDS-KDPKSGETIYSTSQVDPKTGYVITIYGYLNPKTNEIEKQ        1143
tr|S4NVH6|S4NVH6_9NEOP              ------------------------------------------------------------        81
tr|S4P6G9|S4P6G9_9NEOP              ------------------------------------------------------------        119
tr|S4PZT5|S4PZT5_9NEOP              ------------------------------------------------------------        73
tr|B1GS95|B1GS95_COTCN              ------------------------------------------------------------        126
tr|G1K0N0|G1K0N0_RHOPR              ------------------------------------------------------------        114
tr|A0A0L7KHN0|A0A0L7KHN0_9NEOP      ------------------------------------------------------------        279
tr|A0A0L7L3N5|A0A0L7L3N5_9NEOP      NVKVDPKT----NQIWIAG-PKDPQTGEVLYTAGQIDPITGYIITIYGRLDPKTGVIVRT        2147
tr|A0A212EJP2|A0A212EJP2_DANPL      NVKVDPKT----NQIWIAG-PKDPQTGEVLYTAGQIDPNTGYIITIYGRLDPKTGTITRA        2021
                                             ***

sp|Q9V8R9|EPB41_DROME               TKLDPNTIKIEPTSGKIYTATGEVDQATGEPLYAATQVDPESGEVYTKLARVDPKTGKIV        1234
tr|A0A0B4LFX4|A0A0B4LFX4_DROME      TKLDPNTIKIEPTSGKIYTATGEVDQATGEPLYAATQVDPESGEVYTKLARVDPKTGKIV        1244
tr|A0A0B4LG23|A0A0B4LG23_DROME      TKLDPNTIKIEPTSGKIYTATGEVDQATGEPLYAATQVDPESGEVYTKLARVDPKTGKIV        1234
GCXY01047324.1,                     ------------------------------------------------------------        343
GCXY01047324.2,                     ------------------------------------------------------------        0
tr|W4VRQ9|W4VRQ9_9DIPT              TKLDPNLTKVDPTNGQIYSATGQVDEKTGEPLFATSEINDETGEIYTKVGKVDPKTGKLV        1203
tr|S4NVH6|S4NVH6_9NEOP              ------------------------------------------------------------        81
tr|S4P6G9|S4P6G9_9NEOP              ------------------------------------------------------------        119
tr|S4PZT5|S4PZT5_9NEOP              ------------------------------------------------------------        73
tr|B1GS95|B1GS95_COTCN              ------------------------------------------------------------        126
tr|G1K0N0|G1K0N0_RHOPR              ------------------------------------------------------------        114
tr|A0A0L7KHN0|A0A0L7KHN0_9NEOP      ------------------------------------------------------------        279
```

```
tr|A0A0L7L3N5|A0A0L7L3N5_9NEOP      TEVDKSLIKVDPVNGQIYTATGEVDENN-EPLYSASQVDPGTGEIYTKLGKIDARTGRLI        2206
tr|A0A212EJP2|A0A212EJP2_DANPL      TDIDKALIKIDPINGQIYTATGDVDEDN-EPLYSASQVDPGSGEIYTKLGKIDPKTGKLI        2080

sp|Q9V8R9|EPB41_DROME               IVRILLISKTDERGRPEEIDPSTCEIDPVSGRVLKFFNKTVYVYNMIDPVTGEIVQVDPN        1294
tr|A0A0B4LFX4|A0A0B4LFX4_DROME      IVRILLISKTDERGRPEEIDPSTCEIDPVSGRVLKFFNKTVYVYNMIDPVTGEIVQVDPN        1304
tr|A0A0B4LG23|A0A0B4LG23_DROME      IVRILLISKTDERGRPEEIDPSTCEIDPVSGRVLKFFNKTVYVYNMIDPVTGEIVQVDPN        1294
GCXY01047324.1,                     ------------------------------------------------------------        343
GCXY01047324.2,                     ------------------------------------------------------------        0
tr|W4VRQ9|W4VRQ9_9DIPT              IIKIFILTKKDERGKPEEVDVNSCDIDPESGKIRSIATKTVYVYKMRDPITGETYKIDSN        1263
tr|S4NVH6|S4NVH6_9NEOP              ------------------------------------------------------------        81
tr|S4P6G9|S4P6G9_9NEOP              ------------------------------------------------------------        119
tr|S4PZT5|S4PZT5_9NEOP              ------------------------------------------------------------        73
tr|B1GS95|B1GS95_COTCN              ------------------------------------------------------------        126
tr|G1K0N0|G1K0N0_RHOPR              ------------------------------------------------------------        114
tr|A0A0L7KHN0|A0A0L7KHN0_9NEOP      ------------------------------------------------------------        279
tr|A0A0L7L3N5|A0A0L7L3N5_9NEOP      IIKIFVITQKDDKGRVKEVDPKECTIDETTGRIITTK--TVYLYQIIDPITGETIDVDPD        2264
tr|A0A212EJP2|A0A212EJP2_DANPL      IIRIYVITQKDDKGRVKELDPKECTIDETTGRIITTK--TVYVYQIIDPITGETIDVDPD        2138

sp|Q9V8R9|EPB41_DROME               DPRFAGARTTVTHTMTLTGEIDPVTGRIKSEYGDIDPNTGDIDPATAVTDPVTGKLILNY        1354
tr|A0A0B4LFX4|A0A0B4LFX4_DROME      DPRFAGARTTVTHTMTLTGEIDPVTGRIKSEYGDIDPNTGDIDPATAVTDPVTGKLILNY        1364
tr|A0A0B4LG23|A0A0B4LG23_DROME      DPRFAGARTTVTHTMTLTGEIDPVTGRIKSEYGDIDPNTGDIDPATAVTDPVTGKLILNY        1354
GCXY01047324.1,                     ------------------------------------------------------------        343
GCXY01047324.2,                     ------------------------------------------------------------        0
tr|W4VRQ9|W4VRQ9_9DIPT              DPSIAGARTTVTETMTLSGEIDPVTGRIKTEWGNIDPNTGDIDPETAIRDPVTGKLILNY        1323
tr|S4NVH6|S4NVH6_9NEOP              ------------------------------------------------------------        81
tr|S4P6G9|S4P6G9_9NEOP              ------------------------------------------------------------        119
tr|S4PZT5|S4PZT5_9NEOP              ------------------------------------------------------------        73
tr|B1GS95|B1GS95_COTCN              ------------------------------------------------------------        126
tr|G1K0N0|G1K0N0_RHOPR              ------------------------------------------------------------        114
tr|A0A0L7KHN0|A0A0L7KHN0_9NEOP      ------------------------------------------------------------        279
tr|A0A0L7L3N5|A0A0L7L3N5_9NEOP      DPRLKGARTTVTQTMTLSGKIDPVTGRIKTEYGDIDPDTGDIDPSTAVRDPVTGQLILHY        2324
tr|A0A212EJP2|A0A212EJP2_DANPL      DPRLKGARTTVTQTMTLSGKIDPITGRIKTEYGDIDPDTGDIDPSTAVRDPVTGQLILHY        2198

sp|Q9V8R9|EPB41_DROME               AQIDPSHFGKQAQVQ-TTTETVPITRQQFFDGV-----KHISKG----------ALRRDS        1398
tr|A0A0B4LFX4|A0A0B4LFX4_DROME      AQIDPSHFGKQAQVQ-TTTETVPITRQQFFDGV-----KHISKG----------ALRRDS        1408
tr|A0A0B4LG23|A0A0B4LG23_DROME      AQIDPSHFGKQAQVQ-TTTETVPITRQQFFDGV-----KHISKG----------ALRRDS        1398
GCXY01047324.1,                     ------------------------------------------------------------        343
GCXY01047324.2,                     ------------------------------------------------------------        0
tr|W4VRQ9|W4VRQ9_9DIPT              ADIEPSHFG--KNVT-VTKETVPITKQQFYDGI-----KHLGKK----------AIRRDS        1365
tr|S4NVH6|S4NVH6_9NEOP              ------------------------------------------------------------        81
tr|S4P6G9|S4P6G9_9NEOP              ------------------------------------------------------------        119
tr|S4PZT5|S4PZT5_9NEOP              ------------------------------------------------------------        73
tr|B1GS95|B1GS95_COTCN              ------------------------------------------------------------        126
tr|G1K0N0|G1K0N0_RHOPR              ------------------------------------------------------------        114
```

```
tr|A0A0L7KHN0|A0A0L7KHN0_9NEOP      ------------------------------------------------------------      279
tr|A0A0L7L3N5|A0A0L7L3N5_9NEOP      SQIDPSHFEDKSGNYTIEKETEDLPANIDIQTVNTHKFSTFGKDES--------PQRG--      2374
tr|A0A212EJP2|A0A212EJP2_DANPL      SQIDPSHFEDKSGNYTIEKETHDLPANIDIQTVNTHKFSTFGKDES--------PARG--      2248
                                                                                  ********

sp|Q9V8R9|EPB41_DROME               EGSSDDDMTAQYGADQV--------NEILIGS--PAGQAGGKLGKPVSTPTVVKTTTKQV      1448
tr|A0A0B4LFX4|A0A0B4LFX4_DROME      EGSSDDDMTAQYGADQV--------NEILIGS--PAGQAGGKLGKPVSTPTVVKTTTKQV      1458
tr|A0A0B4LG23|A0A0B4LG23_DROME      EGSSDDDMTAQYGADQV--------NEILIGS--PAGQAGGKLGKPVSTPTVVKTTTKQV      1448
GCXY01047324.1,                     ------------------------------------------------------------      343
GCXY01047324.2,                     ------------------------------------------------------------      0
tr|W4VRQ9|W4VRQ9_9DIPT              ETSDDETV--EYDVKEL--------SP-------------TSAAGKQGTPTVVKTTTKQV      1402
tr|S4NVH6|S4NVH6_9NEOP              ------------------------------------------------------------      81
tr|S4P6G9|S4P6G9_9NEOP              ------------------------------------------------------------      119
tr|S4PZT5|S4PZT5_9NEOP              ------------------------------------------------------------      73
tr|B1GS95|B1GS95_COTCN              ------------------------------------------------------------      126
tr|G1K0N0|G1K0N0_RHOPR              ------------------------------------------------------------      114
tr|A0A0L7KHN0|A0A0L7KHN0_9NEOP      ------------------------------------------------------------      279
tr|A0A0L7L3N5|A0A0L7L3N5_9NEOP      ---DEPKTFTEYTTSEHIRHQGYVSSNTPLSSKIPISQ---RSKKTPTPPVVVKTTTKQL      2428
tr|A0A212EJP2|A0A212EJP2_DANPL      ---DEPKTFTEYTTSEHIRHQGYASS-TPISSKIPISQ---RSKKTPTPPVVVKTTTKQL      2301


sp|Q9V8R9|EPB41_DROME               LTKNIDGVTHNVEEEVRNLGTGEVTYSTQEHKADATPT-D--LSGAYVTATAVTTRTATT      1505
tr|A0A0B4LFX4|A0A0B4LFX4_DROME      LTKNIDGVTHNVEEEVRNLGTGEVTYSTQEHK----------------------------      1490
tr|A0A0B4LG23|A0A0B4LG23_DROME      LTKNIDGVTHNVEEEVRNLGTGEVTYSTQEHKADATPT-D--LSGAYVTATAVTTRTATT      1505
GCXY01047324.1,                     ------------------------------------------------------------      343
GCXY01047324.2,                     ---L----TQNVEESIENVNTGEVTHSSHINTAEGLDE---SQT-PIITATAVTTRTATT      49
tr|W4VRQ9|W4VRQ9_9DIPT              ITKNDDGVTHNVEEEVQNLGTGQIVYSTQEHKADAPTNNA--TPGAFVSGTAITTRTATT      1460
tr|S4NVH6|S4NVH6_9NEOP              ------------------------------------------------------------      81
tr|S4P6G9|S4P6G9_9NEOP              ------------------------------------------------------------      119
tr|S4PZT5|S4PZT5_9NEOP              ------------------------------------------------------------      73
tr|B1GS95|B1GS95_COTCN              ------------------------------------------------------------      126
tr|G1K0N0|G1K0N0_RHOPR              ------------------------------------------------------------      114
tr|A0A0L7KHN0|A0A0L7KHN0_9NEOP      ------------------------------------------------------------      279
tr|A0A0L7L3N5|A0A0L7L3N5_9NEOP      LTKNDEGVTHNVEQEVENLGTGEVTFSTHTNKAESLEPMEVGGKSPYVTARAVTTRTATT      2488
tr|A0A212EJP2|A0A212EJP2_DANPL      LTKNDEGVTHNVEQEVEHLGTGEVTFSTHTNKAESLEP---EGKSPYVTARAVTTRTATT      2358


sp|Q9V8R9|EPB41_DROME               HEDLGKNAKTEQLEEKTVATTRTHDPNKQQQRVVTQEVKTTATVTSGDQYQ-RRDSVSST      1564
tr|A0A0B4LFX4|A0A0B4LFX4_DROME      ------------------------------------------------------------      1490
tr|A0A0B4LG23|A0A0B4LG23_DROME      HEDLGKNAKTEQLEEKTVATTRTHDPNKQQQRVVTQEVKTTATVTSGD--Q-RRDSVSST      1562
GCXY01047324.1,                     ------------------------------------------------------------      343
GCXY01047324.2,                     LKDLGTNAETSEVQEKTVATSTTKQGSRQEQRVVTQEVRAT-TVVNDPQIE-RSKSSSSL      107
tr|W4VRQ9|W4VRQ9_9DIPT              HEDLGTNIKTQQLEEKTVATTTTQHENRQEHKVITQEVKTTATVTSGDQFINRRESVSST      1520
tr|S4NVH6|S4NVH6_9NEOP              ------------------------------------------------------------      81
tr|S4P6G9|S4P6G9_9NEOP              ------------------------------------------------------------      119
tr|S4PZT5|S4PZT5_9NEOP              ------------------------------------------------------------      73
tr|B1GS95|B1GS95_COTCN              ------------------------------------------------------------      126
```

```
tr|G1K0N0|G1K0N0_RHOPR              ------------------------------------------------------------        114
tr|A0A0L7KHN0|A0A0L7KHN0_9NEOP      ------------------------------------------------------------        279
tr|A0A0L7L3N5|A0A0L7L3N5_9NEOP      HHDLDTKAKTQQMEERTVAQTVTSSATRQEQRVLTQEVKTM--VTTGDQVH-N-------        2538
tr|A0A212EJP2|A0A212EJP2_DANPL      HHDLDTKARTQQMEEKTVAHTVTSSATRQEQRVLTQQVKTT--VTTGDQLT-RRGSQSSL        2415


sp|Q9V8R9|EPB41_DROME               SSGDSGTPIDGPYDGASVVRT--DNQKSPLFTT---SATTG-----------PHVESTRV        1608
tr|A0A0B4LFX4|A0A0B4LFX4_DROME      --------------------------KSPLFTT---SATTG-----------PHVESTRV        1510
tr|A0A0B4LG23|A0A0B4LG23_DROME      SSGDSGTPIDGPYDGASVVRT--DNQKSPLFTT---SATTG-----------PHVESTRV        1606
GCXY01047324.1,                     ------------------------------------------------------------        343
GCXY01047324.2,                     SSDDSGTPIDGDYYPDRPQGPIVET-EKVVYSRPLGEDGMGEELRTSSPPLVPTV-SQRL        165
tr|W4VRQ9|W4VRQ9_9DIPT              SSGDSGTPIDGPYDQSNVILN--KSYTGVQEGS---NLPSG-----------PNVEQHHV        1564
tr|S4NVH6|S4NVH6_9NEOP              ------------------------------------------------------------        81
tr|S4P6G9|S4P6G9_9NEOP              ------------------------------------------------------------        119
tr|S4PZT5|S4PZT5_9NEOP              ------------------------------------------------------------        73
tr|B1GS95|B1GS95_COTCN              ------------------------------------------------------------        126
tr|G1K0N0|G1K0N0_RHOPR              ------------------------------------------------------------        114
tr|A0A0L7KHN0|A0A0L7KHN0_9NEOP      ------------------------------------------------------------        279
tr|A0A0L7L3N5|A0A0L7L3N5_9NEOP      ------------------------------------------P---------LHT-----        2542
tr|A0A212EJP2|A0A212EJP2_DANPL      SSGDSGTPIDLDEGSEGHYYTAPGSYTTTTTSSAVGNAPFGGV---------LHSASARM        2466


sp|Q9V8R9|EPB41_DROME               VLGEDTPGFSGHGEIISTQT--------VSSKTRTVETITYKTERDGIVETRVEQKITIQ        1660
tr|A0A0B4LFX4|A0A0B4LFX4_DROME      VLGEDTPGFSGHGEIISTQT--------VSSKTRTVETITYKTERDGIVETRVEQKITIQ        1562
tr|A0A0B4LG23|A0A0B4LG23_DROME      VLGEDTPGFSGHGEIISTQT--------VSSKTRTVETITYKTERDGIVETRVEQKITIQ        1658
GCXY01047324.1,                     ------------------------------------------------------------        343
GCXY01047324.2,                     ELEDGT--FSLHGTITS-QT--------ITSKTRTVETLTYKTEKDGVVETRVEQKITIQ        214
tr|W4VRQ9|W4VRQ9_9DIPT              FLDDD--GTAERGEIVSTQT--------VSSKTRTVETITYKTERDGVVETRVEQKITIQ        1614
tr|S4NVH6|S4NVH6_9NEOP              ------------------------------------------------------------        81
tr|S4P6G9|S4P6G9_9NEOP              ------------------------------------------------------------        119
tr|S4PZT5|S4PZT5_9NEOP              ------------------------------------------------------------        73
tr|B1GS95|B1GS95_COTCN              ------------------------------------------------------------        126
tr|G1K0N0|G1K0N0_RHOPR              ------------------------------------------------------------        114
tr|A0A0L7KHN0|A0A0L7KHN0_9NEOP      ------------------------------------------------------------        279
tr|A0A0L7L3N5|A0A0L7L3N5_9NEOP      ------------------------------------------------------------        2542
tr|A0A212EJP2|A0A212EJP2_DANPL      TSSPPPPSPPAEGEVVSSQT--------ISSKTRTVETITYKTERNGVVETRVEQKITIQ        2518
                                                        ********

sp|Q9V8R9|EPB41_DROME               SDGDPIDHDKALAEAIQEATAMNPDMTVEKIEIQQQTQ--    1698
tr|A0A0B4LFX4|A0A0B4LFX4_DROME      SDGDPIDHDKALAEAIQEATAMNPDMTVEKIEIQQQTQ--    1600
tr|A0A0B4LG23|A0A0B4LG23_DROME      SDGDPIDHDKALAEAIQEATAMNPDMTVEKIEIQQQTQ--    1696
GCXY01047324.1,                     ----------------------------------------    343
GCXY01047324.2,                     SDGDPIDHEKALNDAIQEATAMNPDLMVEKIEIQQE----    250
tr|W4VRQ9|W4VRQ9_9DIPT              SDGDPIDHDKALAEAIQEATAMNPDMTVEKIEIQQQTQ--    1652
tr|S4NVH6|S4NVH6_9NEOP              ----------------------------------------    81
tr|S4P6G9|S4P6G9_9NEOP              ----------------------------------------    119
tr|S4PZT5|S4PZT5_9NEOP              ----------------------------------------    73
```

```
tr|B1GS95|B1GS95_COTCN            ----------------------------------------    126
tr|G1K0N0|G1K0N0_RHOPR            ----------------------------------------    114
tr|A0A0L7KHN0|A0A0L7KHN0_9NEOP    ----------------------------------------    279
tr|A0A0L7L3N5|A0A0L7L3N5_9NEOP    ----------------------------------------    2542
tr|A0A212EJP2|A0A212EJP2_DANPL    SDGDPIDHDRALAEAIQEATAMNPDMTVEKIEIQQQSTQP    2558
```
