# Supplementary material for: The large milkweed bugs’ Na,K-ATPase β-subunits colocalize with septate junction proteins in a tissue-specific manner
Source: Cell Tissue Res. 2025 Mar 26;400(3):347–63. doi: 10.1007/s00441-025-03965-3 (PMC12125057; doi:10.1007/s00441-025-03965-3)
Supplement: Supplementary file 5 — Supplementary Material 5 (PDF 9.10 MB) [file 441_2025_3965_MOESM5_ESM.pdf]

The large milkweed bugs' Na,K-ATPase β-subunits colocalize with septate junction proteins in a tissue-specific manner

Marlena Herbertz[1*], Christian Lohr[2], Susanne Dobler[1]

[1] Institute of Cell and Systems Biology of Animals, Molecular Evolutionary Biology, Universität Hamburg, 20146 Hamburg, Germany

[2] Institute of Zell and Systems Biology of Animals, Neurophysiology, Universität Hamburg, 20146 Hamburg, Germany

*corresponding author: marlena.herbertz@uni-hamburg.de

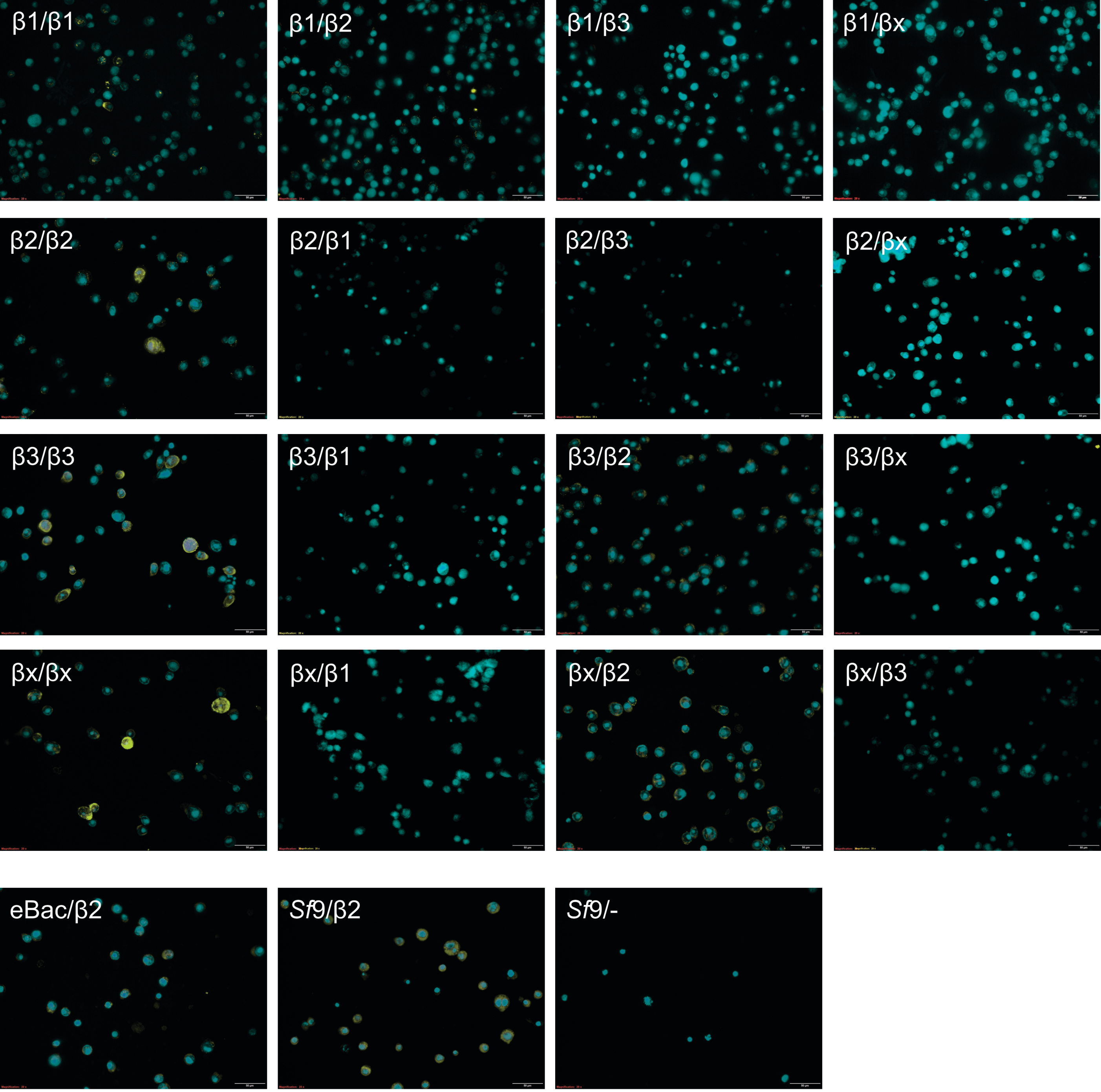

Figure S4: Immunocytochemical prove of binding specifity of NKA β-specific antibodies. The β-subunits were expressed in *Sf*9 cells and stained with different β-specific antibodies. In the first column, only suitable antibodies were use to detect the target protein (target protein / antibody). The images in the other columns show the effect of the other antibodies on an non-target protein. All β-specific antibodies only stain their target β-subunit. Additionally, anti-β2 antibody binds to endogenous β-subunits in the Sf9 cells, causing a slight signal in the cells with non-target β-subunits, cells with empty bacmids (eBac), and untransfected cells (*Sf*9). Negative control: *Sf*9 cells stained only with secondary anti-rabbit antibody (*Sf*9/-). (Yellow: β-subunits; cyan: nuclei; scale bars: 50μm)
