# Supplementary material for: The large milkweed bugs’ Na,K-ATPase β-subunits colocalize with septate junction proteins in a tissue-specific manner
Source: Cell Tissue Res. 2025 Mar 26;400(3):347–63. doi: 10.1007/s00441-025-03965-3 (PMC12125057; doi:10.1007/s00441-025-03965-3)
Supplement: Supplementary file 6 — Supplementary Material 6 (PDF 6.98 MB) [file 441_2025_3965_MOESM6_ESM.pdf]

The large milkweed bugs' Na,K-ATPase β-subunits colocalize with septate junction proteins in a tissue-specific manner

Marlena Herbertz[1*], Christian Lohr[2], Susanne Dobler[1]

[1] Institute of Cell and Systems Biology of Animals, Molecular Evolutionary Biology, Universität Hamburg, 20146 Hamburg, Germany

[2] Institute of Zell and Systems Biology of Animals, Neurophysiology, Universität Hamburg, 20146 Hamburg, Germany

*corresponding author: marlena.herbertz@uni-hamburg.de

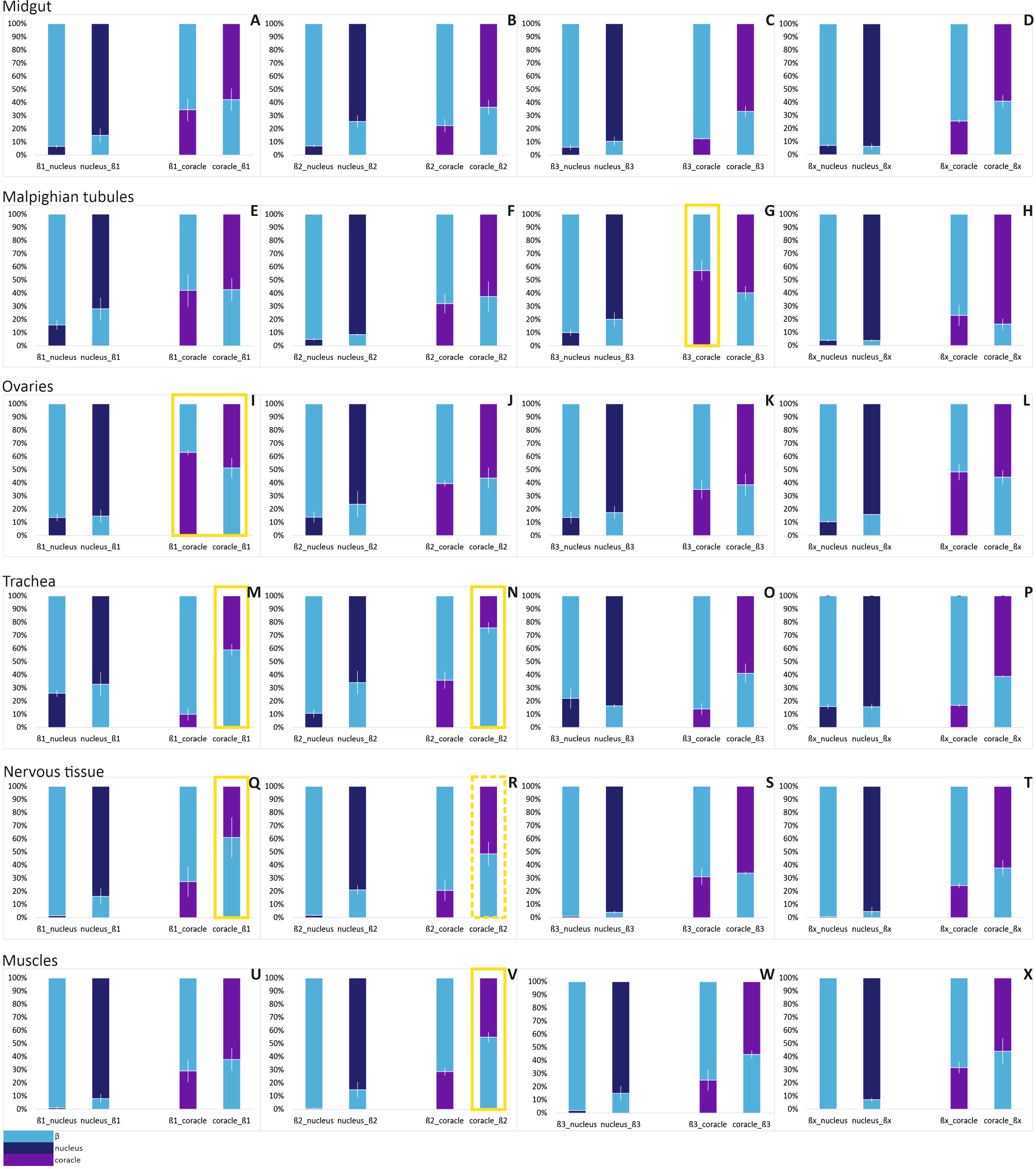

Figure S5: Proportions of colocalization (N=3, mean ± standard error) over a defined area (100%) for different pairings: nuclei (dark blue) overlap area of β-subunits (light blue), area of β-subunits overlap area of nuclei, area of coracle (violet) overlap area of β-subunits, and area of β-subunits overlap area of coracle. Areas were extracted from IHC images and were analyzed with Fiji (ImageJ, Version 1.53c). Each β-subunit is represented separately in one graph (graphs from left to right: β1, β2, β3, βx). Following tissues were analyzed: Midgut (A-D), Malpighian tubules (E-H), ovaries (I-L), trachea (M-P), nervous tissue (Q-T), and muscles (U-X). Yellow boxes highlight strong interdependences between coracle and β-subunits (overlap > 50% of the total tissue area). Box with yellow dashed line highlights a strong interdependence between coracle and β2 in the nervous tissue, where the overlap almost reached 50%.
