# Supplementary material for: The large milkweed bugs’ Na,K-ATPase β-subunits colocalize with septate junction proteins in a tissue-specific manner
Source: Cell Tissue Res. 2025 Mar 26;400(3):347–63. doi: 10.1007/s00441-025-03965-3 (PMC12125057; doi:10.1007/s00441-025-03965-3)
Supplement: Supplementary file 7 — Supplementary Material 7 (PDF 27.1 MB) [file 441_2025_3965_MOESM7_ESM.pdf]

The large milkweed bugs' Na,K-ATPase β-subunits colocalize with septate junction proteins in a tissue-specific manner

Marlena Herbertz[1*], Christian Lohr[2], Susanne Dobler[1]

[1] Institute of Cell and Systems Biology of Animals, Molecular Evolutionary Biology, Universität Hamburg, 20146 Hamburg, Germany

[2] Institute of Zell and Systems Biology of Animals, Neurophysiology, Universität Hamburg, 20146 Hamburg, Germany

*corresponding author: marlena.herbertz@uni-hamburg.de

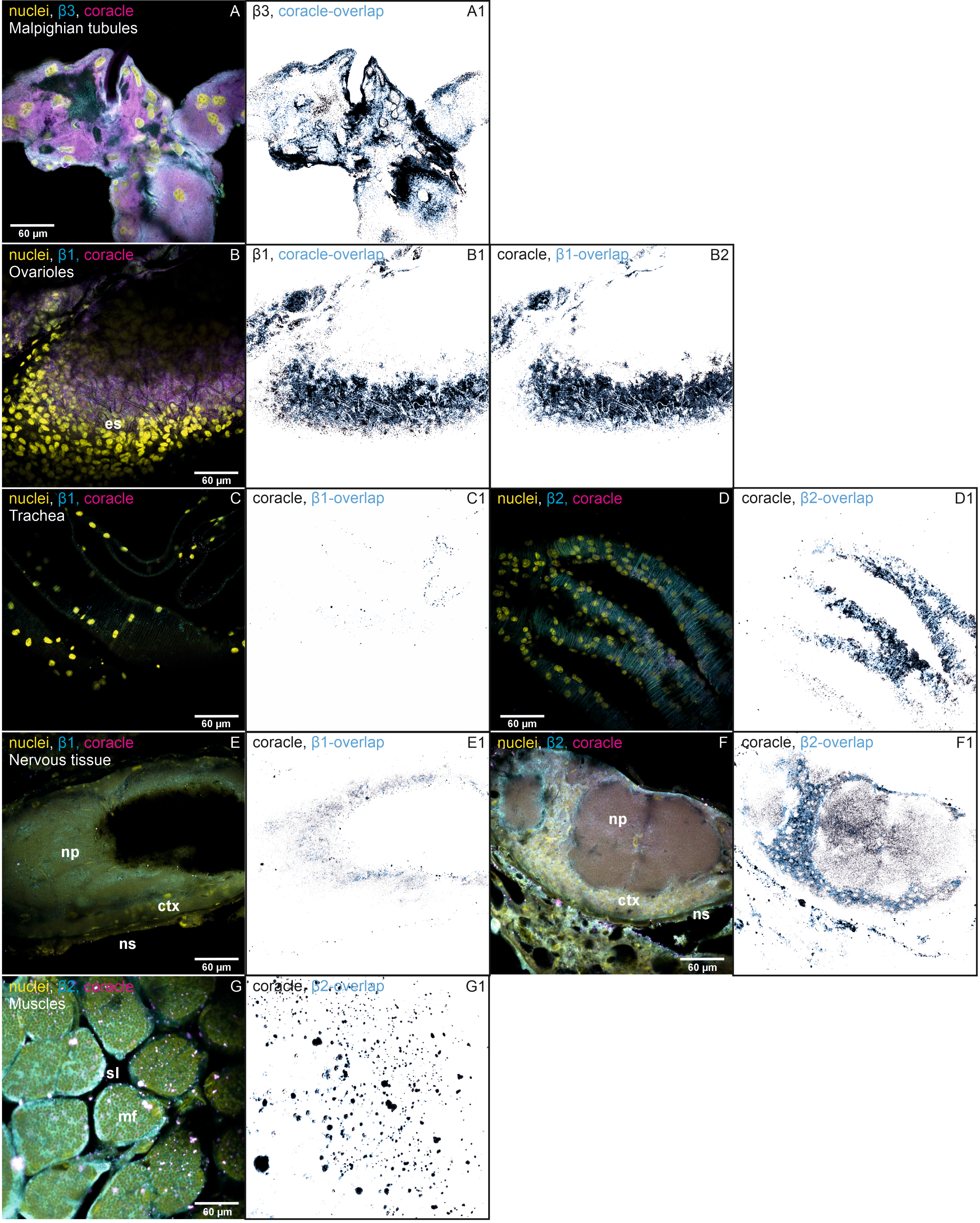

Figure S6: Immunohistochemistry (IHC) images of different tissues show immunostainings of β-subunits (cyan), coracle (magenta), nuclei (yellow), and colocalizations. One reference image out of three similar biological replicates is shown that represent overlaps of β-subunits and coralce that are equal to or higher than 50% (see Fig. S5). The following binary images in one row show calculated and extracted areas of β-subunits, coracle, and colocalization (black on white background, Fiji ImageJ Version 1.54f). (A) IHC image of Malpighian tubules: β3, coracle , and nuclei. (A1) Binary image of extracted area of β3 (black) and overlap with coracle (blue). (B) IHC image of ovarioles: β1, coracle, and nuclei. (B1) Extracted area of β1 (black) and overlap with coracle (blue). (B2) Extracted area of coracle (black) and overlap with β1 (blue). (C) IHC image of trachea: β1, coracle, and nuclei. (C1) Extracted area of coracle (black) and overlap with β1 (blue). (D) IHC image of trachea: β2, coracle, and nuclei. (D1) Extracted area of coracle (black) and overlap with β2 (blue). (E) IHC image of nervous tissue: β1, coracle, and nuclei. (E1) Extracted area of coracle (black) and overlap with β1 (blue). (F) IHC image of nervous tissue: β2, coracle, and nuclei. (F1) Extracted area of coracle (black) and overlap with β2 (blue). (G) IHC image of nervous tissue: β2, coracle, and nuclei. (G) Extracted area of coracle (black) and overlap with β2 (blue). (Scale bar 60µm, es: epithelial sheath, np: neuropil, ctx: cortex, ns: neural sheath, sl: sarcolemma, mf: muscle fiber). For negative controls see Fig. S8.
