# Supplementary material for: The large milkweed bugs’ Na,K-ATPase β-subunits colocalize with septate junction proteins in a tissue-specific manner
Source: Cell Tissue Res. 2025 Mar 26;400(3):347–63. doi: 10.1007/s00441-025-03965-3 (PMC12125057; doi:10.1007/s00441-025-03965-3)
Supplement: Supplementary file 8 — Supplementary Material 8 (PDF 2.26 MB) [file 441_2025_3965_MOESM8_ESM.pdf]

The large milkweed bugs' Na,K-ATPase β-subunits colocalize with septate junction proteins in a tissue-specific manner

Marlena Herbertz[1*], Christian Lohr[2], Susanne Dobler[1]

[1] Institute of Cell and Systems Biology of Animals, Molecular Evolutionary Biology, Universität Hamburg, 20146 Hamburg, Germany

[2] Institute of Zell and Systems Biology of Animals, Neurophysiology, Universität Hamburg, 20146 Hamburg, Germany

*corresponding author: marlena.herbertz@uni-hamburg.de

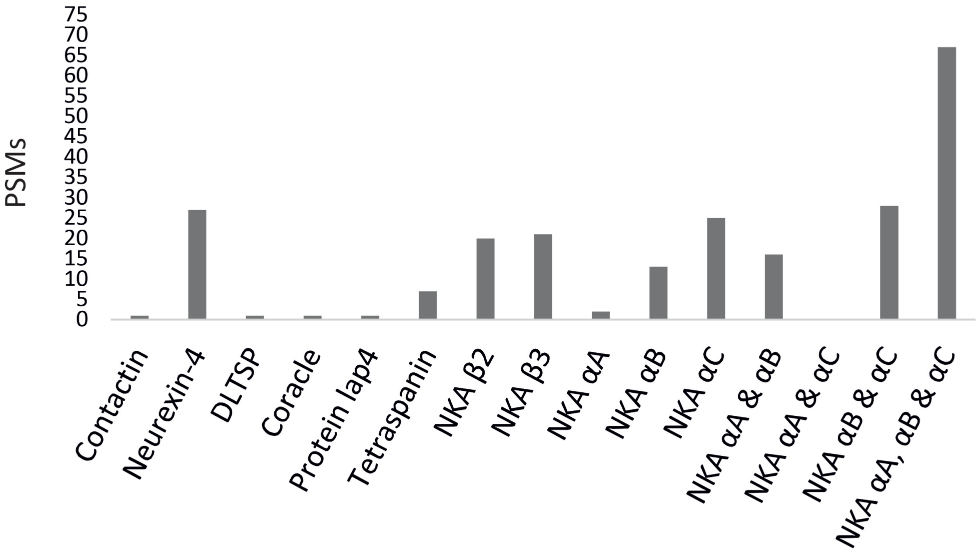

Figure S7: PSMs resulting from LC-MS/MS analyses of nervous tissue samples immunoprecipitated with magnetic beads cross linked to the anti-coracle antibody C566.9 (DSHB Hybridoma Bank) are shown for different junction proteins. The higher the number of PSMs the more likely is a high presence in the tissue, indicating a possible complex partner. Shared peptides of different NKA α1 paralogs are also indicated (last four bars).
