# Supplementary material for: The large milkweed bugs’ Na,K-ATPase β-subunits colocalize with septate junction proteins in a tissue-specific manner
Source: Cell Tissue Res. 2025 Mar 26;400(3):347–63. doi: 10.1007/s00441-025-03965-3 (PMC12125057; doi:10.1007/s00441-025-03965-3)
Supplement: Supplementary file 9 — Supplementary Material 9 (PDF 20.4 MB) [file 441_2025_3965_MOESM9_ESM.pdf]

The large milkweed bugs' Na,K-ATPase β-subunits colocalize with septate junction proteins in a tissue-specific manner

Marlena Herbertz[1*], Christian Lohr[2], Susanne Dobler[1]

[1] Institute of Cell and Systems Biology of Animals, Molecular Evolutionary Biology, Universität Hamburg, 20146 Hamburg, Germany

[2] Institute of Zell and Systems Biology of Animals, Neurophysiology, Universität Hamburg, 20146 Hamburg, Germany

*corresponding author: marlena.herbertz@uni-hamburg.de

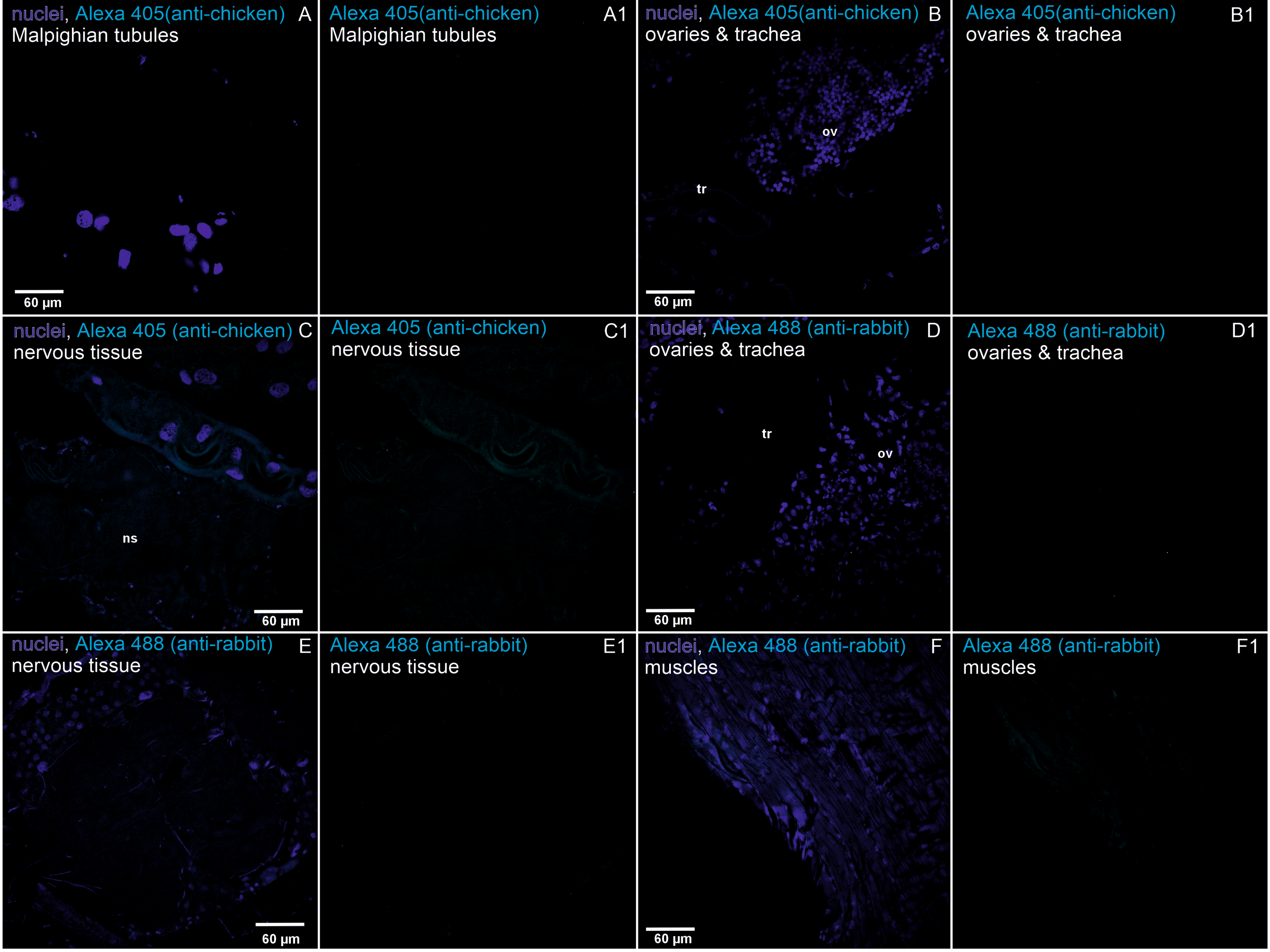

Figure S8: Immunohistochemical images of negative controls in Malpighian tubules, ovaries, trachea, nervous tissue, and muscles from *Oncopeltus fasciatus*. As negative controls tissues were exclusively stained with secondary antibodies (cyan) and Dapi (blue). (A) Malpighian tubules stained with Dapi and Alexa 405 (anti-chicken (β1 and β3)) are shown. (A1) Only Alexa 405 immunostain is shown here, the signal with equal or higher detector gains than the ones used for the main samples is negligible. (B) Ovaries and trachea stained with Dapi and Alexa 405 (anti-chicken (β1 and β3)) are shown. (B1) Only Alexa 405 immunostain is shown here, the signal was clearly weaker than signals of stainings in the main samples at same or higher detector gains. (C) Nervous tissue stained with Dapi (blue) and Alexa 405 (anti-chicken secondary antibody (β1 and β3)) is shown. (C1) Only Alexa 405 immunostain is shown here, a weak signal was recorded. The detector gain was set higher than for the main samples (D) Ovaries and trachea stained with Dapi and Alexa 488 (anti-rabbit (β2 and βx)) are shown. (D1) Only Alexa 488 immunostain is shown here, a negliglible signal was recorded after setting same or higher detector gains as for the main samples. (E) Nervous tissue stained with Dapi and Alexa 488 (anti-rabbit (β2 and βx)) is shown. (E1) Only Alexa 488 immunostain is shown here, only a weak signal was recorded. (F) Muscles stained withDapi and Alexa 488 (anti-rabbit (β2 and βx)) are shown. (F1) Only Alexa 488 immunostain is shown here, only a weak signal was recorded.The detector gain was set higher than for the main samples. (scale bars: 60µm, ov: ovaries, tr: trachea, ns: nervous tissue)
